# Cryo-EM structure of ex vivo Sup35 yeast prion and the factors defining prion phenotype

**DOI:** 10.1038/s41467-026-77823-3

**Published:** 2026-09-19

**Authors:** Alexander A. Dergalev, Yury M. Chesnokov, Anna D. Burtseva, Olga V. Mitkevich, Timur N. Baymukhametov, Vladimir O. Popov, Konstantin M. Boyko, Vitaly V. Kushnirov

**Affiliations:** 1 Bach Institute of Biochemistry, Federal Research Center of Biotechnology of the Russian Academy of Sciences, Moscow, Russia; 2 National Research Center “Kurchatov Institute”, Moscow, Russia; 3 Moscow Center for Advanced Studies, Moscow, Russia

**Keywords:** Cryoelectron microscopy, Prions

## Abstract

Human prions and amyloids exhibit structural diversity that correlates with different pathological manifestations. Yeast prions represent a convenient model for studying fundamental properties of prions and their strain diversity. The Sup35 yeast prion has multiple structural variants, divided into “strong” and “weak” groups differing in frequency of fibril fragmentation and resulting nonsense-suppression. The molecular origin of these distinctions remains unclear. Here, we show that the different frequency of prion fragmentation is determined by their stability, rather than the efficiency of chaperone binding. The fully protease-resistant part (residues 2-32) of the Sup35 prion core is a key for the prion phenotype and maintenance, whereas partially resistant part (33-72) is less important. We established the cryo-EM structure of a strong Sup35 prion variant purified from yeast, comprising residues 2–64, with 2.7 Å resolution. Comparison of this structure with that of Sup35 amyloid formed in vitro at 4˚C revealed their significant difference.

## Abstract (web summary)

Here, authors present the cryo-EM structure of a “strong” Sup35 prion purified from yeast, comprising residues 2–64. This structure differs significantly from that of Sup35 amyloid generated in vitro at 4˚C, but fits earlier proteinase K mapping data.

## Introduction

Amyloids are filamentous non-covalent protein polymers, which contain a highly ordered core with a cross-β structure and may also include structured and unstructured parts. Amyloids are primarily known as pathological agents related to some incurable degenerative diseases of mammals^1,2^ – but, as it is now known, they are widespread across all kingdoms of life, and often play an important role in the normal physiology of various organisms^3–5^. Some amyloids can infect multicellular organisms or stably propagate in microorganisms across many generations, and these are called prions. The [*PSI*+] prion of yeast *Saccharomyces cerevisiae* represents a heritable amyloid of the eRF3 translation termination factor, also known as the Sup35 protein^6–9^. When Sup35 switches into the prion form, translation termination efficiency decreases. Thus, the [*PSI*+] prion is an epigenetic element with a loss-of-function phenotype^10^.

The Sup35 protein is composed of three domains. The N-terminal domain (N, residues 2-123) is primarily responsible for the prion properties and is rich in glutamine and asparagine (QN). The middle domain (M, residues 124-253) is rich in glutamic acid and lysine, and it includes the major target for Ssa1/2 chaperones (residues 143-164), which is critical for prion propagation^11^. The C-terminal domain (C, residues 254-685) performs an essential function in translation termination.

Many amyloids exhibit structural polymorphism – i.e., the ability of the same amyloid-forming protein to acquire many different cross-β structures. Different amyloid polymorphs of α-synuclein, tau protein, Aβ peptide, PrP and other human proteins were shown to be associated with different types of respective neurodegenerative disorders^12–17^. Although many such pathology-related amyloid structures are now established^18,19^, the mechanisms relating these structures to pathologic phenotypes remain poorly understood due to the high complexity of multicellular organisms.

In contrast, baker’s yeast is a simple unicellular organism, where the phenotypic manifestations of prions and their relation to prion structures can be observed and studied more easily^10,20^. The [*PSI*+] prion phenotype is usually detected using nonsense mutations in adenine biosynthesis pathway genes, which cause accumulation of the red intermediate of adenine biosynthesis. The Sup35 prion state reduces the efficiency of translation termination, allowing partial readthrough of mutant nonsense codons and reverting colony color from red to some shades of pink or white, depending on the extent of Sup35 inactivation (nonsense suppressor phenotype).

The [*PSI*+] prion also has a high number of structural variants differing by the level of nonsense suppression that are usually roughly divided into “strong” and “weak” types based on colony color phenotype^21–23^. This criterion is not sufficiently strict, but we have shown that strong and weak variants represent two distinct classes that can be reliably distinguished by their proteinase K (PK) digestion patterns and opposite reaction to overproduction of *SUP35* and *HSP104* genes^24^. The most systematic study of the [*PSI*+] prion variation was able to distinguish 23 variants^25^. However, only five of them, including one strong and four weak variants, were obtained in a standard way, through transient Sup35 overproduction, while the other 18 variants were generated by prion passage on various Sup35 mutants poorly compatible with the original [*PSI*+]. Thus, most of the studied [*PSI*+] likely belong to the former category of five “standard” variants. Our structural probing with PK of 19 strong and 12 weak [*PSI*+] prion isolates allows us to assume that more than one strong [*PSI*+] variant may exist, but their structures are basically similar, though they may differ in minor details^24,26^. Weak [*PSI*+] variants should also have something common in their structures, but their structural variation is higher than within the strong class.

It is widely assumed that many, if not all, phenotypic features distinguishing strong and weak variants result from the more frequent fragmentation of strong Sup35 prions, compared to weak ones, by the Hsp104/Ssa1/Sis1 chaperone mechanism in yeast^27–29^. If so, the study of the structure-to-phenotype relation in the case of [*PSI*+] prion variants could be reduced to a significant extent to seeking the mechanism relating the structures of Sup35 prions and the frequency of their fragmentation in yeast.

Current understanding of the properties of Sup35 prions is largely based on studies of amyloid fibrils that spontaneously form in vitro by the Sup35NM protein lacking the C domain. The fibrils formed at 4 °C produce mainly strong [*PSI*+] prion upon transfection into yeast cells, while those formed at 37 °C produce mainly weak [*PSI*+]^30,31^. Due to this, it is commonly assumed that the former fibrils (Sc4) are structurally equivalent to strong prion variants, while the latter (Sc37) are equivalent to weak ones. Deuterium exchange and proteinase K (PK) digestion data indicate that the Sup35 amyloid cores include amino acid residues 2-46 in Sc4 and 2-72 in Sc37^32,33^. The Sc4 fibrils also showed significantly higher fragility than Sc37^31^. This allowed thinking that the higher fragility of strong prion variants defines their easier and more frequent fragmentation compared to weak ones. An alternative idea was that Hsp104 is sufficiently powerful to fragment any prion, but the critical point is the ability of the chaperone machinery to recognize a prion as a target, and the smaller prion core of strong prions leaves longer unfolded regions for better chaperone recognition^34^.

However, PK mapping of prion cores of 26 strong and weak Sup35 prions isolated from yeast revealed that they all have a core structure comprising residues 2-72. However, while residues 2-32 are fully protected, the residues 33-72 are protected only partly and significantly better protected in strong variants^24^. This causes a paradox that PK maps of strong [*PSI*+] are highly similar to Sc37 amyloid, rather than to Sc4, while weak [*PSI*+] is similar to Sc4^24,33^.

Current work, together with that of Tanaka et al.^35^, resolves this paradox by showing the substantial structural difference between the Sup35 amyloids obtained in vitro and prion polymers propagating in yeast cells. Using the cryo-EM approach together with biochemical, computational, and genetic methods, we established the 3D-structure of the N-terminal core of strong-type Sup35 amyloid fibrils, analyzed the impact of different parts of this core on the overall stability of these fibrils, and the effect of different mutations earlier reported to have anti-prion action. Based on these findings, we propose a model explaining how different Sup35 structures predetermine the different frequencies of fibril fragmentation in vivo and, eventually, the different severity of the [*PSI*+] prion phenotype.

## Results

### [*PSI*+]-S7 and [*PSI*+]-W2 prion fibrils differ in fragmentation due to their physical properties, rather than chaperone binding

**Fig. 1:**
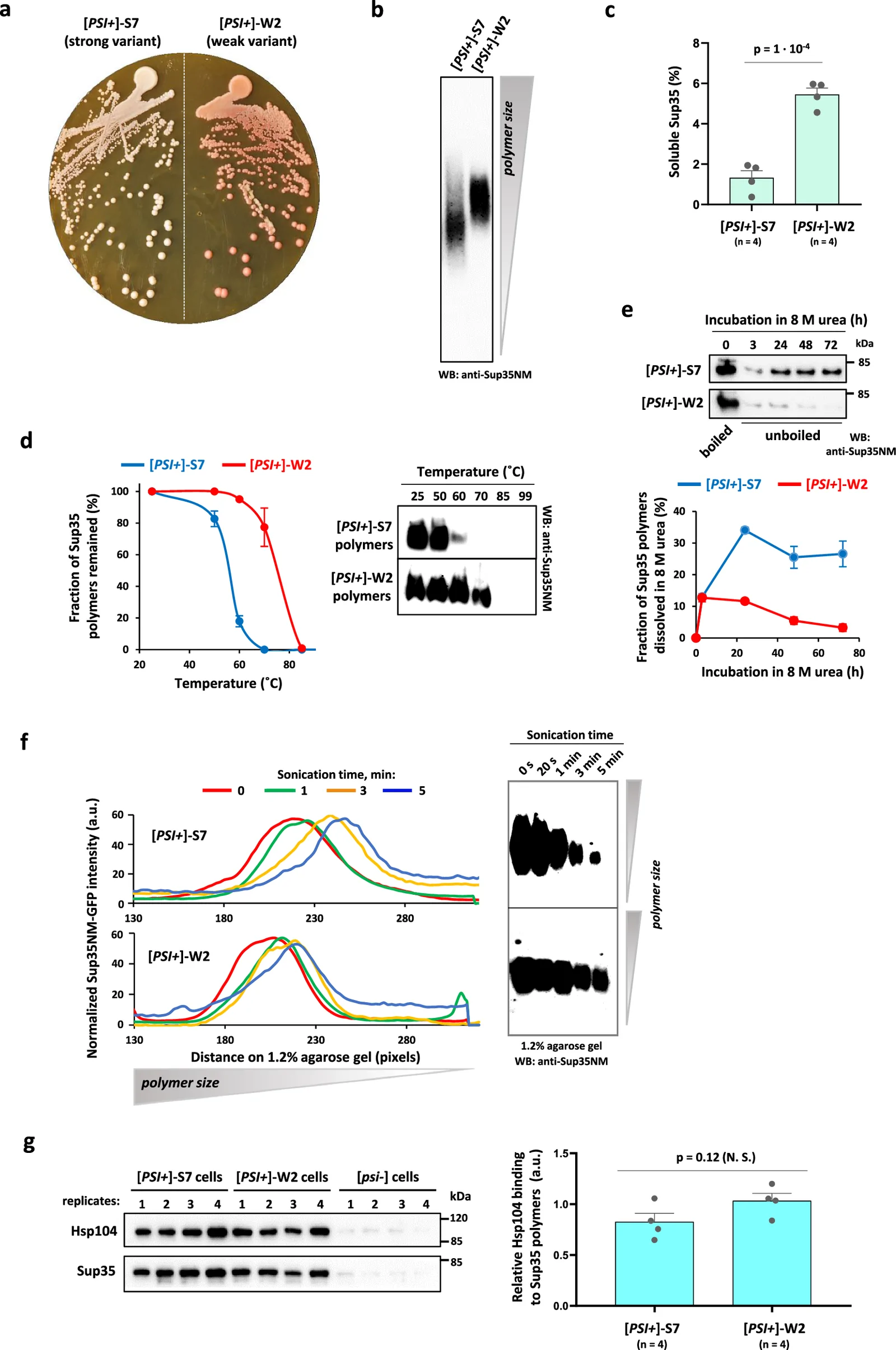
Properties of strong and weak [*PSI*+] prion variants used in this work. **a** Phenotype of the strong and weak [*PSI*+] variants in the *ade1-14* yeast strain. **b** Comparison of the Sup35 polymer size by the SDD-AGE and immunoblotting. **c** Comparison of the proportion of soluble Sup35 in the cells carrying strong and weak [*PSI*+] variants, determined as a proportion of Sup35 molecules capable of entering a 10% polyacrylamide gel without boiling. Data represent mean ± SEM (*n* = 4 biological replicates). Comparison of resistance to heat (thermal stability assay, **d**), urea (**e**) and mechanical shearing via ultrasonication (**f**) for [*PSI*+]-S7 and [*PSI* + ]-W2 prion fibrils. In **d**, the fractions of Sup35 prion fibrils, which resisted the indicated temperatures, were determined by SDD-AGE. Data represent mean ± SEM (*n* = 3 biological replicates). In **e** purified Sup35NM-GFP fibrils were incubated for the indicated time in 8 M urea, and the proportion of released monomeric Sup35NM-GFP was determined by SDS-PAGE, using a sample boiled without urea as a measure of the total Sup35NM-GFP amount in fibrils. Data represent mean ± SEM (*n* = 3 biological replicates). In **f** equal aliquots of Sup35NM-GFP fibrils were ultrasonicated for different durations, and the change in fibril size distribution was assessed by SDD-AGE. Experiment was repeated two times with similar results. In all cases, electrophoresis was followed by immunoblotting and densitometry of blots. **g** Hsp104 binding to strong- and weak-type Sup35 prion aggregates assessed via their co-sedimentation from cell lysates. The relative quantity of Hsp104 and aggregated Sup35 in pellets was assessed by Western Blotting, using [*psi*-] cells as a negative control. Data represent mean ± SEM (*n* = 4 biological replicates). *P*-values were calculated using two-tailed Student’s *t*-test.

To study the structural basis of the difference between strong and weak [*PSI*+] variants, we took [*PSI*+]-S7 and [*PSI*+]-W2 as typical representatives of the “strong” and “weak” classes as defined in our previous work^24^. Consistent with earlier observations made by our and other groups on strong and weak [*PSI*+] variants^22,36,37^, the cells bearing the strong [*PSI*+]-S7 variant had a smaller size of Sup35 polymers, a lower proportion of Sup35 monomers, and a higher number of propagons (Fig. 1a–c, and Supplementary Fig. 1a) compared to the weak [*PSI*+]-W2 variant. Both variants were mitotically stable under normal growth conditions, but [*PSI*+]-W2 inheritance was significantly destabilized following short-term heat shock (Supplementary Fig. 1b).

We compared the mechanical properties of Sup35 prions from [*PSI*+]-S7 and [*PSI*+]-W2 cells and their interaction with chaperones. The [*PSI*+]-S7 prion was less resistant to heating and high-molar urea (Fig. 1d, e, and Supplementary Fig. 1c), and more fragile (Fig. 1f), consistent with earlier observations of ex vivo strong and weak [*PSI*+] polymers^34,38^ and their presumed in vitro assembled counterparts Sc4 and Sc37^30,33^. Assaying the amyloid/chaperone co-sedimentation, we observed that Sup35 fibrils of [*PSI*+]-S7 and [*PSI*+]-W2 variants bind similar amounts of the Hsp104 chaperone (Fig. 1g), the key player in fragmentation and disaggregation of Sup35 polymers^27,29^. This agrees with the finding that strong- and weak-type Sup35 prion polymers bind equal amounts of the Ssa1/2 chaperones^39^. These results indicate that strong- and weak-type polymers represent equally attractive substrates for the chaperones performing amyloid fragmentation. Summing up, our data suggest that the fibrils' stability is the primary feature that determines the frequency of their fragmentation, the balance between soluble and amyloid Sup35 protein and, eventually, the “strength” of the [*PSI*+] prion phenotype.

### The role of Core1A and Core1B in maintaining the stability of Sup35 fibrils

In different [*PSI*+] variants prion fibrils may have up to four PK-resistant cores located within the region 2-221, but only the N-terminal core (Core1) is present in all studied preparations and defines the strong or weak prion phenotype^24^. According to the PK data, Core1 spans residues 2-72 in strong and most weak variants and can be divided into two subdomains: the fully PK-resistant Core1A (residues 2-32) and the partially resistant Core1B (residues 33-72).

Because the observed difference in physical properties of strong- and weak-type prion fibrils in vivo cannot be explained by the Core1 length (which is identical), it is likely caused by the difference in the cross-β packing of the same region. Paradoxically, the strong-type Sup35 prion fibrils, which are less stable than weak-type ones (Fig. 1d–f), and more fragmentable in vivo (Fig. 1b), always have a more PK-resistant Core1B than weak-type ones^24^. This discrepancy suggests that in strong-type prion fibrils, Core1B is more tightly associated with Core1A than in weak variants and plays a more important role in the stabilization of the whole prion structure. Higher stability of weak fibrils is probably related to the more robust Core1A. To test this hypothesis, we analyzed the effects on the [*PSI*+]-S7 and [*PSI*+]-W2 prions of several uncharged-to-charged prion-destabilizing amino acid substitutions in the Core1A and Core1B areas using plasmid shuffle.

**Fig. 2:**
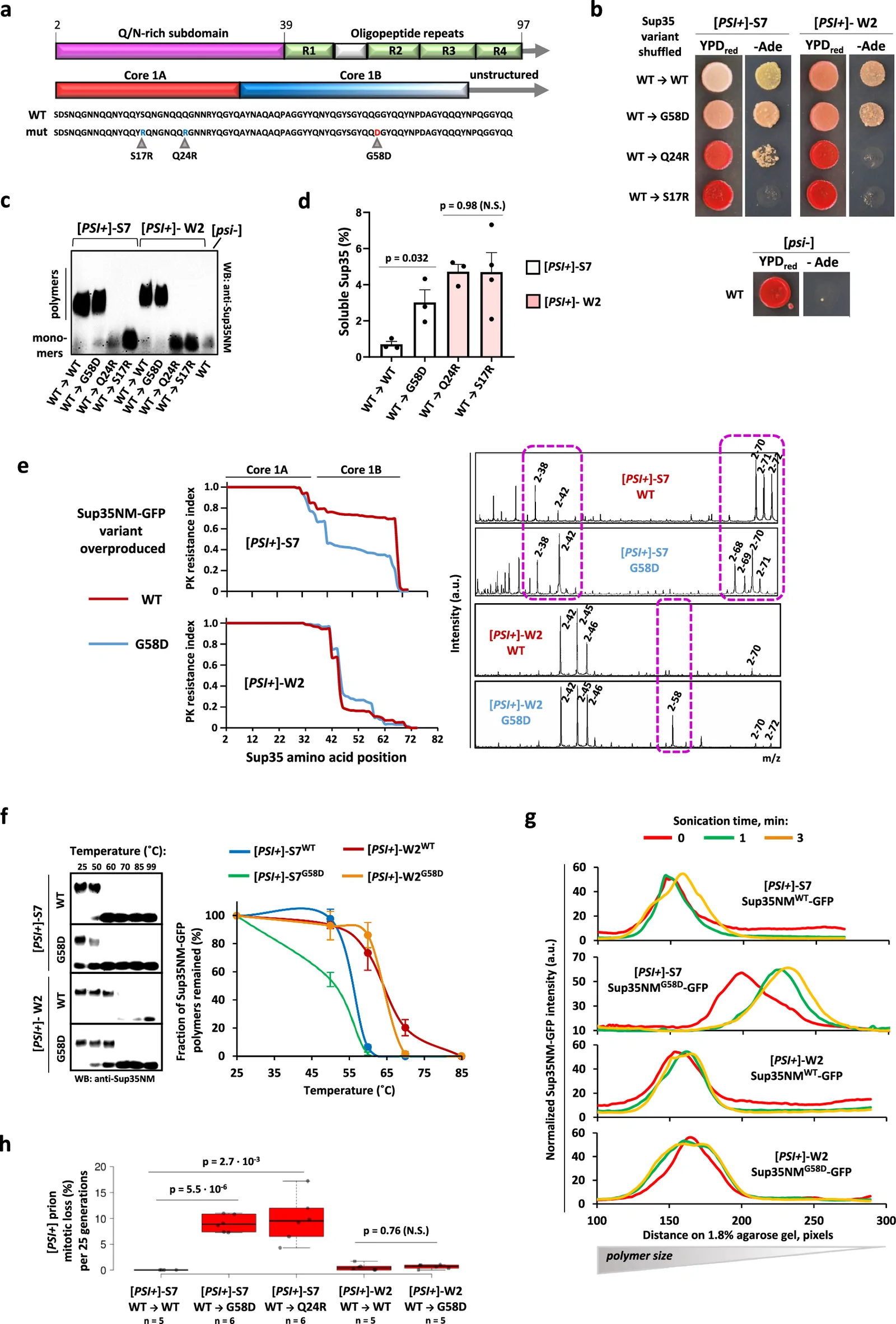
Dissecting the roles of Core1A and Core1B in the stability and phenotype of strong and weak [*PSI*+] variants. **a** Schematic representation of the Sup35 N-domain showing its Q/N-rich and oligopeptide repeats (R1-R4) regions, location of the fully PK-resistant Core1A and partly PK-resistant Core1B, and the studied anti-prion mutations. **b** Nonsense-suppressor and *Ade+* phenotypes of [*PSI*+]-S7 and [*PSI*+]-W2 cells with the *SUP35*^*WT*^ gene shuffled for either *SUP35*^*G58D*^, *SUP35*^*Q24R*^ or *SUP35*^*S17R*^ mutant alleles. **c** SDS-resistant Sup35 polymers detected by SDD-AGE in the lysates of [*PSI*+]-S7 and [*PSI*+]-W2 cells where *SUP35*^*WT*^ was shuffled for the indicated mutant variants. **d** Proportions of soluble Sup35 and Sup35^G58D^ in [*PSI*+]-S7 and [*PSI*+]-W2 cells, estimated by the Sup35 ability to enter a 10% polyacrylamide gel without boiling. Data represent mean ± SEM (*n* = 3 biological replicates). **e** Mapping of PK-resistant cores of prion fibrils isolated from [*PSI*+]-S7 and [*PSI*+]-W2 cells and composed of either Sup35NM^WT^-GFP or Sup35NM^G58D^-GFP proteins. Comparison of the resistance to heat (thermal stability assay, **f**) and mechanical shearing by ultrasonication (**g**) for strong- and weak-type prion polymers consisting of either Sup35NM^WT^-GFP or Sup35NM^G58D^-GFP proteins. Data represent mean ± SEM (*n* = 3 biological replicates). **h** Comparison of prion mitotic stability for [*PSI*+]-S7 and [*PSI*+]-W2 cells with the *SUP35*^*WT*^ allele replaced for the *SUP35*^*G58D*^ or *SUP35*^*Q24R*^ alleles. Center lines show the medians; box limits indicate the 25th and 75th percentiles as determined by the R software; whiskers extend 1.5 times the interquartile range from the 25th and 75th percentiles. *P*-values were calculated using two-tailed Student’s *t*-test.

The G58D substitution, located in the Core1B area (Fig. 2a), was previously shown to cure only strong [*PSI*+] variants^40,41^. Therefore, we anticipated that this mutation could destabilize either Core1B itself or its association with Core1A. Indeed, in our hands, *SUP35*^*G58D*^ allele, even as a sole source of Sup35 protein in cells, did not completely cure the [*PSI*+]-S7 prion, but noticeably altered its properties: weakened its nonsense-suppressor phenotype (Fig. 2b, and Supplementary Fig. 2a), increased proportion of soluble Sup35 (Fig. 2d), and decreased mitotic stability (Fig. 2h). The PK resistance of the Core1B was noticeably reduced (Fig. 2e). These features may seem to indicate a shift in the [*PSI*+]-S7^G58D^ properties towards a weak prion variant. However, contrary to such interpretation, [*PSI*+]-S7^G58D^ polymers showed lower thermal stability and higher fragility than [*PSI*+]-S7^WT^ (Fig. 2f,g) - and, therefore, their manifestations in vivo likely originated from the increased (rather than decreased, as in the case of “true” weak [*PSI*+]) fragmentation by chaperones (Supplementary Fig. 3c), leading to a partial amyloid disaggregation, in agreement with the earlier assumption of Pei et al.^42^. These data support the hypothesis that the Core1B region is important for the in vivo stability of strong [*PSI*+]. Reverse shuffle (Sup35^G58D^→ Sup35^WT^) led to restoration of the initial [*PSI*+]-S7 phenotype (Supplementary Fig. 2d,e), suggesting that Core1B has recovered its initial fold that was likely dictated by Core1A.

In contrast to the strong [*PSI*+]-S7 prion, weak [*PSI*+]-W2 variant was nearly unaffected by the Sup35^WT^→ Sup35^G58D^ substitution, as follows from its PK resistance profile, nonsense suppressor phenotype, and physical properties of fibrils (Fig. 2b–h, and Supplementary Fig. 2a). This suggests that Core1B is more loosely packed in the [*PSI*+]-W2 fibrils and is less important for structure stabilization and fibril’s physical properties. Earlier, we observed a weak [*PSI*+]-C3 isolate that lacked protease resistance after the residue Y45^43^, but its thermal stability was even higher (Supplementary Fig. 3a,b) than that of [*PSI*+]-W2. This allows us to conclude that the region 46-72, a major part of the Core1B, is unlikely to have a significant impact on the overall stability of weak-type fibrils.

Anti-prion substitutions S17R and Q24R^44^ are located in the Core1A area (Fig. 2a). The Sup35^WT^→ Sup35^S17R^ shuffle cured both [*PSI*+]-S7 and [*PSI*+]-W2 prions (Fig. 2b). The Sup35^WT^→ Sup35^Q24R^ substitution also cured [*PSI*+]-W2 variant, while [*PSI*+]-S7 prion has acquired an unusual very weak nonsense-suppressor phenotype (dark-pink colony color, Fig. 2b) with the absence of SDS-resistant polymers (Fig. 2c). Since this phenotype did not emerge in the [*psi-*] cells Sup35^WT^→ Sup35^Q24R^ shuffle (Supplementary Fig. 2b), and was partially sensitive to Hsp104 inhibition (Supplementary Fig. 2c), we suggest that it likely is of prion nature, but its prion structure is substantially altered and further study of its properties is beyond the scope of this article.

Therefore, we conclude that Core1A is essential for the maintenance of both strong and weak prions variants, while Core1B is less important for weak variants and highly important for strong ones as a stabilizer of strong-type prion polymers. Based on these conclusions, we suggest that Core1A itself has a less robust and stable fold in the strong-type fibrils compared to weak-type ones and, therefore, requires stabilizing interactions with Core1B.

### Cryo-EM analysis of the [*PSI*+]-S7 prion structure

Next, we sought to resolve the atomic structures of ex vivo Sup35NM-GFP fibrils purified from either [*PSI*+]-S7 or [*PSI*+]-W2 cells. The cryo-EM reconstruction was hindered by the structural noise caused by the GFP globule and Sup35 M domain, and these parts were enzymatically removed from the purified prion. However, this caused precipitation of prion fibrils. The issue was solved by the introduction of a negatively charged D/E-rich cluster after residue G96 (Supplementary Fig. 5a). For both variants, most of the fibrils consisted of a single protofilament of ~6 nm width. For [*PSI*+]-S7 fibrils, we observed alteration of nearly untwisted (~54%) and clearly twisted (~46%) segments on the same filaments (Supplementary Figs. 6a and 7a,b). These segments displayed similar 2D class features apart from the degree of twist, indicating a common molecular fold (Supplementary Fig. 7d). [*PSI*+]-W2 fibrils had an almost untwisted morphology (Supplementary Fig. 7c), which made them unsuitable for further structure reconstruction. 2D class averages of the untwisted segments of [*PSI*+]-S7 and [*PSI*+]-W2 fibrils exhibited different patterns of longitudinal striations, indicating different cross-β packing of these prion variants (Supplementary Fig. 7a–c).

**Fig. 3:**
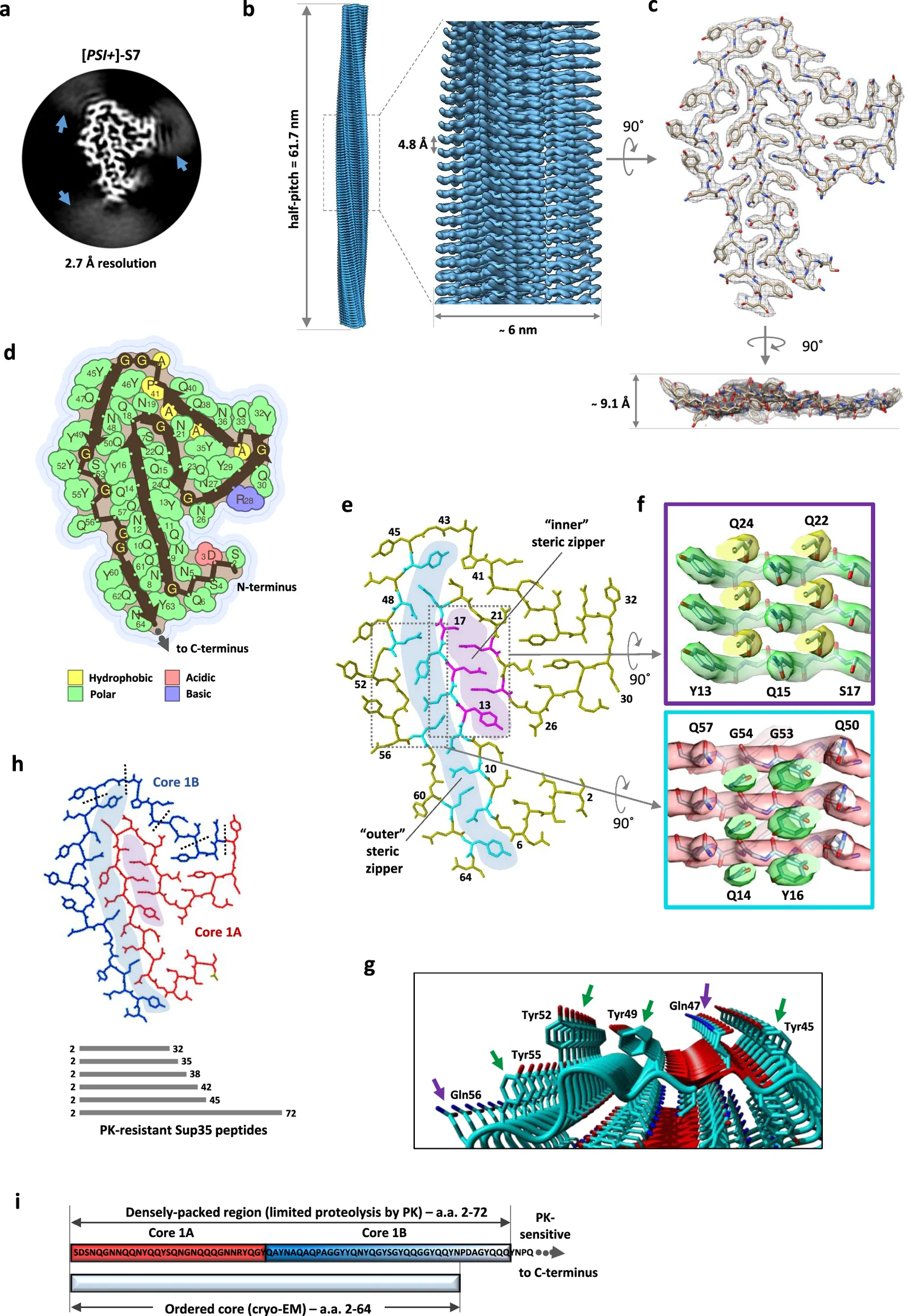
Cryo-EM structure of the [*PSI*+]-S7 prion fibril core. **a** Cross-sectional view of the 3D reconstruction of [*PSI*+]-S7 amyloid core structure. Rendered 3D model of the ordered part of [*PSI*+]-S7 amyloid fibril (**b**), and the atomic model of a single protomer superimposed on the sharpened cryo-EM density map (**c**). **d** Topology of the [*PSI*+]-S7 amyloid fold, showing the location of β-strands. The sidechains are color-coded based on their physiochemical properties. **e** Atomic model of the [*PSI*+]-S7 amyloid core showing the “inner” and “outer” steric zipper elements. **f** Close-up of the parts of “inner” and “outer” steric zippers showing their “staggered” organization along the *Z*-axis of the fibril. **g** Close-up of several π-columns formed by tyrosine residues (green arrows) and amide ladders formed by glutamine residues (violet arrows) stabilizing the core structure along the axis of [*PSI*+]-S7 prion fibrils. **h**, **i** Comparison of the PK-protected region and cryo-EM-solved ordered structure of Core1. Dotted lines indicate the main stop sites for proteinase K digestion of Core1B. The “inner” and “outer” steric zipper zones are highlighted in magenta and blue, respectively. The major PK-resistant peptides observed in the [*PSI*+]-S7 preparation are shown below.

Using the clearly twisted segments, we resolved the [*PSI*+]-S7 fibril core structure at 2.7 Å resolution (Fig. 3a–d, and Supplementary Fig. 9a–d). The corresponding helical parameters were determined as a rise of 4.78 Å and a twist of –1.39°, corresponding to a left-handed pitch of 123.5 nm. A single protomer exhibits a non-planar, undulating arrangement (with an axial span of ~9.1 Å along the fibril axis) and forms an “amyloid key”-like fold^18^ in the S2-N64 region. This span of the ordered structure is generally consistent with the length of [*PSI*+]-S7 Core1 as determined by PK mapping (Fig. 3i). Residues 2–4 precede the structured region and are partially stabilized. Among them, residue 4 is sufficiently constrained for its position to be assigned unambiguously, whereas residues 2 and 3 remain partially flexible, contributing diffuse density without discernible side-chain features.

The Sup35 protomer molecule is stabilized by numerous “horizontal” interactions (Fig. 3e,f) between amino acid side chains, which stabilize the structure in the plane perpendicular to the fibril axis. An “inner” zipper, formed by a short β-arch spanning Y13–G25, stabilizes the central Q/N-rich subdomain of the core (Core1A). An “outer” zipper is generated by the antiparallel packing of N8–Q18 against Q47–Y63, thereby linking Core1A to the peripheral subdomain (Core1B) and integrating both into a single ordered fold (Fig. 3h). Because the protomer shape is non-planar, these interfaces are volumetric rather than flat: side chains interdigitate with a slight axial offset, producing three-dimensional packing that reduces solvent access and increases shape complementarity (Fig. 3f). In both zippers, the opposing β-strands are staggered by approximately half of the helical rise, a feature typical of amyloid steric zippers that promotes volumetric packing. Quantitative analysis of interface geometry shows that the inner and outer steric zipper interfaces both display high shape complementarity (SC)^45^ values of 0.81–0.82, in contrast to the lower value (0.76) displayed by the interfaces located outside of the zipper-forming regions (Supplementary Fig. 9g). Notably, the SC values observed for the [*PSI*+]-S7 steric zippers approach those reported for the microcrystals formed by the classical Sup35-derived peptide GNNQQNY (SC = 0.86), where the highly complementary polar side chains form completely dry steric zipper interfaces^46^. The order of the corresponding sidechains packing is also similar between the GNNQQNY microcrystal and the outer zipper of [*PSI*+]-S7 amyloid (Supplementary Fig. 9h).

Along the fibril axis, stabilization is provided by both a canonical in-register cross-β hydrogen-bonding network between backbone amides and carbonyls, as well as by some “vertical” interactions of the side chains: twelve tyrosine residues form intermolecular π-columns, while numerous asparagine and glutamine residues form amide ladders (Fig. 3g). A more detailed description of fibril-stabilizing interactions and some additional structural features is provided in the *Supplementary Results* section. Notably, the major PK-resistant peptides from [*PSI*+]-S7 digestion start from the N-terminus (S2 residue) and end at the positions Y32, Y35, Q38, A42 and Y45^24^, which belong to the region between the “inner” and “outer” steric zippers (Fig. 2h). As the core structure predominantly consists of polar residues (Fig. 3d), its surface is largely hydrophilic and uncharged (Supplementary Fig. 9e,f).

### Thermodynamic profiling of [*PSI*+]-S7 atomic structure and its interaction with the anti-prion point mutations in the Sup35 N-domain

**Fig. 4:**
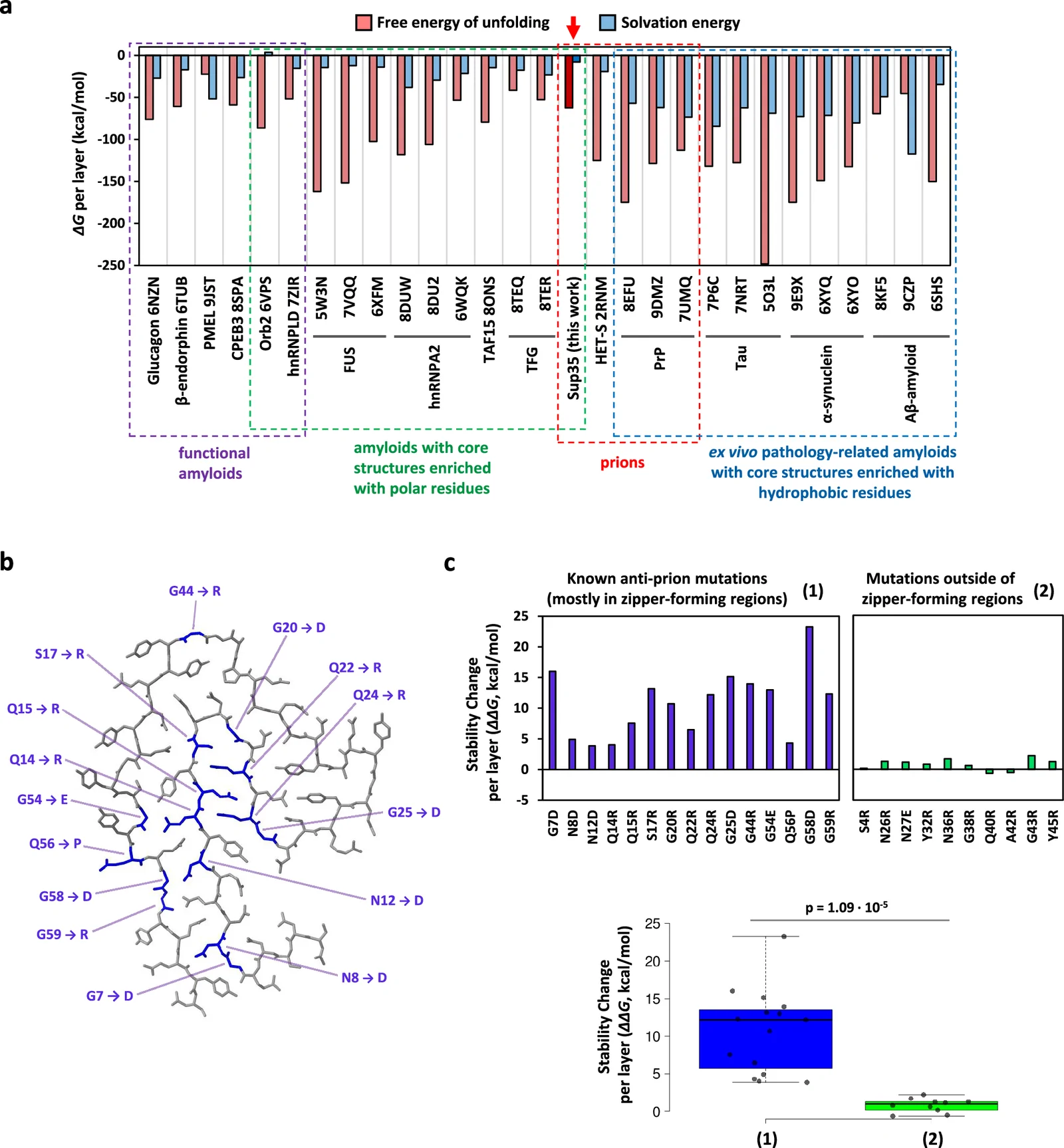
Thermodynamic profiling of the [*PSI*+]-S7 core structure and the effects of anti-prion mutations. **a** Gibbs free energy of unfolding and solvation energy of Sup35/[*PSI*+]-S7 prion structure (red arrow) in comparison with that of several representatives of different amyloid classes. **b** Amyloid core structure with known anti-prion mutations^44,54,55^. Most of them represent uncharged-to-charged residues substitutions in the zipper-forming regions. **c** Predicted change in the free energy of unfolding (ΔΔG) calculated by FoldX software for the anti-prion Sup35 mutations in comparison with the wild type structure. In contrast to known anti-prion mutations, manually selected substitutions (uncharged-to-charged residues) located outside of the zipper-forming regions have only a minor impact on fibril stability. The bar plot shows the values calculated for each individual mutation. The box plot shows the grouped values for the “anti-prion” and “control” mutation groups. Center lines show the medians; box limits indicate the 25th and 75th percentiles as determined by R software; whiskers extend 1.5 times the interquartile range from the 25th and 75th percentiles. *P*-value was calculated using two-tailed Student’s *t*-test.

To make a structure-based assessment of the [*PSI*+]-S7 fibrils stability, we estimated their free energy of unfolding (Δ*G*_Unf_) using the FoldX algorithm^47^. For comparison, we also calculated the solvation energy (Δ*G*_Solv_), which has been used to estimate the amyloid structure's stability in many published works^48–52^. To get a broader picture, we also calculated Δ*G*_Unf_ and Δ*G*_Solv_ for a set of published amyloid structures belonging to different partially overlapping biological and physicochemical groups. We found that [*PSI*+]-S7 structure has Δ*G*_Unf_ comparable to that of some structures of known functional amyloids, and higher (i.e., less favorable) than that of pathology-related amyloids (Fig. 4a). In contrast, its Δ*G*_Solv_ is very high (i.e., non-favorable) even in comparison with the majority of functional amyloids. Notably, the only known amyloid structure with even higher Δ*G*_Solv_ compared with Sup35 fibrils is the Orb2 functional amyloid of *D. melanogaster*^53^. Given that both of these structures are dominated by stabilizing steric zipper motifs (as in the case with many other highly stable pathological amyloid structures), we suggest that the Δ*G*_Solv_ parameter is less accurate for assessing amyloid structure stability than the Δ*G*_Unf_ parameter, and seems to greatly underestimate the true stability of amyloid structures consisting mostly of polar residues (Supplementary Fig. 10).

We further used the resolved [*PSI*+]-S7 amyloid core structure to provide structural rationale for the effect of known anti-prion mutations in the Sup35 N-terminal domain. To this end, we used a set of 15 such mutations that mostly cause uncharged-to-charged residue changes^44,54,55^. A majority of these substitutions are located in the areas of either “inner” or “outer” steric zippers (Fig. 4b), suggesting that the zipper regions are most important for the structure stability. Using FoldX, we estimated the fibril stability change upon these mutations, and found all substitutions to be significantly destabilizing (i.e., increasing Δ*G*_Unf_; Fig. 4c). Remarkably, by testing another 10 manually selected uncharged-to-charged residue substitutions located in non-zipper-forming regions, we found their effect on [*PSI*+]-S7 structure stabilization energy to be negligible. Thus, [*PSI*+]-S7 structural analysis indicates which structural motifs are most sensitive to substitutions. One could also suggest that introducing charged residues into the tightly locked zippers would loosen their packing, thereby decreasing fibril stability.

### Comparison of ex vivo Sup35 prion structure with in vitro formed Sup35 amyloids

**Fig. 5:**
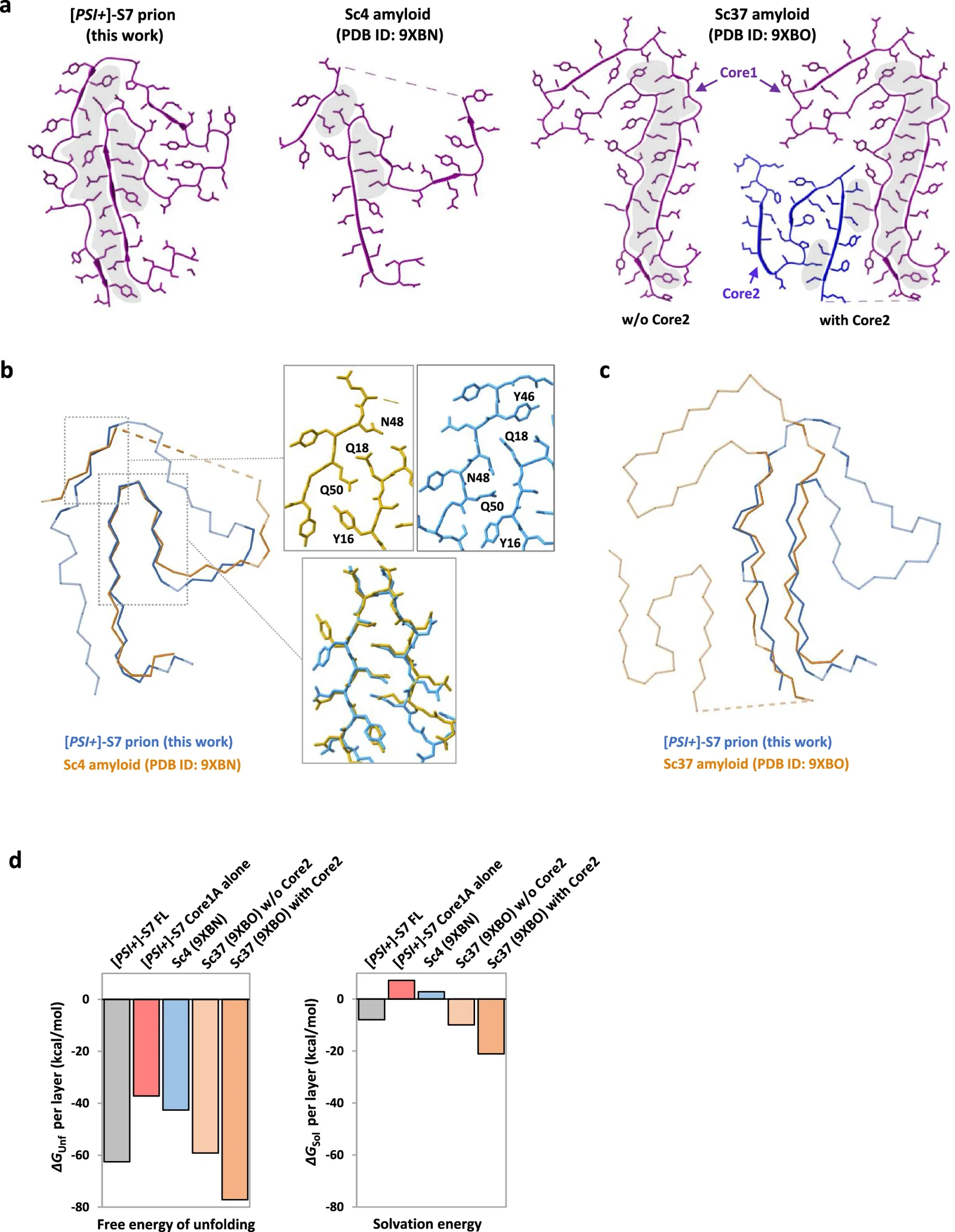
Comparison of ex vivo Sup35 prion structure with in vitro formed Sup35 amyloids. **a** Structure of [*PSI*+]-S7 ex vivo prion fibrils in comparison with the Sc4 (PDB ID: 9XBN) and Sc37 (PDB ID: 9XBO) amyloids formed in vitro. Steric zipper elements are highlighted in grey. Structural alignment of [*PSI*+]-S7 with Sc4 (**b**) and Sc37 (**c**) structures, with the close-up of some regions fully or partially similar between [*PSI*+]-S7 with Sc4 structures. **d** Comparison of the free energy of unfolding (*ΔG*_Unf_) and solvation energy (*ΔG*_Solv_) between [*PSI*+]-S7, Sc4 and Sc37 structures.

The work of Tanaka et al., published recently^35^, describes the cryo-EM structures of Sc4 and Sc37 amyloids that are presumed to be structural equivalents of the strong and weak Sup35 prions, respectively. Comparison of the structures of Sc4 amyloid with that of the strong [*PSI*+]-S7 prion reveals both a significant similarity and a substantial difference (Fig. 5a,b). The similarity relates to region 2-26, which we consider to be the most significant and defining part of the [*PSI*+]-S7 structure. The highest similarity is observed in the area of Y13–G25 β-arch (Fig. 5b, lower panel). A β-strand N27-Q30 is also found in both structures, but it is oriented differently. In contrast to [*PSI*+]-S7, in the Sc4 structure, the major part of the N36-N64 stretch (roughly corresponding to the Core1B area) is fully or partially disordered. The exception is the Q47-S53 stretch, which forms a short β-strand attached to the top of Y13-G25 hairpin by the zipper-like interactions. However, the register of these interactions in [*PSI*+]-S7 and Sc4 structures is different (Fig. 5b, upper panels).

Surprisingly, the structure of Sc37 amyloid also bears noticeable similarity to that of [*PSI*+]-S7 prion: both structures contain a long zipper-like element between the antiparallel stretches Q6-Q23 and Y45-N63 (Fig. 5a,c), and a similar overall span of the Core1 (residues 5-65 and 2-64, respectively). The thermodynamic parameters (Δ*G*_Unf_ and Δ*G*_Solv_) are also similar between [*PSI*+]-S7 prion and Sc37 amyloid structures, as well as between Sc4 and the Core1A part of [*PSI*+]-S7 structure (Fig. 5d). Tanaka et al. also reported that about half of the Sc37 amyloid segments also contained the additional core (K102-Q132); this subtype of Sc37 structure has Δ*G*_Unf_ ~ 25% lower compared with that of [*PSI*+]-S7 structure. Of note, the 2D class features of Sc37 amyloid differed from those of [*PSI*+]-W2 prion, which indicates a substantial structural difference between these fibril types (Supplementary Fig. 7e).

## Discussion

### The mechanism of prion fragmentation

Previously, two hypotheses were proposed explaining what defines the difference in the frequency of fragmentation between the weak and strong Sup35 prions. One hypothesis proposed that the more frequent fragmentation of the strong prion is due to its lower mechanical strength. The other assumed that since Hsp104 is a powerful molecular machine with 12 ATPase units, the limiting factor is not its ability to unfold a prion, but its ability to find its target, which occurs with the help of Hsp70 (Ssa1/2) and Hsp40 (Sis1). In support of the latter model, it was observed that progressive deletions of the oligopeptide repeats in the Sup35 N domain, which were assumed to be chaperone targets, reduced prion fragmentation, thus increasing prion size and eventually causing prion loss^56,57^. However, the set of data, presented in this and some other works^34,38,39^, supports the mechanical strength being the key factor. This also agrees with the recent finding that the Sup35 interaction with chaperones mainly occurs through the Ssa1/2 binding site located beyond the prion-forming region, at Sup35 residues 143-164^11^. While one can note that the mechanical strength model was generally more popular than its alternative, it was based predominantly on the assumption of the equivalence of synthetic Sup35 amyloids and in vivo Sup35 prions, which proves to be incorrect according to this study.

### Cryo-EM structure of Sup35 prion

Many human amyloid fibrils are composed of two or more protofilaments. In contrast, the Sup35 polymers extracted from [*PSI*+]-S7 and [*PSI*+]-W2 cells consisted of single filaments. This rules out the scenario where the higher stability of weak [*PSI*+]-W2 filaments is related to their double- or triple-protofilament arrangement. In vitro, Sup35 usually forms single filaments, though twin filaments were also observed^35^. However, it appears likely that only single-filament fibrils could succeed as viable prions in yeast. Propagation of yeast prions relies on the ability of Hsp104 to fragment prion fibers^58^. While in single-filament fibers this requires the extraction of just one protomer, in multi-protofilament fibers several protomers opposing each other should be extracted, which should significantly reduce the probability and resulting frequency of fragmentation. Thus, the requirement for frequent fragmentation of yeast prions may preclude the appearance of multi-filament yeast prions, in contrast to human amyloids, which do not depend on frequent fragmentation.

Here we found that [*PSI*+]-S7 prion has the parallel-in-register architecture. This confirms the earlier observations of Wickner and coauthors^59–61^, and disproves the alternative solenoid-type model^62,63^. Of note, the solenoid model was formulated based on a study of in vitro generated Sup35 fibrils^62^, for which the parallel in-register architecture was also shown recently^35^. Importantly, PK digestion patterns of strong and weak prions are similar within these groups^24^, suggesting that many (if not all) in vivo [*PSI*+] variants are likely to share similar strong or weak fibril architecture.

**Fig. 6:**
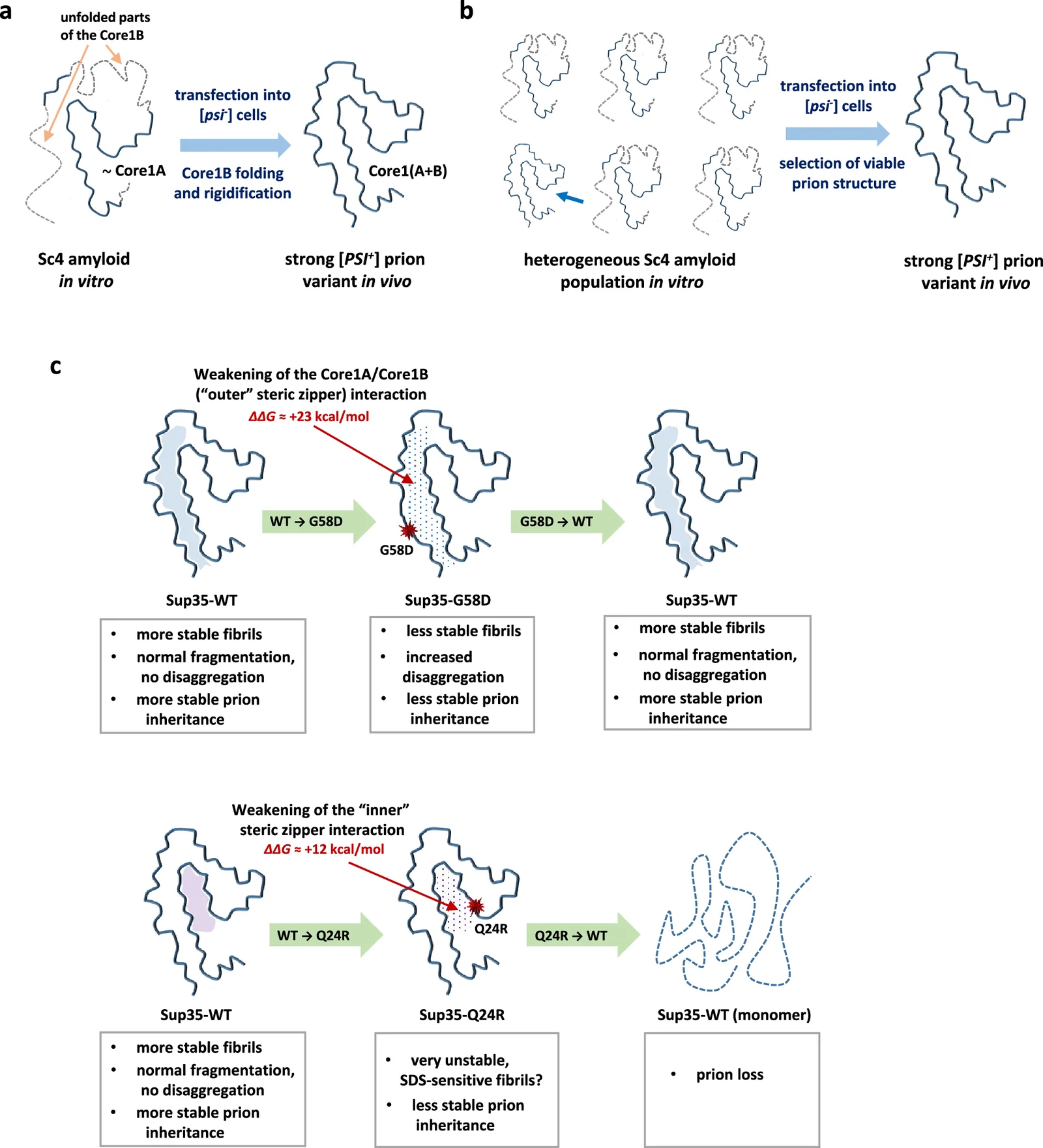
Possible mechanisms of conversion of Sc4 amyloid into a strong prion in vivo, and of the effect of anti-prion substitutions on the [*PSI*+]-S7 prion structure. **a**, **b** Two ways of conversion of in vitro formed Sc4 amyloid into a strong prion propagating in vivo. **a** Structural rearrangement of Sc4 amyloid by extension of the existing cross-β fold to the N36-N64 region of Sup35, and **b** positive selection in yeast cells of rare most fitted Sc4 polymorphs. **c** Proposed mechanism of the anti-prion action of the G58D and Q24R substitutions with respect to the strong [*PSI*+]-S7 prion structure.

Our data indicate that the Core1A is the key element of the strong and weak prion structures. While some known anti-prion substitutions are located in the Core1B region^54^, mutagenesis of the whole *SUP35* N domain yielded the anti-prion substitutions located only between residues 8 and 24^44^. This region belongs to the Core1A and to the inner steric zipper (Figs. 3e and 4b). The data from this study and previous publications show that substitutions within Core1A generally are significantly more efficient in curing [*PSI*+] than mutations in the Core1B^38,44,54^. This emphasizes the central role of Core1A and the supplementary role of Core1B. Restoration of the initial [*PSI*+]-S7 phenotype (i.e., strong nonsense-suppression and high mitotic stability) after the reverse *SUP35*^*G58D*^ → *SUP35*^*WT*^ shuffle (Fig. 6c, and Supplementary Fig. 2d,e) also suggests that the Core1A region determines the folding of the rest of the structure. While the dominance of the Core1A appears to be largely related to its physical strength, our recent study shows that the terminal location itself is a significant factor helping any sequence to influence the folding of the remaining part of the prion structure. The most telling observation was that a random 23-residue sequence added to the Sup35 N-terminus and produced in [*PSI*+] cells acquired a prion fold and altered the fold and overall span of the Sup35 strong and weak prion structures at their original location^64^.

### Relation between the in vitro formed Sup35 amyloids and the prion propagating in vivo

Earlier works revealed a significant difference in the PK maps of the strong and weak Sup35 prions with their presumed counterparts generated in vitro^24,33^. This work, together with Tanaka et al.^35^ resolves this discrepancy by establishing cryo-EM structures and showing that the PK maps correspond well to these structures, while the structures differ significantly.

The Sc4 and [*PSI*+]-S7 structures are very similar in their most important fully PK-resistant part, or Core1A, but have little in common for the rest of the structures. Yeast transformation with Sc4 results in a strong [*PSI*+] variant identified as VH^25^. Our studies of ex vivo prions from 19 strong [*PSI*+] isolates reveal that all of them, including VH and [*PSI*+]-S7, show a high level of PK protection in the region 33-72 in contrast to Sc4 amyloid, which yields no PK-resistant peptides larger than 2-46^24,33^. Thus, the conversion of Sc4 amyloid into a yeast prion can be accompanied by a significant change in its structure. There are two ways how the Sc4 fold can turn into a strong prion fold in yeast: either through rearrangement of the region 32-64 in the Sc4 main fraction (Fig. 6a) or through selection of a minor [*PSI*+]-S7-like conformer present in the Sc4 preparation (Fig. 6b). The latter opportunity appears less likely, since such [*PSI*+]-S7-like conformer appears to be at least very rare in Sc4 preparations according to PK digestion spectra and the cryo-EM data^33,35^. Interestingly, a recent study using in vivo NMR indicates that both such processes occur with in vitro-formed α-synuclein amyloid: upon transfection into cells, the proportion of major and minor isoforms reversed, and the disordered flanking regions of the fibrils were remodeled^65^.

Our observations with the Sup35 G58D anti-prion mutation suggest why the [*PSI*+]-S7 fold may have selective preference in yeast cells over Sc4. This mutation destabilizes the steric zipper which attaches Core1B to the Core1A, making the whole prion structure less stable (Figs. 2e–g and 4b,c), which should increase prion disaggregation and promote prion loss^42^. Apparently, the Sc4 structure, which almost lacks this steric zipper, should be even less stable than the strong prion with G58D substitution and thus unable to propagate in yeast.

Although the cryo-EM structure of the weak [*PSI*+] variant has not been solved, some important notes about the relation of the Sc37 structure and the natural weak [*PSI*+] prions can be made. Comparison of the Sc37 and [*PSI*+]-S7 structures explains why their PK digestion patterns are so similar. Both structures include similar, though not identical, steric zippers formed by stretches N8-Y16 and Q50-Y63, which ensure high resistance to PK of the region Y45-Q72 in both cases. However, in all studied weak [*PSI*+] prions, and [*PSI*+]-W2 in particular, this region shows low PK resistance^24^.

It was shown that Sc37 transfection into yeast produces a weak [*PSI*+] variant similar to the VK variant^25^, which exhibits a PK digestion pattern typical of all weak variants^24^, but different from that of Sc37^33^. Thus, the Sc37 fold changes in yeast, but it is difficult to conclude whether this occurs through rearrangement of the Sc37 major fraction or through in vivo selection of a minor fraction.

The difference between the structures formed in vitro and propagating in vivo could be related to the action of the Hsp104 chaperone, whose prion-fragmenting action is required for prion propagation. Too soft amyloids are likely to be disassembled in vivo, while too rigid ones may be unable to propagate due to insufficiently frequent fragmentation (Supplementary Fig. 3c). In support of such a role of Hsp104, the Sup35 prion variants propagating in the presence of weakened Hsp104^T160M^ cannot propagate in the presence of wild-type Hsp104 and vice versa^38^.

### Relation between the span of Sup35 amyloid core structures and PK-resistant peptides

Mapping of amyloid cores through partial proteinase K digestion is an affordable supplement and, in some senses, an alternative to the full-atomic structure reconstruction. Now, PK maps are available for several ex vivo yeast prions^64,66^, but their full-atomic structures are missing. Thus, it is of interest to understand how PK maps relate to actual structures. This work and those of the Tanaka group^33,35^ provide a rare opportunity to learn this.

The ordered core of [*PSI*+]-S7 fibrils defined by the cryo-EM (residues 2-64) is smaller than the PK-resistant region (residues 2-72). A similar difference was observed for the Sc37 amyloid (5-65 versus 2-72)^33,35^. Sc4 has an ordered core comprising residues 4-35 and 47-52, while the PK-resistant region includes residues 2-46; the cryo-EM structure of S17R4 amyloid includes residues 88-129 versus the PK-resistant core at residues 81-148; the S17R37 cryo-EM structure includes residues 69-132 and the PK-resistant core at residues 62-144. Thus, PK-protected regions are about 7-12 residues longer at each end, excluding the N-terminus, which has no such length of unstructured sequence. The existence of such a gap could be explained by the fact that the PK active site is located within a cavity in the PK molecule^67^, and to be digested at its end, an unfolded region of certain length needs to enter this site and to reach the catalytic center. Still, it remains unclear how PK can cut within the structured region at residues 32 to 64, and this mechanism requires further investigation.

According to these observations, the borders of the Core1A and Core1B could be corrected. Core1A may include residues 2-24, and Core1B – residues 25-64. In this case, the border of Core1A would coincide with the end of the inner steric zipper in the [*PSI*+]-S7 structure. However, it should be kept in mind that placing the border at residues 24/25 is fairly arbitrary. Actually, the division of Core1 into two parts is only to indicate that these parts differ in their PK resistance, properties and roles.

## Methods

### Plasmid construction

To create the pRS315-SUP35 and pRS316-SUP35 plasmids, the XhoI-XbaI chromosomal fragment containing the *SUP35* gene was inserted into pRS315 and pRS316 plasmids^68^ by XhoI and XbaI sites. To introduce the G58D, S17R or Q24R mutations, the whole plasmid pRS315-SUP35 was amplified with the G58D, S17R or Q24R pairs of primers (see Supplementary Table 1). The PCR fragments were treated with the ligase-independent ClonExpress II One Step Cloning Kit (Vazyme, Nanjing, China) and used to transform *Escherichiacoli*. To create the pRS315-SUP35C plasmid, the XhoI-XbaI fragment of the pRG416-3ATG plasmid^69^ was ligated to the XhoI and XbaI sites of pRS315. This fragment encodes the essential Sup35C domain but lacks the region encoding N and M domains. To create the pYES2-SUP35DE-GFP plasmid, a *SUP35* fragment was amplified with Pxd22 and Sup35DE-Rf primers (Supplementary Table 1), and the resulting PCR fragment was inserted into the pYES2-SUP35NM-GFP plasmid^24^ cut with BstXI and MlsI (BalI), using ClonExpress II One Step Cloning Kit. This inserted an acidic stretch DDDNEDSEEDDED followed by trypsin and thrombin cutting site followed by SUP35 codon 155.

### Yeast strains and cultivation conditions

The work used derivatives of the 74-D694 yeast strain (*MATa, ade1-14, trp1-289, his3Δ−200, ura3-52, leu2-3,112*) carrying either [*PSI*+]-S7 or [*PSI*+]-W2 prion. To prevent the de novo formation of any alternative amyloid conformers in the experiments with Sup35NM-GFP overproduction, the *RNQ1* gene in these strains was disrupted by *HIS3* insertion. For the plasmid shuffle experiments, derivatives of [*PSI*+]-S7 and [*PSI*+]-W2 strains were created, where the chromosomal *SUP35* gene was disrupted by *TRP1* insertion and the *SUP35* gene was introduced on the centromeric pRS316-SUP35 vector. The standard yeast media were used. Synthetic defined media (SD) contained 6.7 g/L yeast nitrogen base, 20 g/L glucose or 24 g/L galactose, and required amino acids. For colony color development, SD contained a reduced amount of adenine (7 mg/L, or 1/3 of the standard). For overproduction of proteins of interest under control of the *GAL1* promoter, we used SD-Gal medium with 24 g/L galactose instead of glucose and 100 mg/L of adenine. Rich YPD medium contained 10 g/L yeast extract, 20 g/L peptone, 20 g/L glucose and 20 g/L agar when required. For better development of the colony color phenotype, solid YPD-red medium was used (6 g/L yeast extract (Oxoid, UK), 20 g/L peptone, 20 g/L glucose and 20 g/L agar). Cells were grown at 30 °C.

### Yeast genetic procedures

Yeast transformation with DNA was performed using the standard LiAc-PEG method^70^. Yeast transfection with prion fibrils (“spheroplast transformation”) was performed as described by Tanaka et al.^30^, using an “empty” pRS316 vector as a selection marker, and the 74-D694 [*psi-*] yeast strain as a recipient.

To switch the Sup35^WT^ allele to Sup35^MUT^, we used the plasmid shuffle technology. Cells where the genomic *SUP35* was deleted and the episomal Sup35^WT^ was encoded on the centromeric pRS316 (*URA3*) plasmid were transformed with pRS315 (*LEU2*) plasmid encoding the Sup35^MUT^ allele. These *SUP35* genes included the native promoter. Transformants were grown on the medium lacking leucine for 3 days, and then streaked on the synthetic medium containing 1 mg/ml of fluoroorotic acid (FOA) to ensure loss of the *URA3* plasmid. After 3-day growth, the cells from fully grown colonies were pooled, streaked to single cells on the YPD-red medium, grown for 3 days, and the loss of the plasmid encoding the Sup35^WT^ allele was confirmed by the inability to grow on the synthetic medium lacking uracil. To make a reverse shuffle, cells carrying pRS315- Sup35^MUT^ plasmid were transformed with the pRS316-Sup35^WT^ plasmid and grown on the medium lacking uracil for 3 days. The transformants were streaked to single cells on the same medium, grown for 3 days, and the individual colonies lacking pRS315-Sup35^MUT^ plasmid were found by their inability to grow on the medium lacking leucine.

To make yeast spots, cells were grown overnight in liquid YPD, then cell suspensions were diluted to OD600 = 1, and 8 μl of suspensions were spotted onto the YPD-red plates. For better development of colony color, spots were grown at 30 °C for 3 days and then incubated at 4 °C for 1 day prior to imaging.

The propagon number in [*PSI*+] cells was evaluated as described by Cox et al.^37^. Briefly, cells were streaked on YPD plates, grown at 30 °C for 3 days, freshly grown colonies were streaked on YPD plates with 4 mM GuHCl and grown for 1.5–2 days. Small colonies were collected and streaked on the SD medium containing 0.4 mg/ml adenine (2% of standard concentration). At this adenine concentration, only the cells that inherited [*PSI*+] prion can form fully grown colonies. Plates were incubated at 30 °C for 5 days, and the number of fully grown colonies corresponding to each colony from the YPD-GuHCl plate was calculated. It is assumed that this value corresponds to the number of propagons (the elementary units of prion inheritance) in a single yeast cell carrying [*PSI*+] prion.

To estimate the level of prion stability upon mitotic cell growth, several [*PSI*+] colonies were pooled, streaked to single cells on a YPD plate and grown at 30 °C for 3 days. Then 5 individual colonies (as individual biological replicates) were separately streaked to single cells on YPD-red plates, grown at 30 °C for 3 days, then incubated for 1 day at 4 °C, and the proportion of red (i.e., [*psi*-]) colonies was determined for each replicate. As the single cell takes ~25 generations to form a fully grown colony, the obtained values correspond to the percentage of prion loss after 25 generations of mitotic growth. To demonstrate the increased level of the weak [*PSI*+]-W2 prion loss upon the short-term heat shock, cells carrying [*PSI*+]-S7 and [*PSI*+]-W2 prions were grown in liquid YPD medium to the log phase (OD600 ~ 1.5), then incubated at 45 °C for 30 min, streaked to single cells on YPD-red plates, and grown at 30 °C for 3 days. The proportions of red and red/pink sectored colonies were calculated.

To demonstrate that overproduced Sup35DE-GFP protein can propagate the [*PSI*+]-S7 prion upon cell division, we transformed [*PSI*+]-S7 cells with pYES2-SUP35DE-GFP plasmid (*GAL1* promoter) for overproduction of prion protein, and the rescue plasmid pRS315-SUP35C to reduce the overproduction-driven cellular toxicity and allow cell division. The transformants were grown to the stationary phase on selective SD-Glucose medium, diluted 1000-fold with selective SD-Galactose medium, and then grown to the stationary phase (~3 days), thus undergoing ~10 divisions on the medium, inducing Sup35DE-GFP overproduction. After this, cell suspension was streaked to single cells on YPD-red plate, grown for 3 days, and the color phenotypes of resulting colonies were compared to the initial [*PSI*+]-S7 color phenotype.

### Purification of Sup35NM-GFP prion fibrils from yeast for cryo-EM analysis

#### Method consideration and creation of the protein construct

In [*PSI*+] cells with the native production level of endogenous Sup35 protein, prions exist as a population of short fibrils^36^, which are not suitable for the cryo-EM structure reconstruction. To overcome this issue and facilitate prion purification, we overproduced the GFP-tagged construct containing the prionogenic part of Sup35 protein (residues 1-239) using the multicopy pYES2 plasmid (Thermo Fisher Scientific) carrying the *SUP35NM-GFP* gene under the control of *GAL1* promoter. Upon this procedure, this construct adopts the pre-existing prion fold^24^ and forms long filaments sequestered into large higher-order aggregates^71^.

In this and previous work^72^, we observed that Sup35NM-GFP fibrils per se are poorly suitable for cryo-EM reconstruction due to the structural noise imposed by GFP globules and the disordered Sup35 M domain (a. a. 125-239). These parts were removed by trypsin. However, the obtained fibrils were prone to precipitation, so only a small proportion of them were separated on cryo-EM grids and suitable for structure reconstruction. To cause repulsion of fibrils, we introduced an acidic cluster DDDNEDSEEDDEDGGP**R**GSR between G96 and A155 residues, resulting in the Sup35DE-GFP protein (Supplementary Fig. 5a). Using Matrix-assisted laser desorption/ionization (MALDI) spectrometry, we confirmed that trypsin cleaves this protein at arginine residue (in bold), but does not cleave at the only upstream R28 located inside the prion core.

#### Purification of Sup35DE-GFP fibrils

Yeast cells carrying either [*PSI*+]-S7 or [*PSI*+]-W2 prions were simultaneously transformed with two plasmids: pYES2-SUP35DE-GFP for overproduction of prion protein, and the rescue plasmid pRS315-SUP35C, which reduced the cellular toxicity associated with Sup35 overproduction in the [*PSI*+] cells, thereby increasing the level of Sup35DE-GFP production. Cells were grown to the stationary phase in selective SD-glucose medium, and then a twofold volume of selective SD-Gal medium was added. Cells were incubated for 15 h and harvested. As a validation of Sup35DE-GFP coaggregation with pre-existing [*PSI*+] prion polymers, we monitored that all Sup35DE-GFP protein produced by cells is sequestered into the fluorescent foci (Supplementary Fig. 4c) using the fluorescent microscope Axioskop 40 (Zeiss, Germany) and the agar pad technique^73^. Cell pellets (2*4 ml in 50 ml tubes) were mixed with an equal volume of glass beads and half a volume of lysis buffer (TBS (Tris Buffered Saline) buffer, 5 mM PMSF, 1 mM DTT). Cells were broken by vortexing at maximum speed for 10 min at 4 °C. Cell lysates were transferred to 15-ml tubes and spun down at 2500 × *g* for 15 min at 4 °C. The supernatant lacking GFP and was discarded, and the upper layer of the pellet containing GFP was resuspended in 4 ml of fresh TBS buffer supplemented with 100 µg/ml RNaseA, 50 µg/ml DNase I, placed on top of sucrose gradient #1 (TBS based, 0.5 ml of 60% sucrose and 3 ml of 30% sucrose in 15 ml Falcon tube), and spun for 15 min at 2500 × *g*, 4 °C. The GFP-containing fraction of the gradient was collected, and sucrose was diluted twofold to reduce sucrose concentration.

To disassemble higher-order clumps of Sup35DE-GFP prion polymers into individual fibrils, SDS was added to a final concentration of 4%. Samples were incubated for 20 min at 10 °C to avoid both protein degradation and SDS precipitation. The dissolution of visible Sup35DE-GFP clumps into individual fibrils was monitored (Supplementary Fig. 4d) with the fluorescent microscope Axioskop 40 (Zeiss, Germany). Then, samples were centrifuged for 1 min at 21,300 × *g* to remove the remaining debris, and GFP-containing supernatant was placed on top of sucrose gradient #2 (based on TBS buffer with 0.3% Sarcosyl: 100 µl of 70% sucrose + 200 µl of 60% sucrose + 700 µl of 30% sucrose in 3.5 ml tube) and spun down in Beckman Coulter Optima Max ultracentrifuge SW50 rotor at 260,000 × *g* for 4 h at 10 °C. GFP-tagged prion fibrils were trapped in the 60% sucrose fraction. The GFP-positive fraction of the gradient was collected, frozen in liquid nitrogen and stored at −70 °C.

To remove GFP and “fuzzy coat” part of Sup35DE-GFP fibrils, the samples were diluted twofold with TBS buffer and treated with 0.1 mg/ml trypsin (Sigma) for 60 min at 37 °C. To terminate reaction, 1 mM PMSF was added after 1 h incubation. To remove the unwanted digestion products, the preparations were centrifuged at 260,000 × *g* for 4 h at 4 °C using sucrose gradient #2. Pure fibrils were found in the 60% sucrose fraction. This fraction was dialyzed against 1 L of 0.1X TBS buffer with 0.1% Sarcosyl. The samples were then concentrated ~10-fold by evaporation of water excess for 1 h at room temperature. The scheme of fibril isolation, trypsin treatment and purification is shown in the Supplementary Fig. 4a,b. The purity of the final sample, as determined by the SDS-PAGE, was ~95% (Supplementary Fig. 4e). The Sup35DE fibrils showed reasonably small aggregation on the cryo-EM grids, suitable for cryo-EM data collection.

To ensure that prion strain properties were not changed upon Sup35DE-GFP overproduction and protein purification procedure, we transfected the 74-D694 [*psi*-] cells with either [*PSI*+]-S7 or [*PSI*+]-W2 fibrils, extracted and treated as described above. In both cases, the initial prion phenotype (either strong or weak nonsense suppression) was reproduced (Supplementary Fig. 5b). A more detailed analysis of the relevance of the obtained [*PSI*+]-S7 structure to the native [*PSI*+]-S7 prion is presented in the Supplementary Notes.

### Cryo-electron microscopy

#### Sample preparation and data acquisition

Quantifoil R1.2/1.3 Cu grids were glow-discharged for 20 s at 20 mA (0.26 mbar) using a PELCO easiGlow system. 3 μl of the fibril suspension (0.38 mg/ml) were applied to the grids, blotted for 3 s at 100% humidity and 4 °C, and plunge-frozen in liquid ethane using a Vitrobot Mark IV (Thermo Fisher Scientific, USA). The grid was then stored in liquid nitrogen until use. Cryo-EM data were collected on a Titan Krios 60-300 transmission electron microscope (Thermo Fisher Scientific, USA) equipped with a field emission electron gun X-FEG (Thermo Fisher Scientific, USA), spherical-aberration corrector (CEOS GmbH, Germany), a post-column BioQuantum energy filter (Gatan, USA) and a K3 direct electron detector (Gatan, USA) in counting mode using SerialEM 4.055 and 9-hole (2 exposures per hole) image-shift data acquisition strategy at the National Research Centre “Kurchatov Institute”. The microscope was operated at 300 kV with a nominal magnification of 81,000×, corresponding to a pixel size of 0.863 Å at the specimen level, and an electron energy selecting slit of 20 eV. A total dose of 52 e⁻/Å² within a 3.5 s exposure time was fractionated into 70 frames, resulting in an electron dose of 0.74 e⁻/Å² per frame. A total of 9015 movies were collected in a nominal defocus range from −0.7 to −2.0 μm with a step of 0.1 μm. Detailed parameters of data acquisition are listed in Supplementary Table 2.

#### Image processing

Movies were motion-corrected and CTF parameters were estimated in Warp. Micrograph power spectra showed a characteristic ~4.8 Å meridional reflection, indicative of well-ordered cross-β structure. Filaments were automatically picked with crYOLO using an inter-box step of 24 Å (five helical units per step).

Helical segments were extracted with box sizes of 1024 and 512 pixels (binned to 256 and 128 for the initial steps) to support accurate crossover measurements and efficient 2D classification, respectively. 1024-pixel and 512-pixel particles were used for initial 2D classification, helical pitch estimation, and ab initio model generation. The 2D classes from 1024-pixel boxes reported a crossover distance of ~70 nm. After initial 2D classification with 512-pixel boxes, false positives and broken filaments were removed and the dataset was partitioned into twisted (585,256 segments) and low-twist (674,012 segments) subsets. For homogeneous processing, only the twisted subset was retained. An initial 3D model of the twisted fibrils was then generated using *relion_helix_inimodel2d*, assuming a left-handed helix, and used as input for subsequent refinement.

Starting from 585,256 segments extracted with a 384-pixel box, an initial helical refinement in CryoSPARC yielded a reconstruction at 3.6 Å, which improved to 3.23 Å after local refinement with a soft mask around the fibril core. This was followed by two consecutive rounds of 3D classification without alignment in CryoSPARC, each combined with cycles of CTF refinement, helical refinement, and local refinement. In the first round, a well-defined subset comprising 163,538 segments produced a 2.81 Å reconstruction (helical parameters: rise 4.78 Å, twist –1.278°). A second round of classification of this subset was performed to further purge residual heterogeneity, yielding 50,658 high-quality segments. Refinement of this final subset in CryoSPARC resulted in a global resolution of 2.69 Å, as determined by the gold-standard FSC 0.143 criterion. The best-defined map corresponded to fibrils exhibiting a rise of 4.782 Å and a twist of –1.388°. The map was sharpened with a negative B-factor of –58 Å².

#### Model building and refinement

A single protomer was built de novo into the sharpened map in Coot, with unambiguous sequence assignment from residue 4 to residue 64. Density preceding residue 4 was poorly resolved: residues 2–3 were therefore modeled according to sequence context (initiator Met1 absent; Ser2 Nα-acetylated; Asp3 present), while acknowledging their flexibility in the map. The built protomer was stacked into a five-layer segment by rigid-body placement in UCSF Chimera and subsequently subjected to PHENIX real-space refinement with NCS constraints between the five chains and individual ADPs. Model-to-map and stereochemical validation statistics are summarized in Supplementary Table 2. Shape complementarity (SC) values were calculated using the *sc* utility from the CCP4 suite for selected pairs of residue segments forming lateral β-sheet interfaces, with molecular surfaces constructed from five neighboring fibril layers to reduce edge effects.

The graphic representation of the cryo-EM data processing and structure reconstruction pipeline is presented in the Supplementary Fig. 8.

#### Validation of helical handedness

A left-handed twist was initially assumed based on the prevalence of left-handed helicity in amyloid filaments formed by different proteins and validated using the high local resolution in the ordered core, where main-chain carbonyl oxygen atoms are resolved as distinct density protrusions and fit perfectly into their corresponding densities (Supplementary Fig. 6b). Experimental 2D class averages matched back-projections from atomic models (Supplementary Fig. 7d), confirming that the atomic models capture the morphology of both fibril types. Back-projections were generated from multi-layer fibril models built by applying the appropriate symmetry operations (rotation and translation per layer for the twisted fibril; translation only for the untwisted fibril) to a single-layer atomic model. Density maps were calculated in Chimera, low-pass filtered to 4.5 Å using Relion, and then used to generate projections.

### PK resistance analysis of Sup35NM-GFP prion fibrils

Yeast cells carrying either [*PSI*+]-S7 or [*PSI*+]-W2 prions were transformed with the pYES2-SUP35NM-GFP plasmid (where the Sup35NM sequence was either WT or containing G58D substitution) and grown to the stationary phase in the selective SD-glucose medium. Then a twofold volume of selective SD-Gal medium was added, cells were incubated for 15 h and harvested.

To map the PK-resistant regions of Sup35NM-GFP fibrils, we extracted and purified the Sarcosyl-resistant fractions from large pellets of yeast cells (~3–5  g per preparation), as described in the original work^24^. Briefly, yeast cells were disrupted with glass beads in 50 ml Falcon tubes and the cell debris containing Sup35NM-GFP aggregates was precipitated at 8000 × *g* for 10 min. Fluorescent material was collected from the top of the pellet, mixed with Sarkosyl (final concentration = 5%), sonicated, loaded onto a 20–65% sucrose gradient, and ultracentrifuged at 260,000 × *g* for 4 h at 4 °C. The fluorescent Sup35NM-GFP fraction was collected near the bottom of the gradient. This fraction was diluted to 200 μg/ml and digested with PK (25 μg/ml) for 1 h at RT. As shown previously, PK has no sequence specificity except for not cutting before or after proline^24,64^. The PK-resistant part of the fibrils was then precipitated by adding acetone to 40%, resuspended in water, boiled, and analyzed by MALDI-TOF/TOF mass spectrometer UltrafleXtreme (Bruker, Germany). The spectra were taken in both linear and reflection modes in the mass range of 1 to 10 kDa. Peptides were identified by tandem mass spectrometry (MS-MS) and/or as groups of related peaks in the reflection spectra using Bruker flexAnalysis 3.3 software, assisted with Gpmaw4.04 for calculation of peptide masses. As we have previously shown^24^, the N-terminal methionine of Sup35PD-GFP was completely removed and replaced by an acetyl group in the yeast cells, and thus all N-terminal peptides started from amino acid residue № 2. The PK resistance was calculated based on peak areas of peptides in the linear mode and plotted as a function (*R*_*n*_) of the normalized PK resistance index for a given sequence position (*n*), given by:

1 $${{{\bf{R}}}}_{{{\bf{n}}}}=\sum {{{\boldsymbol{S}}}}_{{{\boldsymbol{n}}}}/\sum {{{\boldsymbol{S}}}}_{{{\boldsymbol{max }}}}$$

where the **∑** ***S***_***n***_ is a sum of the areas of all MS peaks containing this amino acid position (n), and **∑** ***S***_***max***_ – the maximum peak area sum value.

The spectra of [*PSI*+]-S7 and [*PSI*+]-W2 shown in Fig. 2e were reproduced twice for independent preparations of these prions. The spectra presented in Supplementary Fig. 5e were obtained once.

### SDD-AGE analysis of amyloid polymers

Yeast cells were grown in 20 ml of the YPD media to the mid-log phase, collected to 2.4 ml Eppendorf tubes and lysed by vigorous vortexing with glass beads and 100 μl of TBS buffer supplemented with 5 mM PMSF, 1 mM DTT and Complete protease inhibitor cocktail (Roche) at 4 °C for 10 min. Cell lysates were mixed with 4X SDD-AGE sample buffer (1X is 0.5X Tris acetate/EDTA, 2% SDS, 5% glycerol, and 0.05% Bromophenol blue) and subjected to the agarose gel electrophoresis, as described by Kryndushkin et al.^36^. Protein samples resolved in agarose gel were then vacuum-transferred to a nitrocellulose membrane (Porablot, MACHEREY-NAGEL), and subjected to a standard Western-Blotting procedure using anti-Sup35NM rabbit polyclonal primary antibodies.

### Thermal stability assay

The procedure was performed as standard SDD-AGE, but with some modifications^34^. Cells were grown and lysed as described above. To avoid the possible effect of cell lysate components on the thermal stability, the Sup35 aggregates were purified by centrifugation at 20,000 rpm for 30 min at 4 °C. The supernatant was discarded, and the pellet was dissolved at room temperature in 300 μl of SDD-AGE sample buffer. Remaining cell debris was spun down at 4000 rpm for 1 min, and 50 μl sample aliquots were transferred to PCR tubes and incubated at either 25, 50, 60, 70, 85, or 99 °C for 8 min, then cooled to 10 °C and loaded onto a 1.8% agarose gel (gel strength = 1000 g/sm^2^) with 0.1% SDS and analyzed by SDD-AGE followed by Western blotting with anti-Sup35NM primary polyclonal antibodies. The integrated intensity of Sup35NM-GFP smears, as a measure of the amount of Sup35NM-GFP amyloids that withstood heating, was then quantified using Fiji software. For each amyloid preparation, the experiment was performed in 3-4 replicates, and mean ± SEM values were calculated.

### Mechanical fragmentation of Sup35NM-GFP fibrils

The fibrils of Sup35NM-GFP (with either WT sequence of Sup35NM or containing the G58D substitution) were extracted from the yeast cells using a protocol similar to that used for fibril purification for the cryo-EM study: the big clumps of long fibrils were isolated from the yeast lysates, dissolved into single fibrils with 4% SDS, and the fraction of purified fibrils was then obtained by ultracentrifugation in a sucrose gradient. The obtained fractions were dialyzed overnight against the TBS buffer, obtained samples were diluted 20-fold and divided into 0.5 ml aliquotes. One aliquot was left unsonicated, while others were sonicated (2/2 s on/off cycle, 50% amplitude) using a VibraCell sonicator (Sonics & Material Inc, USA) for indicated times at +4 °C. All samples were then mixed with SDD-AGE sample buffer and subjected to 1.8% agarose gel electrophoresis, followed by Western blotting and immunostaining with anti-Sup35NM primary antibodies. The distributions of Sup35NM-GFP signal on the gel, which reflect the Sup35NM-GFP fibril mobility and the shift in their size upon sonication, were quantified using densitometry analysis with FIJI software.

### Urea denaturation assay

Sup35NM-GFP fibrils, templated in vivo by either [*PSI*+]-S7 or [*PSI*+]-W2 prion variant, were isolated as described in the “PK resistance analysis of Sup35NM-GFP amyloid fibrils” chapter. The final samples were diluted 20-fold in TAE buffer with a designated urea concentration and incubated for 8 h at 37 °C without agitation. After that, the Laemmli sample buffer was added, and samples were subjected to a standard SDS-PAGE procedure followed by Western blotting with immunostaining against Sup35NM. At this stage, only the monomeric Sup35NM-GFP molecules, which were released from the polymers upon their urea-mediated denaturation, are capable of entering the polyacrylamide gel. As a measure of total Sup35NM-GFP amount in polymers, similar samples without urea were boiled for 5 min, leading to a total conversion of polymers into monomers. We used a set of urea concentrations (i.e., 0 M, 4 M, 6 M and 8 M) and found that both [*PSI*+]-S7 and [*PSI*+]-W2 polymers started to dissolve only in 8 M urea solution, and the dissolution was much more prominent in the case of [*PSI*+]-S7 polymers (Supplementary Fig. 1C). Further, we made a time-lapse experiment, where the aliquots of either [*PSI*+]-S7- or [*PSI*+]-W2-templated fibrils were incubated at 37 °C for 3–72 h, and then analyzed as described above. The proportion of polymers dissolved in urea was calculated as a ratio of the amount of monomeric protein released in 8 M urea samples to the amount of monomeric protein released in the boiled sample w/o urea. The experiment was performed in triplicate, and mean ± SEM values were calculated for both types of fibrils at each time point.

### Chaperones binding assay

For this experiment, we used isogenic cells with either [*PSI*+]-S7, [*PSI*+]-W2 or [*psi-*] prion status, grown to a logarithmic phase. Cells were lysed using a standard lysis procedure with glass beads in 100 µl of TAE buffer supplemented with 10 mM PMSF, 1 mM NaN_3_ and 0.01 mg/mL RNAseA. NaN_3_ and RNAseA were added to disrupt large polyribosome complexes, which can include monomeric Sup35 in the [*psi*-] cells, and thus to prevent monomeric Sup35 sedimentation upon centrifugation in the later stages. Lysates were transferred into separate tubes, incubated for 10 min at 15 °C, diluted to 1000 µl by TAE buffer, and spun at 6000 × *g* for 2 min to remove cell debris. 600 µl of supernatants were transferred into separate tubes and centrifuged at 20,000 × *g* for 15 min at 4 °C to precipitate prion aggregates with bound Hsp104. Supernatants were removed, 50 µl of 1X SDS-PAGE loading buffer was added, and the pellets were resuspended by vigorous pipetting. Samples were then boiled for 5 min (to dissolve Sup35 amyloid fibrils into monomers), cooled, centrifuged for 1 min at 20,000 × *g*, and then 5 µl aliquots were subjected to a standard SDS-PAGE procedure followed by immunoblotting. Each sample was immunostained with the primary polyclonal antibodies against either Sup35NM or Hsp104. The relative amounts of both Sup35 and Hsp104 proteins in each sample (in the arbitrary units) were determined by the densitometry of protein bands, and the relative Hsp104 binding to Sup35 prion aggregates was assessed in arbitrary units as:

2 $${{{\bf{B}}}}^{{{\bf{X}}}}=	\left({{{{\bf{N}}}}^{{{\bf{X}}}}}_{\left[{{\boldsymbol{PSI}}}+\right]-{{\bf{V}}}}\left({{\bf{Hsp}}}{{\bf{104}}}\right)-{{{{\bf{N}}}}^{{{\bf{Mean}}}}}_{\left[{{\boldsymbol{psi}}}-\right]}\right)\left.\left({{\bf{Hsp}}}{{\bf{104}}}\right)\right)/ \\ 	 \left({{{{\bf{N}}}}^{{{\bf{X}}}}}_{\left[{{\boldsymbol{PSI}}}+\right]-{{\bf{V}}}}\left({{\bf{Sup}}}{{\bf{35}}}\right)-{{{{\bf{N}}}}^{{{\bf{Mean}}}}}_{\left[{{\boldsymbol{{psi}}}}-\right]}\left({{\bf{Sup}}}{{\bf{35}}}\right)\right)$$

where **B**^**X**^ is a relative Hsp104 binding for a given replicate of given [*PSI*+] variant (“*[PSI*+*]-V*”), **N**^**X**^_**[*PSI*+]-V**_ **(Hsp104)** is a Hsp104 band intensity for a given replicate of given [*PSI*+] variant, **N**^**Mean**^_**[*psi*-]**_ **(Hsp104)** is a mean Hsp104 band intensity for [*psi-*] cells, **N**^**X**^_**[*PSI*+]-V**_ **(Sup35)** is a Sup35 band intensity for a given replicate of given [*PSI*+] variant, and **N**^**Mean**^_**[psi-]**_ **(Sup35)** is a mean Sup35 band intensity for [*psi-*] cells.

The experiment was performed in four biological replicates for each cell type, and mean + SEM values were calculated for [*PSI*+]-S7 and [*PSI*+]-W2 prion variants. Importantly, only barely detectable Hsp104 amounts were found in the pellet of [*psi*-] cells, indicating that almost all Hsp104 molecules detected in the pellets of [*PSI*+] cells sedimented due to the binding to Sup35 prion aggregates.

As a control, we also measured the Hsp104 production levels in [*PSI*+]-S7 and [*PSI*+]-W2 yeast strains (Supplementary Fig. 1d). Cells were lysed with glass beads as described above, cell lysates were normalized by adsorption at 280 nm and subjected to standard SDS-PAGE procedure followed by immunoblotting. Each sample was immunostained with the primary polyclonal antibodies against either Hsp104 or β-tubulin (Thermo Fisher Scientific, PA5-16863) as a housekeeping protein. The relative amounts of Hsp104 (in arbitrary units) were determined by the densitometry of protein bands (the amount of Hsp104 in each sample was normalized to the amount of β-tubulin).

### Structure-based energy calculations

To calculate the free energy of unfolding (ΔG_Unf_), and stability change upon mutations (i.e., the difference in free energy of unfolding between the fibrils formed by the mutated and wild-type Sup35 protein), we used the FoldX 5.1 plugin in the YASARA program.

ΔG_Unf_ was calculated (default temperature = 298 K, default ionic strength = 0.05 M) using the equation:

3 $${{{\boldsymbol{\Delta }}}{{\bf{G}}}}_{{{\bf{Unf}}}}={{{\bf{W}}}}_{{{\bf{vdw}}}}\cdot {{{\boldsymbol{\Delta }}}{{\bf{G}}}}_{{{\bf{vdw}}}}+{{{\bf{W}}}}_{{{\bf{solvH}}}}\cdot {{{\boldsymbol{\Delta }}}{{\bf{G}}}}_{{{\bf{solvH}}}}+{{{\bf{W}}}}_{{{\bf{solvP}}}}\cdot {{{\boldsymbol{\Delta }}}{{\bf{G}}}}_{{{\bf{solvP}}}}+{{{\boldsymbol{\Delta }}}{{\bf{G}}}}_{{{\bf{wb}}}}+\\ {{{\boldsymbol{\Delta }}}{{\bf{G}}}}_{{{\bf{hbond}}}}+{{{\boldsymbol{\Delta }}}{{\bf{G}}}}_{{{\bf{el}}}}+{{{\boldsymbol{\Delta }}}{{\bf{G}}}}_{{{\bf{Kon}}}}+{{{\bf{W}}}}_{{{\bf{mc}}}}\cdot {{\bf{T}}}\cdot {{{\boldsymbol{\Delta }}}{{\bf{S}}}}_{{{\bf{mc}}}}++ {{{\bf{W}}}}_{{{\bf{sc}}}}\cdot {{\bf{T}}}\cdot {{{\boldsymbol{\Delta }}}{{\bf{S}}}}_{{{\bf{sc}}}}$$

where **ΔG**_**vdw**_ is the sum of the van der Waals contributions of all atoms with respect to the same interactions with the solvent; **ΔG**_**solvH**_ and **ΔG**_**solvP**_ are the differences in solvation energy for apolar and polar groups respectively when these change from the unfolded to the folded state; **ΔG**_**hbond**_ is the free energy difference between the formation of an intra-molecular hydrogen bond compared to inter-molecular hydrogen-bond formation (with solvent); **ΔG**_**wb**_ is the extra stabilizing free energy provided by a water molecule making more than one hydrogen bond to the protein (water bridges) that cannot be taken into account with non-explicit solvent approximations; **ΔG**_**el**_ is the electrostatic contribution of charged groups, including the helix dipole; **ΔS**_**mc**_ is the entropy cost of fixing the backbone in the folded state; this term is dependent on the intrinsic tendency of a particular amino acid to adopt certain dihedral angles; **ΔS**_**sc**_ the entropic cost of fixing a side chain in a particular conformation.

For a comparison with the Sup35 amyloid core structure solved in this work, the ΔG_Unf_ values were also calculated for a set of manually selected amyloid structures representing different, partially overlapping physicochemical and biological groups: prions, pathological amyloids, functional amyloids, amyloid structures enriched in polar residues, and amyloid structures enriched in hydrophobic residues. To obtain more consistent results, all types of fibrils were standardized by length (to be 5-mers), and the calculated ΔG_Unf_ values were normalized for a single layer of the fibril.

The solvation energy (ΔG_Solv_) values were calculated using the Amyloid Illustrator package^18,52^ (default pH = 7). As in the case of ΔG_Unf_, the solvation energy was normalized for a single layer and compared with the values calculated for a set of published amyloid structures representing different biological and physicochemical groups.

To calculate the change in ΔG_Unf_ upon mutations (stability change, ΔΔG), we used the model of [*PSI*+]-S7 fibril comprising 5 protomers and introduced the corresponding residue substitution in all of these protomers (using the “Mutate multiple residues” feature in FoldX5.1; *T* = 298 K, ionic strength = 0.05 M, pH = 7, VdW design = 2). ΔΔG was determined as the difference between the ΔG_Unf_ values of mutated and WT molecules. The resulting values were normalized for a single layer of the fibril. The positive values correspond to decreased structure stability, and negative values correspond to increased structure stability.

### Image representation

Image representations of reconstructed densities and atomic models were created with UCSF Chimera^74^, ChimeraX^75^ and the Amyloid Illustrator package^18^. To make an alignment between [*PSI*+]-S7 (this work), Sc4 (PDB ID: 9XBN) and Sc37 (PDB ID: 9XBO) structures, we used the Pairwise Structure Alignment tool on the www.rcsb.org website (TM-align algorithm).

### Reporting summary

Further information on research design is available in the Nature Portfolio Reporting Summary linked to this article.

## Supplementary information

Supplementary Information

Reporting Summary

Transparent Peer Review file

## Source data

Source Data 1

Source Data 2

## Data Availability

Unless otherwise stated, all data supporting the results of this study can be found in the article, supplementary, and source data files. Atomic coordinates of [*PSI*+]-S7 structure have been deposited in the Protein Data Bank under accession number 21BQ. The corresponding cryo-EM density map has been deposited in the Electron Microscopy Data Bank under accession code EMD-67558. Source data are provided with this paper.
