# Supplementary material for: Cryo-EM structure of ex vivo Sup35 yeast prion and the factors defining prion phenotype
Source: Nat Commun. 2026 Sep 19;17:10978. doi: 10.1038/s41467-026-77823-3 (PMC13683386; doi:10.1038/s41467-026-77823-3)
Supplement: Supplementary file 1 [file 41467_2026_77823_MOESM1_ESM.pdf]

Supplementary information

Cryo-EM structure of *ex vivo* Sup35 yeast prion and the factors defining prion phenotype

Alexander A. Dergalev[1,#,§], Yuri M. Chesnokov[2,#], Anna D. Burtseva[1,3], Olga V. Mitkevich[1], Timur N. Baymukhametov[2], Vladimir O. Popov[1,2], Konstantin M. Boyko[1,§], Vitaly V. Kushnirov[1,#,§]

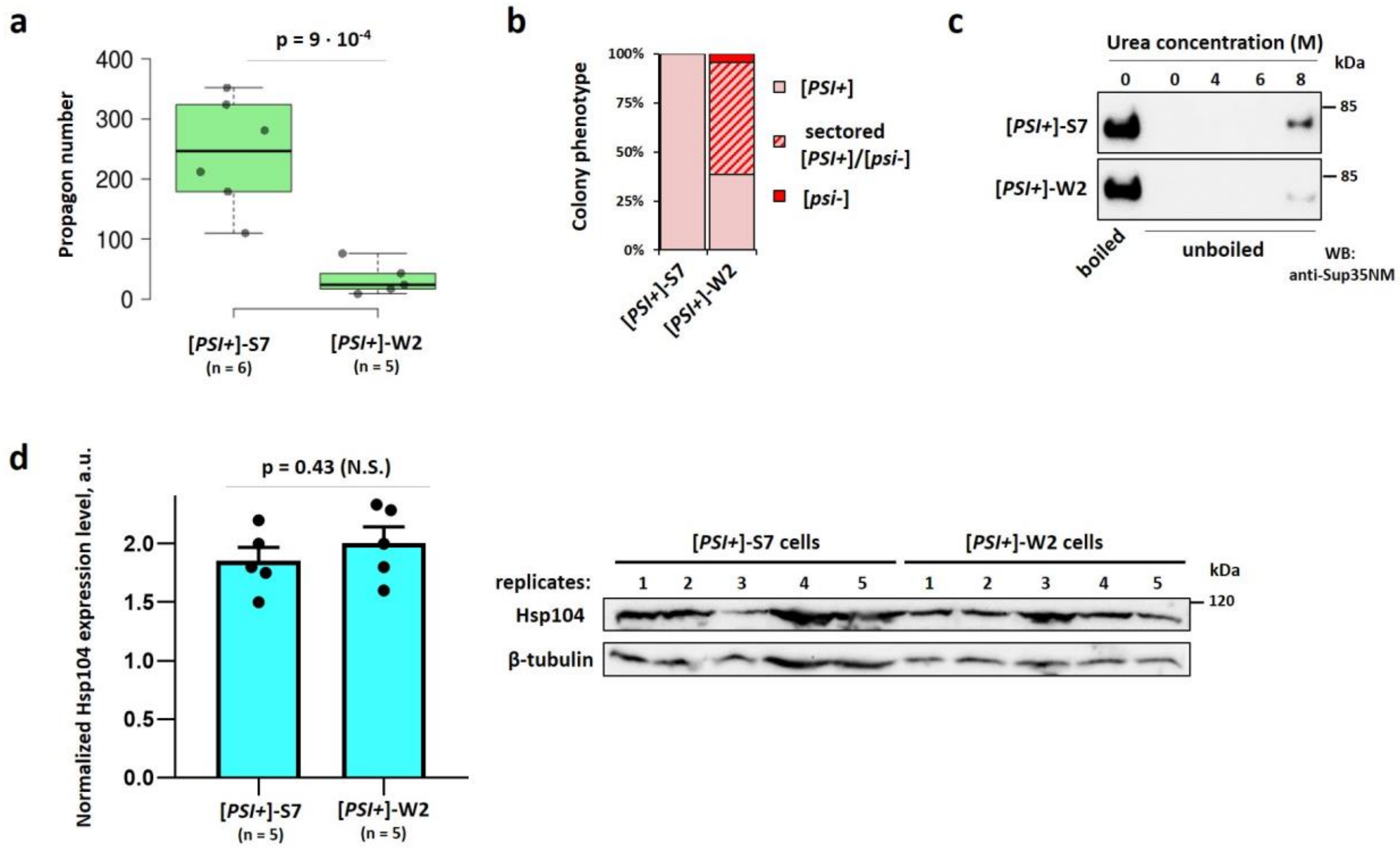

Supplementary Figure 1. Properties of the [*PSI*+]-S7 and [*PSI*+]-W2 prion variants.

a Comparison of the propagon number per cell for the [*PSI*+]-S7 and [*PSI*+]-W2 prion variants. P-value was calculated using a two-tailed Student's t-test. Center lines show the medians; box limits indicate the 25th and 75th percentiles as determined by R software; whiskers extend 1.5 times the interquartile range from the 25th and 75th percentiles.

b Mitotic stability of the [*PSI*+]-S7 and [PSI+]-W2 prion variants after a 30 min heat shock at 45°C. Data represent mean values from n = 3 biological replicates.

c Comparison of urea resistance for [*PSI*+]-S7-and [*PSI*+]-W2-templated Sup35NM-GFP purified fibrils. Equal fibril samples were incubated at 37°C for 8 hours in the presence of different urea concentrations, and the proportion of released monomeric Sup35NM-GFP molecules was determined via SDS-PAGE. A boiled sample without urea was used as a measure of the total amount of Sup35NM-GFP in the fibrils.

d Quantification of the production level of Hsp104 in [*PSI*+]-S7 and [*PSI*+]-W2 cells. Lysates of the corresponding cells were subjected to SDS-PAGE, followed by Western Blotting and immunostaining using primary antibodies against Hsp104 and β-tubulin (a housekeeping

protein). The intensities of the Hsp104 bands were normalized against the corresponding β-tubulin bands. Data represent mean ± SEM.

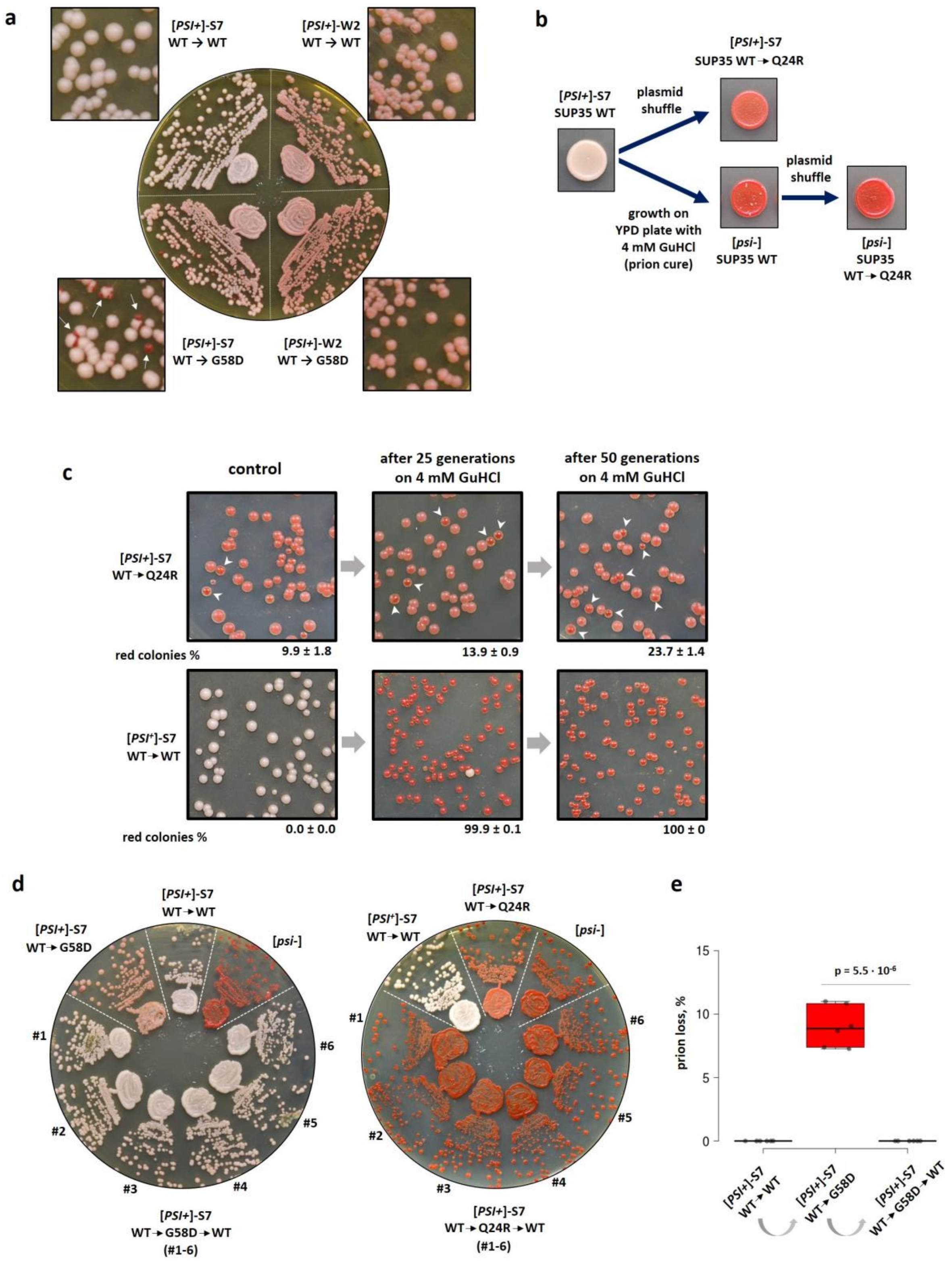

Supplementary Figure 2. The effect of G58D and Q24R substitutions on the cells carrying [*PSI*+]-S7 and [*PSI*+]-W2 prion variants.

a Nonsense-suppression phenotype of [*PSI*+]-S7 and [*PSI*+]-W2 cells where the $SUP35^{WT}$ allele was shuffled to the $SUP35^{G58D}$ allele. The cells were grown on YPD-red plates.

Colonies with a red color phenotype indicating the loss of the prion state are highlighted with white arrows.

b Comparison of the nonsense-suppression phenotypes of [*PSI*+]-S7 and [psi-] cells with SUP35^{WT} → SUP35^{Q24R} shuffle.

c Growth on YPD medium containing 4 mM GuHCl partially destabilizes the nonsense suppression

phenotype of [*PSI*$^{+}$]-S7 cells with the SUP35^{WT} → SUP35^{Q24R} shuffle. In contrast, the nonsense suppression phenotype of [*PSI*+]-S7 cells with the SUP35^{WT} allele was quickly and entirely cured under the same conditions. White arrowheads indicate red colonies losing the weak nonsense suppression phenotype obtained in [*PSI*+]-S7 cells with the SUP35^{WT} → SUP35^{Q24R} shuffle.

d-e Reverse shuffle SUP35^{G58D} → SUP35^{WT} restores of initial [*PSI*$^{+}$]-S7 phenotype (including the colony color phenotype (**d**) and mitotic stability (**e**)), whereas the reverse shuffle SUP35^{Q24R} → SUP35^{WT} causes a loss of the nonsense suppression phenotype of [*PSI*$^{+}$]-S7^{Q24R} cells. P-value was calculated using two-tailed Student's t-test. Center lines show the medians; box limits indicate the 25th and 75th percentiles as determined by R software; whiskers extend 1.5 times the interquartile range from the 25th and 75th percentiles.

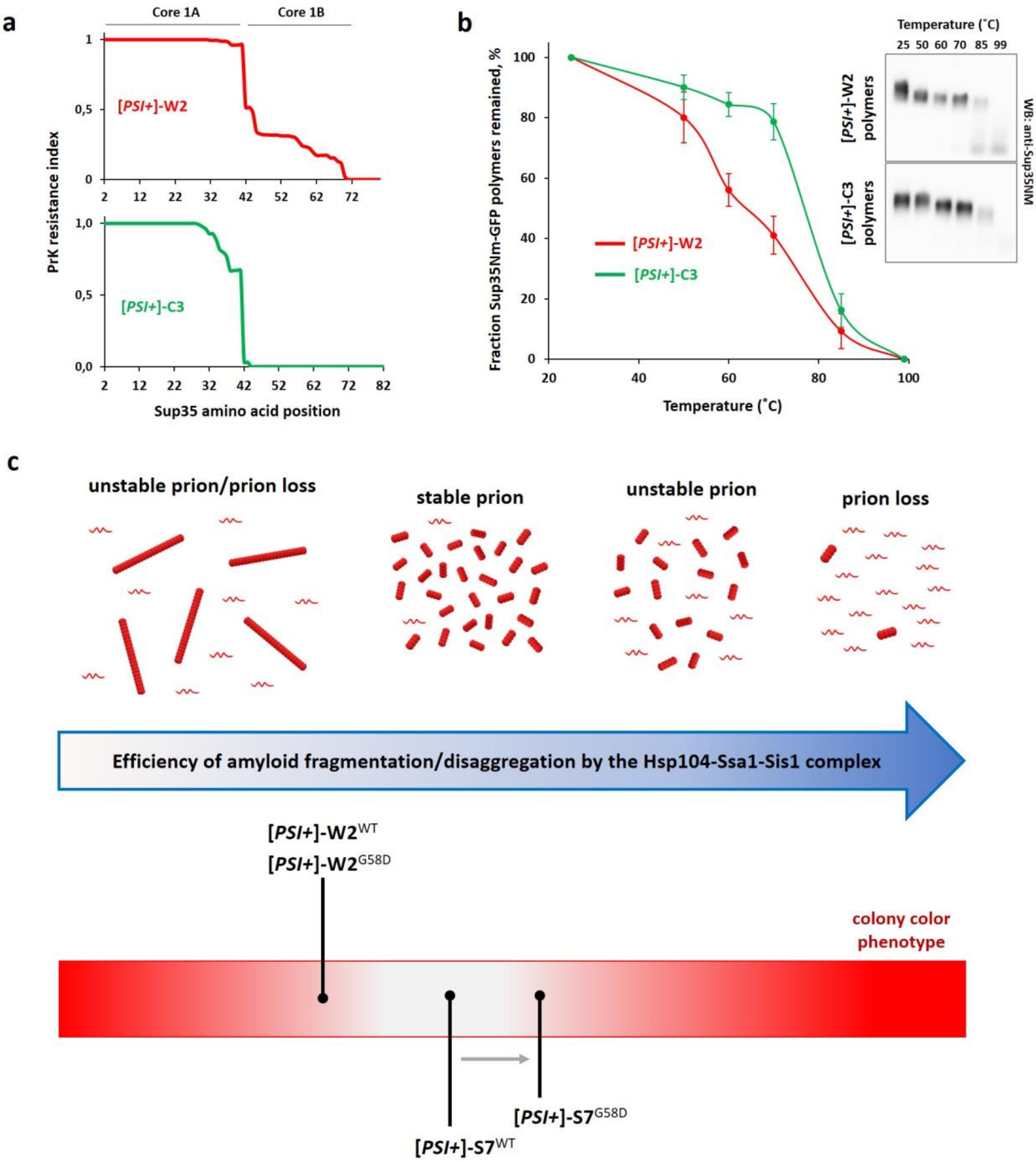

Supplementary Figure 3. A comparison of fibril stability for the weak prion variants [*PSI*+]-W2 and [*PSI*+]-C3.

a-b Comparison of PK resistance (**a**) and thermal stability (**b**) profiles of the weak prion variants [*PSI*+]-W2 and [*PSI*+]-C3. In **b**, data represent mean ± SEM (n ≥ 4 replicates).

c Scheme showing how both increased and decreased fragmentation can lead to the weakening of the nonsense suppression phenotype (i.e. a shift in colony color from white to pink).

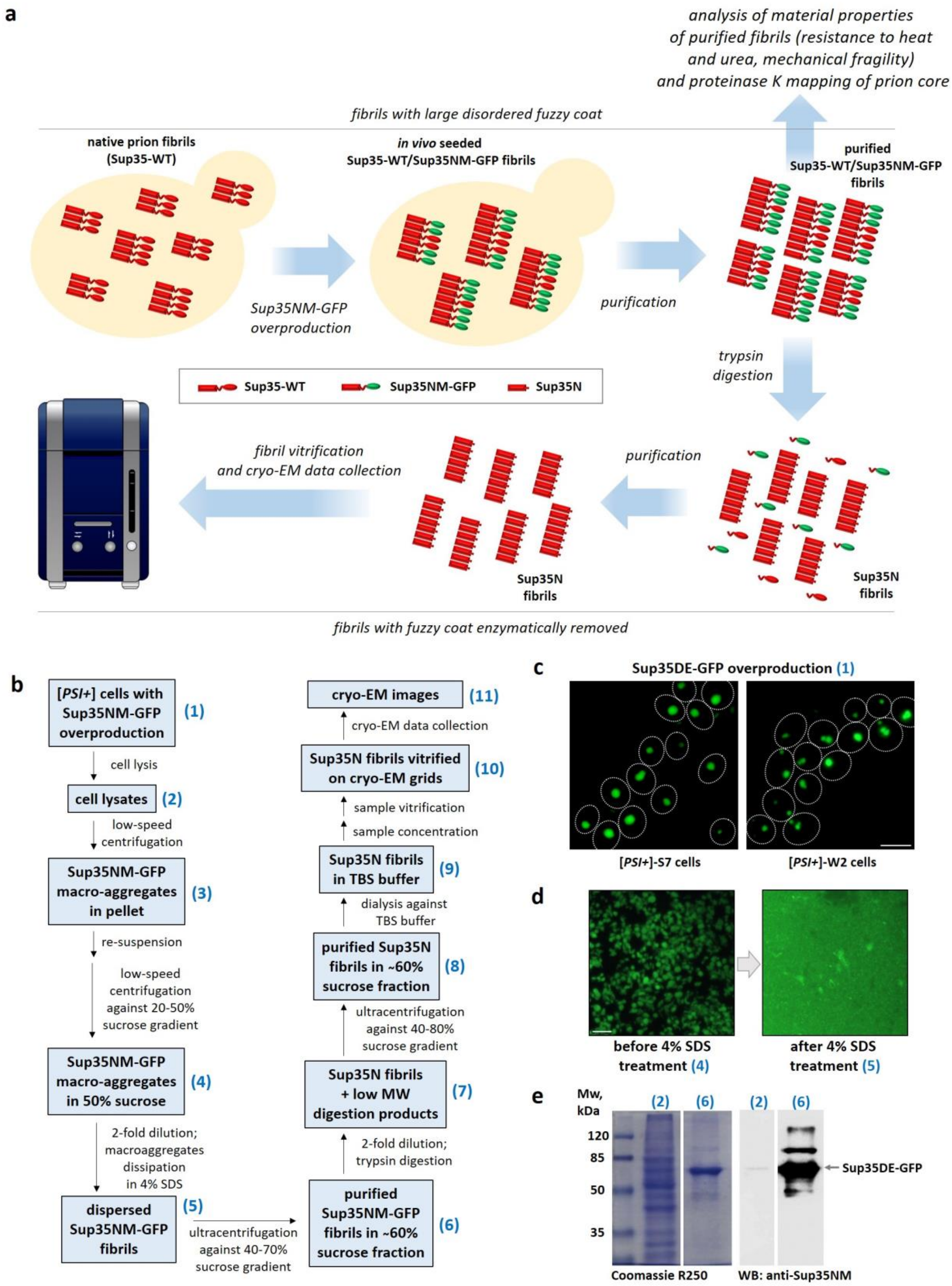

Supplementary Figure 4. Extraction of prion fibrils for the cryo-EM analysis.

a Scheme of fibril’s *in vivo* elongation, isolation and fuzzy coat cleavage as steps for obtaining particles appropriate for 3D structure reconstruction via cryo-EM images. The

desktop_electron_microscope icon by DBCLS https://togotv.dbcls.jp/en/pics.html is licensed under CC-BY 4.0 Unported https://creativecommons.org/licenses/by/4.0/.

b Graphic protocol of fibril purification and cryo-EM sample preparation.

c Fluorescence microscopy images of [*PSI*+]-S7 and [*PSI*+]-W2 cells taken after the overnight overproduction of Sup35DE-GFP. These cells were subsequently used for prion polymer extraction for the cryo-EM analysis. Scale bar is 5 μM.

d Dissolution of higher-order Sup35DE-GFP macroaggregates into individual fibrils after the addition of 4% SDS, as monitored via the fluorescence microscopy. Note that the size of the individual prion filaments is smaller than the resolution limit of conventional fluorescence microscopy; therefore, only the evenly distributed green fluorescence can be seen after the dissolution of the macroaggregates. Scale bar is 5 μM.

e Degree of purity of Sup35DE-GFP fibrils isolated from yeast cells for a cryo-EM study.

The numbers in brackets in **c**, **d** and **e** correspond to the stages of the sample preparation protocol assigned in **b**. In **a** and **b**, the protein is referred to as Sup35NM-GFP to indicate which parts of the prionogenic domain are present at the different stages of sample preparation. However, to collect the cryo-EM data, this protocol was applied to the prion fibrils composed of Sup35DE-GFP protein, which is a derivative of the Sup35NM-GFP protein with an acidic D/E cluster introduced to prevent fibril clumping after trypsin digestion.

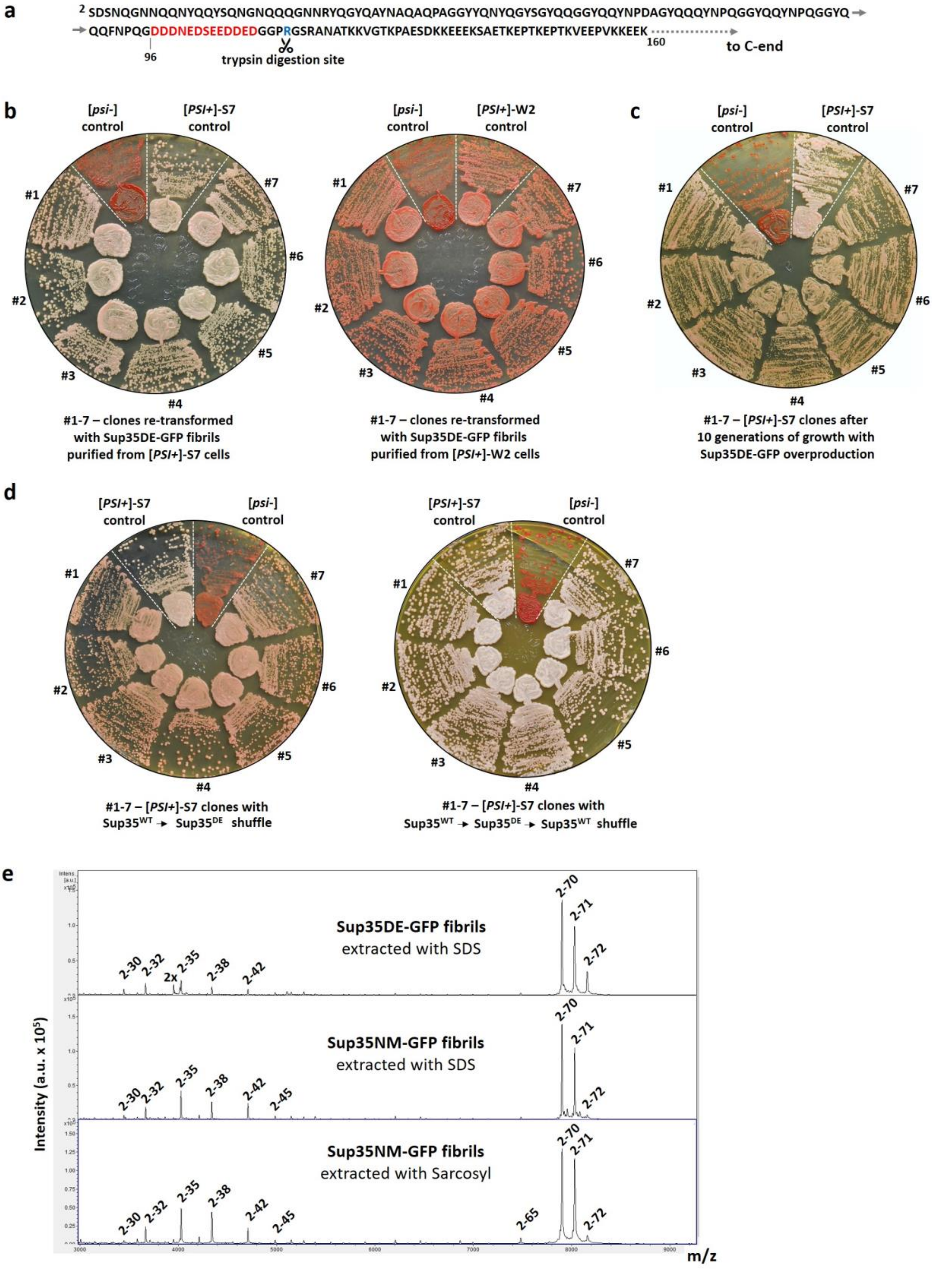

Supplementary Figure 5. Extracted Sup35DE-GFP fibrils preserve the variant-specific structure and properties of [*PSI*+]-S7 or [*PSI*+]-W2 prions.

a Sequence of the N-terminal region of the Sup35DE-GFP construct that formed [*PSI*+] prion polymers, which were used for the cryo-EM analysis. The negatively charged D/E cluster is highlighted in red and the first arginine residue available for trypsin digestion (i.e. outside the fibril core region, residues 2–72, as assessed by PK digestion) is highlighted in blue.

b [*psi*-] cells were transformed by Sup35DE-GFP prion fibrils, formed in either [*PSI*+]-S7 or [*PSI*+]-W2 cells and purified for the cryo-EM analysis, to verify the preservation of the variant-specific prion properties in the prion fibrils obtained via Sup35DE-GFP overproduction.

c The strong nonsense suppression phenotype of [*PSI*+]-S7 cells was preserved after ~ 10 generations of cell growth on an SD-Galactose medium with Sup35DE-GFP overproduction.

d Plasmid shuffle $SUP35^{WT} \rightarrow SUP35^{DE}$ results in the cells that retain the prion state (albeit with a weakened nonsense suppression phenotype), whereas the reverse shuffle $SUP35^{DE} \rightarrow SUP35^{WT}$ restores the initial [*PSI*+]-S7 phenotype.

e MALDI spectra of PK-resistant peptides of [*PSI*+]-S7 prion fibrils composed of either Sup35NM-GFP or Sup35DE-GFP proteins, and extracted using a protocol with either SDS or Sarcosyl as a detergent.

In **b-d**, control [*PSI*+]-S7, [*PSI*+]-W2 and [*psi*-] cells, as well as a representative set of clones after the corresponding treatment, were streaked on YPD-red medium.

a

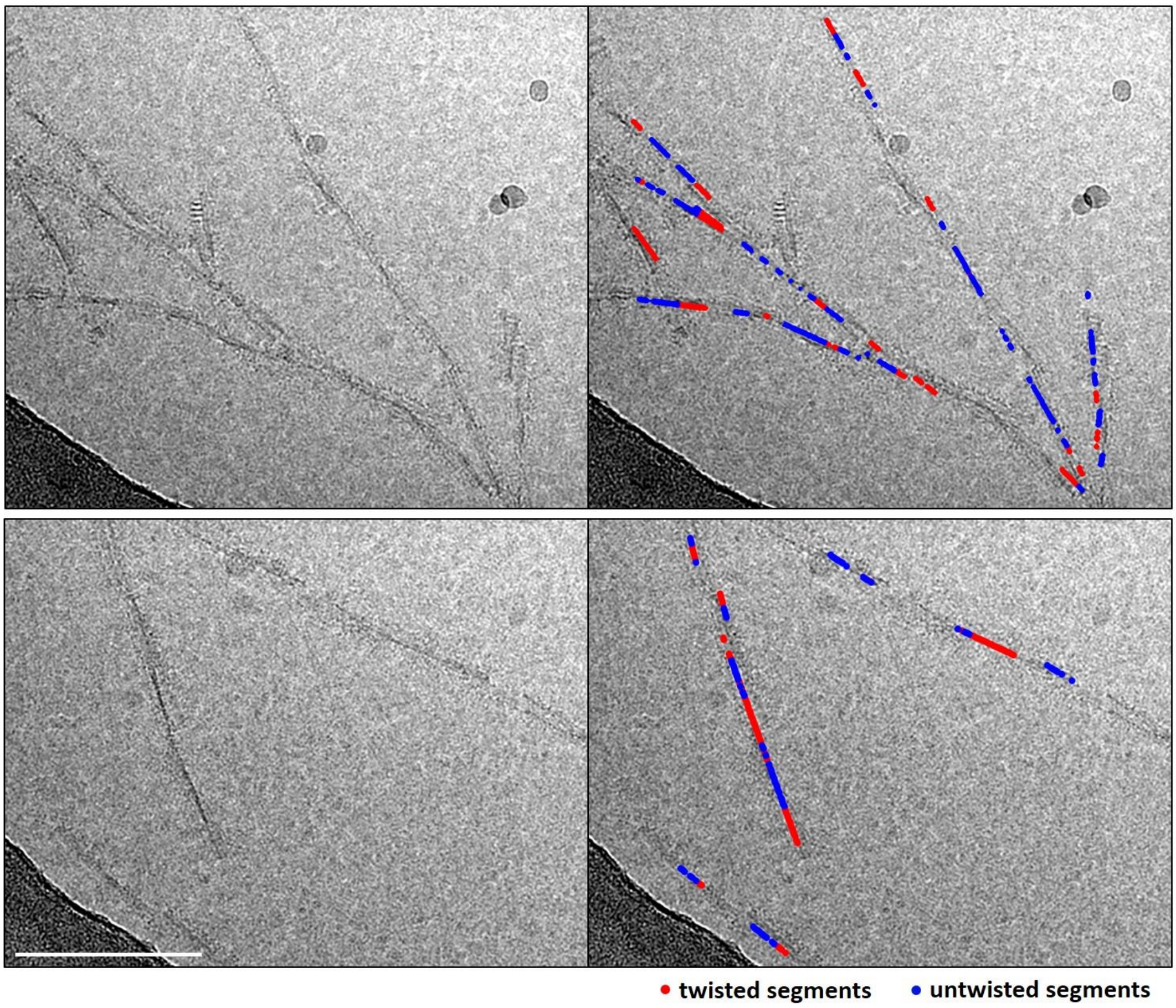

b

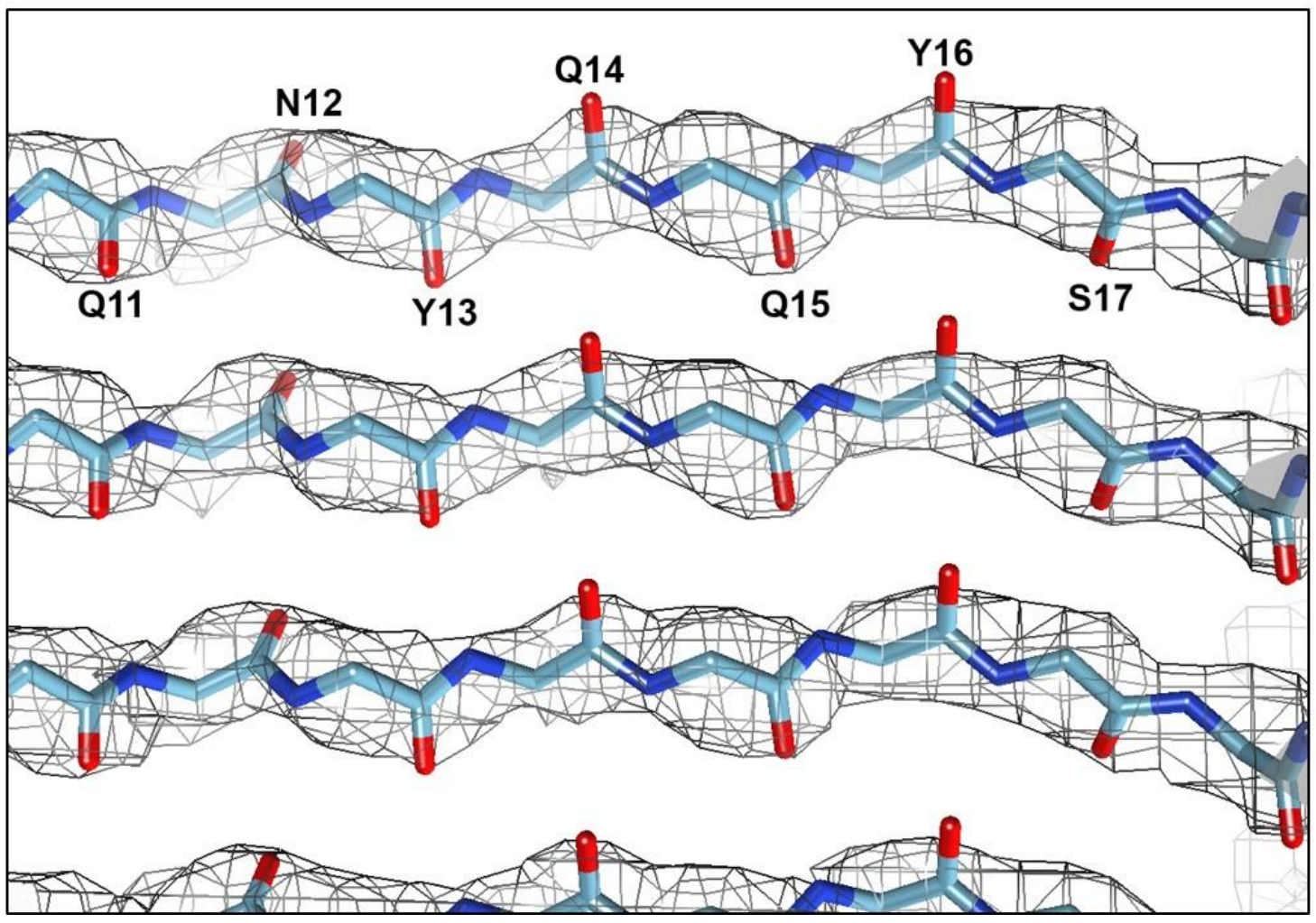

Supplementary Figure 6. A twist evaluation in the [*PSI*+]-S7 prion fibrils.

a Representative cryo-EM micrographs of [*PSI*+]-S7 prion fibrils showing the location of segments classified as twisted (red) or untwisted (blue) after the first 3D classification. Scale bar: 100 nm.

b Close-up view of the [*PSI*+]-S7 cryo-EM density map (shown as a transparent surface) with the atomic model shown as sticks (residues 12-17, carbonyl oxygens colored red), showing perfect agreement between the oxygen atoms and their corresponding density protrusions. This confirms that the [*PSI*+]-S7 fibrils have the left-handed twist that was assumed for the structure reconstruction.

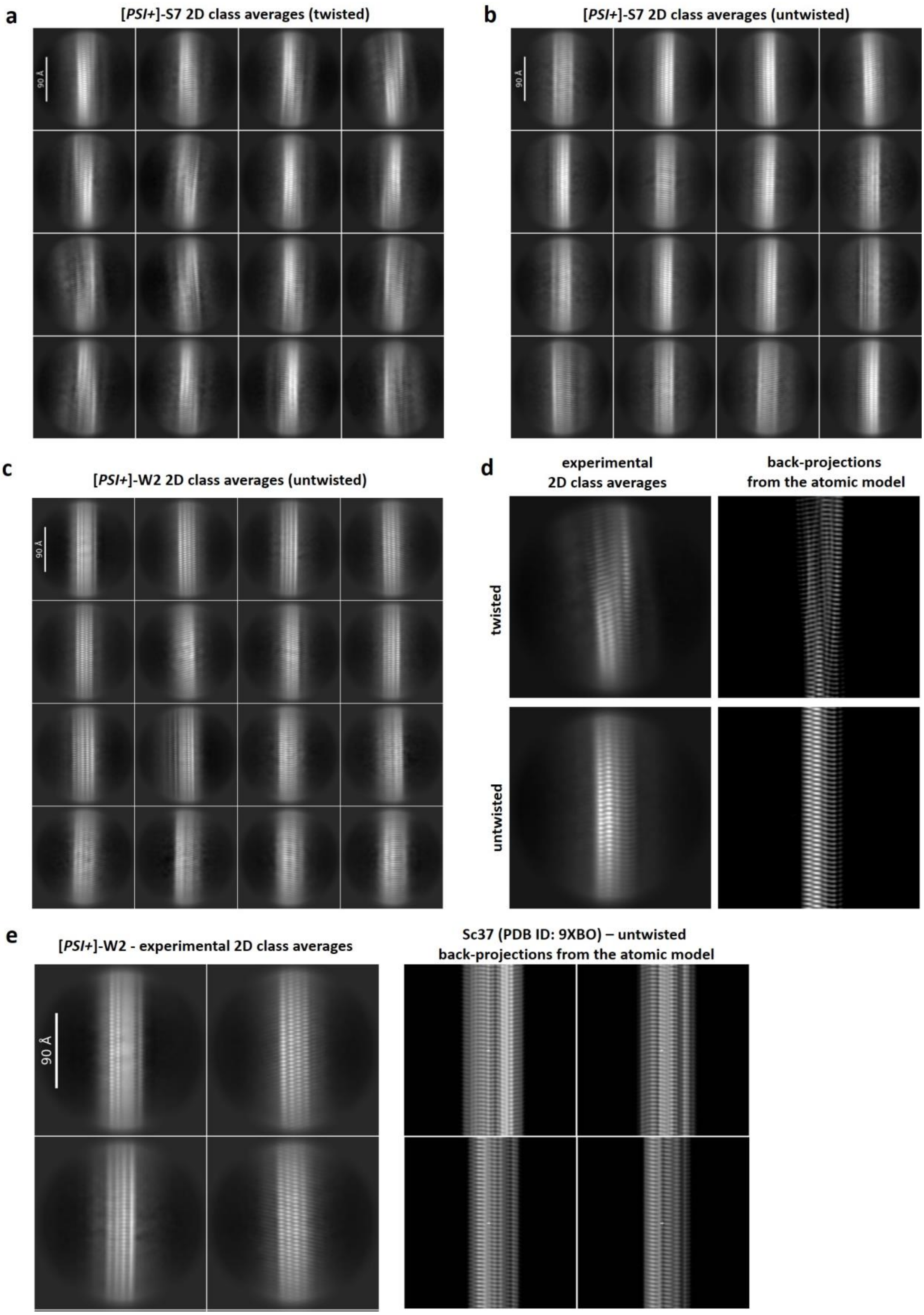

Supplementary Figure 7. 2D class averages of [*PSI*+]-S7 and [*PSI*+]-W2 prion fibrils.

a-c Representative two-dimensional class averages of the twisted [*PSI*+]-S7 (**a**), untwisted [*PSI*+]-S7 (**b**) and untwisted [*PSI*+]-W2 (**c**) fibril segments. The preparation of the [*PSI*+]-S7 prion contained both twisted and untwisted segments, whereas the preparation of the [*PSI*+]-W2 prion contained only untwisted segments.

d Experimental 2D class averages of twisted and untwisted segments from [*PSI*+]-S7 fibrils compared with twisted and untwisted back-projections from the [*PSI*+]-S7 atomic model.

e Experimental 2D class averages of untwisted [*PSI*+]-W2 fibrils compared with untwisted back-projections from the atomic model of Sc37 amyloid (PDB ID: 9XBO; reported by Tanaka et al., 2025[13]).

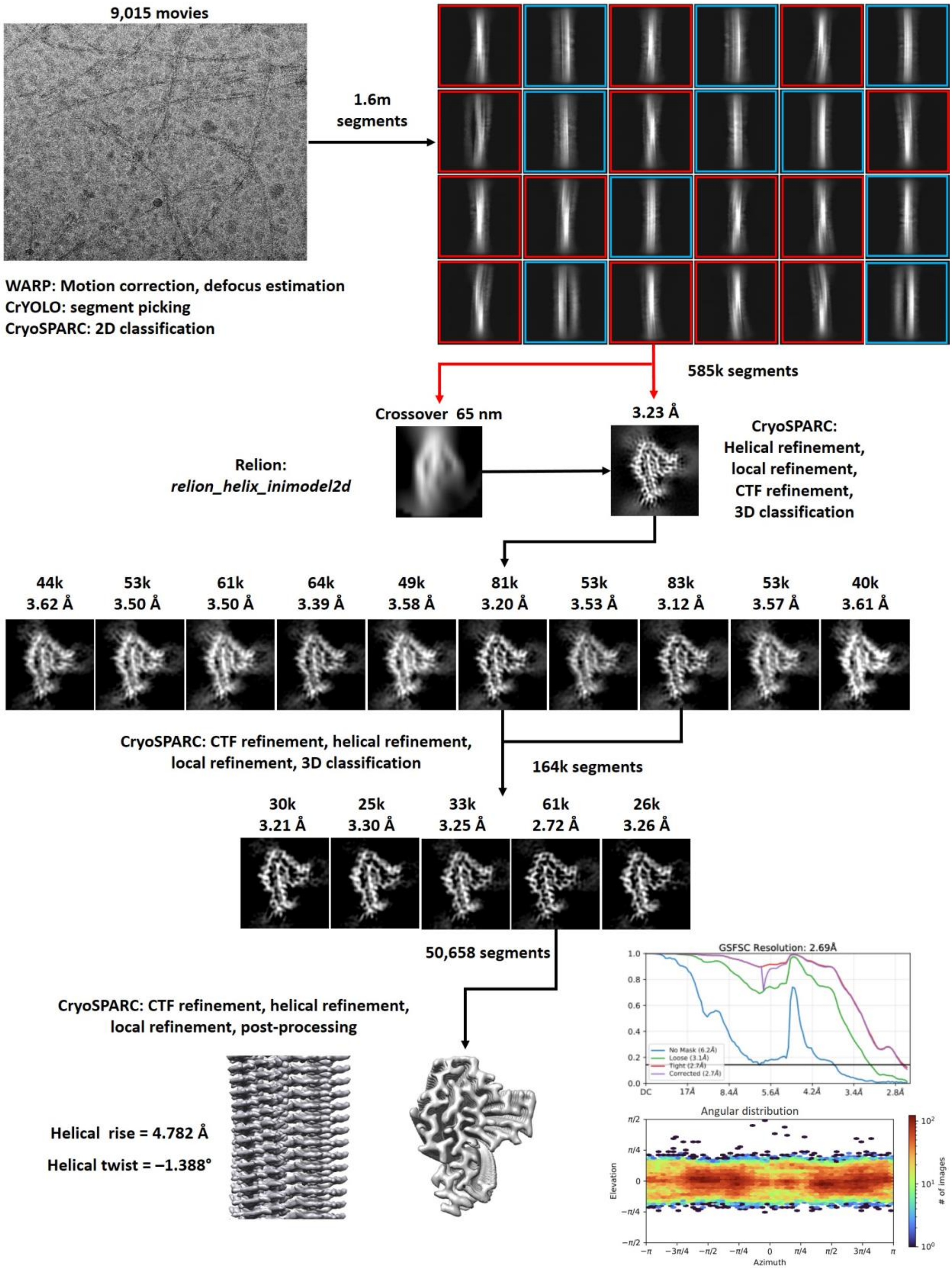

Supplementary Figure 8. Schematic representation of the cryo-EM data collection and processing pipeline.

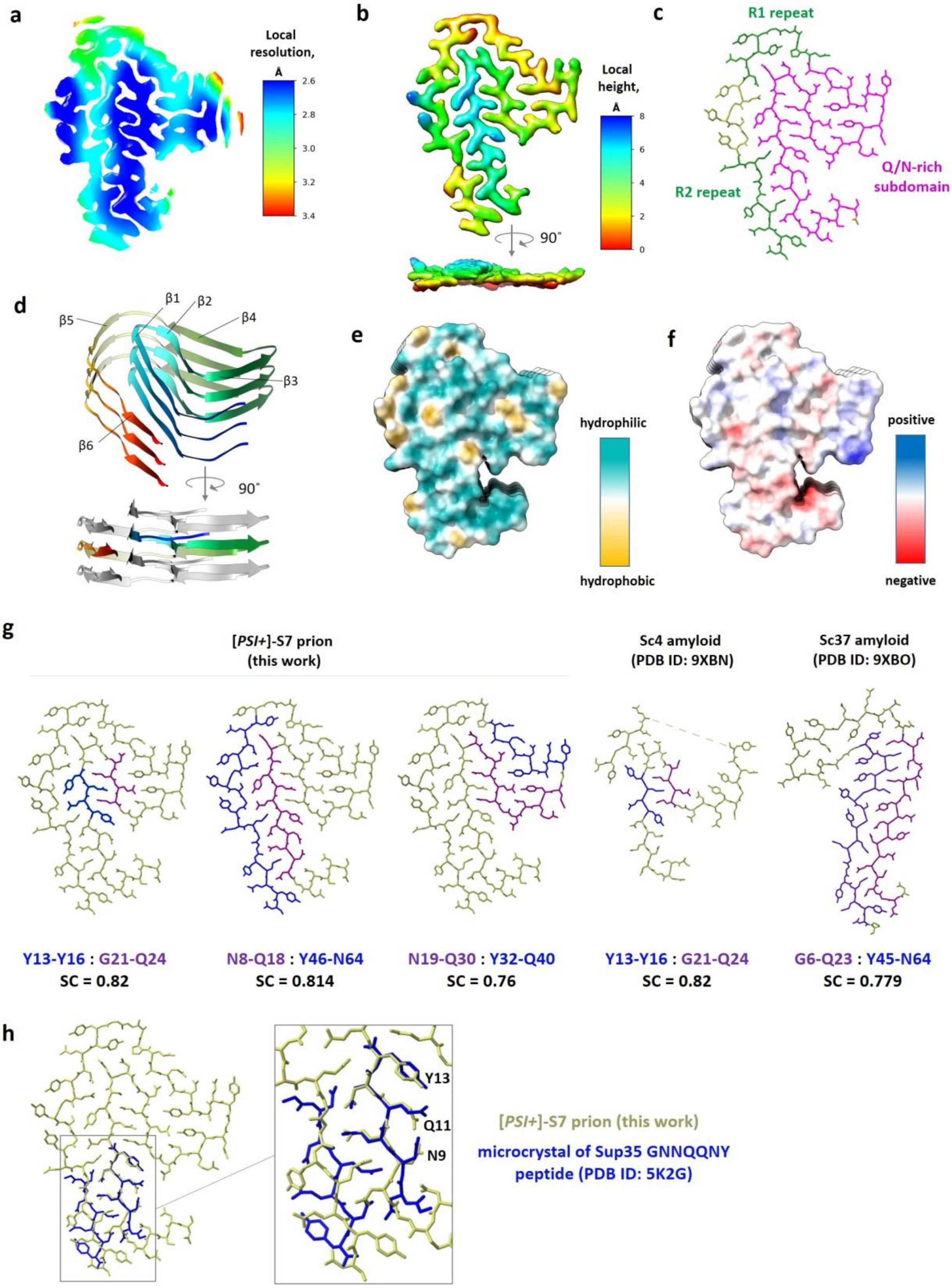

Supplementary Figure 9. Properties of the [*PSI*+]-S7 prion structure.

a-b Local resolution (**a**) and local height (**b**) maps of the [*PSI*+]-S7 prion structure.

c The Q/N-rich subdomain and the R1 and R2 oligopeptide repeats superimposed over the [*PSI*+]-S7 prion structure.

d The β–strand arrangement of the [PSI+]-S7 fibril core structure.

e-f Electrostatic (**e**) and hydrophilic (**f**) surface representations of the structure of a [*PSI*+]-S7 prion.

g Representation of the shape complementarity (SC) parameter values calculated for the pairs of segments in the structures of [*PSI*+]-S7, Sc4 (9XBN) and Sc37 (9XBO) structures.

h Alignment of the structures of [*PSI*+]-S7 prion and the GNNQQNY peptide from Sup35 protein (PDB ID: 5K2G).

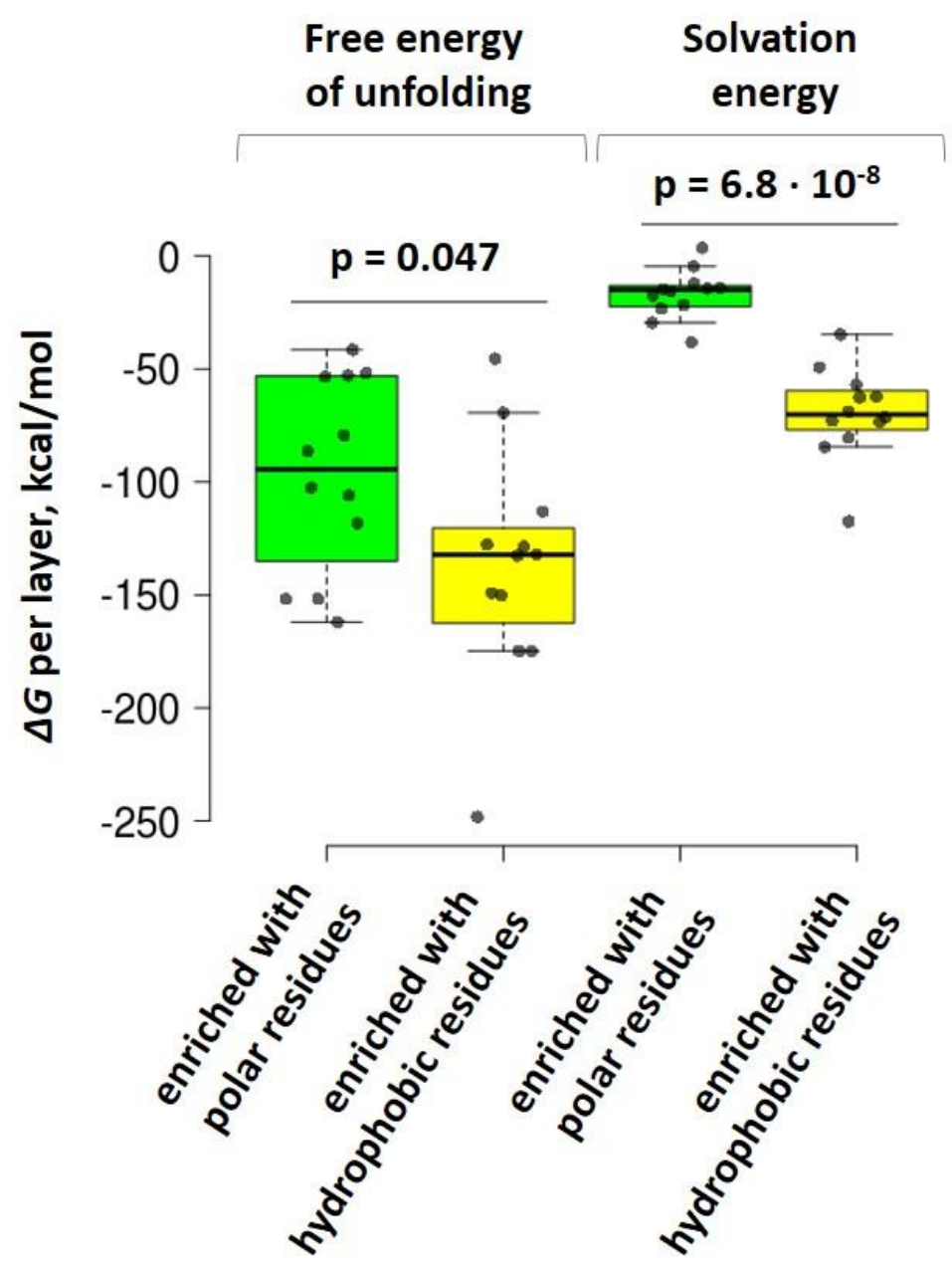

Supplementary Figure 10. A comparison of two computational methods of amyloid thermodynamic stability evaluation used in this work.

Comparison of the free energy of unfolding (calculated with FoldX) and solvation energy (calculated with Amyloid Illustrator package), determined for a set of amyloid structures deposited at Protein Data Bank and classified as “enriched with polar residues” (PDB ID: 6VPS, 7ZIR, 5W3N, 7VQQ, 6XFM, 8DUW, 8DU2, 6WQK, 8ONS, 8TEQ, 8TER, and the Sup35 prion structure determined in this work) or “enriched with hydrophobic residues” (PDB ID: 8EFU, 9DMZ, 7UMQ, 7P6C, 7NRT, 5O3L, 9E9X, 6XYQ, 6XYO, 8KF5, 9CZP, 6SHS) and normalized for a single amyloid layer. Center lines show the medians; box limits indicate the 25th and 75th percentiles as determined by R software; whiskers extend 1.5 times the interquartile range from the 25th and 75th percentiles. P-values were calculated using a two-tailed Student’s t-test.

Supplementary Table 1. Primers used for the plasmid construction.

Name	Sequence	Comment
**PNM2-D**	CCAACAAGATGGCTATCAACAGTACAATCC	G58D mutation to pRS315-SUP35
**PNM2-R**	TGATAGCCA**T**CTTGTTGGTACCCAGAATAAC	G58D mutation to pRS315-SUP35
**S17R-D**	GCAATACAGGCAGAACGGTAACCAACAAC	S17R mutation to pRS315-SUP35
**S17R-R**	CCGTTCTGCCTGTATTGCTGGTAGTTTTGC	S17R mutation to pRS315-SUP35
**Q24R-D**	CCAACAAAGAGGTAACAACAGATACCAAGG	Q24R mutation to pRS315-SUP35
**Q24R-R**	CTGTTGTTACCTCTTTGTTGGTTACCGTTCTGGC	Q24R mutation to pRS315-SUP35
**Sup35DE-Rf**	CCTTCTTGGTAGCATTGGCACGGGATCCACGCGGACCACCATCCTCGTCGTCTTCCTCACTGTCCTCGTTGTCGTCGTCGCCTTGTGGATTGAATTGC	Acidic DE fragment insertion to pYES2-SUP35NM-GFP
**Pxd22**	TTATTCTGGGTACCAACAA	DE fragment insertion to pYES2-SUP35NM-GFP

Supplementary Table 2. Cryo-EM data collection, reconstruction and model statistics

**Data collection**	**Sup35 [*PSI*$^{+}$]-S7 fibril**
Magnification	×81,000
Defocus range (μm)	–0.7 to –2.0
Voltage (kV)	300
Microscope	Titan Krios
Camera	Gatan K3
Energy filter	BioQuantum, 20 eV slit
# movie frames	70
Total electron dose (e^{-}/Å^{2})	52
Pixel size (Å)	0.863
# micrographs	9015
**Reconstruction**	**Sup35 [*PSI*$^{+}$]-S7 fibril**
Box size (pixel)	384

Inter-box distance (Å)	24
# segments extracted	1,259,268
# segments after Class2D	585,256
# segments after Class3D	50,658
Resolution (Å)	2.7
Map sharpening B-factor (Å²)	–58
Helical rise (Å)	4.782
Helical twist (°)	–1.388
**Atomic model**	**Sup35 [*PSI*⁺]-S7 fibril**
Chains	5
# unique non-hydrogen atoms (5 chains)	2580
Protein residues (5 chains)	315
R.m.s.d. bonds (Å)	0.005
R.m.s.d. angles (°)	0.503
Clashscore	7.72
MolProbity score	1.86
Ramachandran favored (%)	96.72
Ramachandran allowed (%)	3.28
Ramachandran outliers (%)	0.00
Rotamer outliers (%)	2.04
Model resolution (Å) (FSC>0.143/0.5)	2.7/3.2

Supplementary Notes

Characterization of [*PSI*+] prion variants

Previously, we demonstrated that "strong" and "weak" [*PSI*+] variants constitute two distinct classes of Sup35 prion, which can be differentiated based on their nonsense-suppressor phenotype, structural characteristics, and their response to the overproduction of SUP35 and HSP104 genes[1]. For the present study, we selected one representative of each class from this previous work: [*PSI*+]-S7 and [*PSI*+]-W2, respectively. To evaluate how these variants correspond to the strong and weak [*PSI*+] variants described earlier, we assessed some phenotypic features of these [*PSI*+] variants. Consistent with previous studies, the [*PSI*+]-W2 variant exhibited a larger polymer size (Fig. 1b) and a higher proportion of soluble Sup35 than the [*PSI*+]-S7 variant (Fig. 1c). This correlates with a weaker nonsense suppressor phenotype (Fig. 1a) and indicates less frequent fragmentation of [*PSI*+]-W2 prion fibrils. The number of propagons (units of prion inheritance) in [*PSI*+]-W2 cells was lower

than in [*PSI*+]-S7 cells (Supplementary Fig. 1a). Unlike some previously described weak variants[2], [*PSI*+]-W2 exhibited high mitotic stability under standard conditions (prion loss of less than 10^{-3} per 25 generations for both variants). However, as was demonstrated for other weak variants[3,4], the mitotic inheritance of [*PSI*+]-W2 was significantly impaired following short-term heat shock (Supplementary Fig. 1b). Therefore, we can conclude that [*PSI*+]-S7 and [*PSI*+]-W2 generally exhibit the phenotypic properties of strong and weak prion variants that have been studied previously.

[*PSI*+]-S7 prion fibril structure

Validation of helical handedness

Since cryo-EM reconstruction does not provide information on the absolute handedness, the final map may need to be inverted. Although most amyloid filaments solved to date have a left-handed twist, filaments with right-handed twists have also been observed[5]. Prior to helical refinement, we therefore assumed a left-handed twist by default. At local resolutions beyond 2.9 Å, the handedness can be determined directly from the map by examining the densities of the carbonyl oxygens in the main chain. In our reconstruction, several regions of the ordered core reach a local resolution of approximately 2.6 Å, enabling such a direct assessment. As shown in Supplementary Fig. 6b (residues 12-17), the cryo-EM density displays a distinct zig-zag pattern perpendicular to the β-strand direction and along the fibril axis. This pattern arises from the alternating orientation of the carbonyl groups. The perfect agreement between the atomic model and the map — with each carbonyl oxygen atom (coloured red) residing inside its corresponding density protrusion — unambiguously confirms that our initial choice of a left-handed twist is correct. This high-resolution feature is consistently observed across other well-resolved regions of the map, further supporting this assignment.

Lateral stabilization interactions

A quantitative analysis of the interface geometry using the shape complementarity (SC) parameter reveals that both of the main steric zipper interfaces display a high level of surface complementarity. The inner steric zipper, formed by interactions between residues 13–17 and 20–24,

has an SC value of 0.82 and is characterized by a regular, checkerboard-like alternation of polar side chains. Residues 13 and 15 on one strand interdigitate with residues 22 and 24 on the opposing strand (see Fig. 3f, top panel). The outer steric zipper forms a more extensive interface involving residues 8–18 on one side and 46–63 on the opposite side of the protomer. Unlike the inner zipper, this interface lacks a continuous checkerboard arrangement along its entire length. Nevertheless, the outer zipper's overall surface complementarity remains high (SC = 0.814), due to a combination of volumetric interdigitation and local groove-mediated packing motifs within the zipper interface. In single β-layers, these motifs correspond to concave surface features enriched in small residues, such as glycine or serine, which lack long polar side chains. Upon multilayer stacking, these features align along the fibril axis to form continuous surface grooves along the fibril axis that can accommodate multiple side chains from the opposing strand. Within the outer steric zipper, two such groove-mediated motifs are observed: one involving residues Q10 and N12 that insert into a groove formed by residues G58 and G59 with residues Q57 and Q61 shaping the groove boundaries (SC = 0.83); and a second involving residues Q14 and Y16 that insert into a groove defined by residues S53 and G54 with residues Q50 and Q57 shaping the groove boundaries (SC = 0.85; see Fig. 3f, bottom panel). Together, these groove-mediated packing motifs compensate for the lack of a regular checkerboard pattern in some areas of the outer steric zipper. This enables the formation of a continuous, highly complementary zipper interface, despite the fact that the surfaces that interact with each other are predominantly polar.

Unlike the high SC values observed for the inner and outer steric zippers, the three-strand region spanning residues 19–44 does not form steric zipper interfaces and exhibits substantially lower surface complementarity. Similarly, the alanine-rich β-strand spanning residues 34–42 exhibits only moderate SC values in the central region of the fibril (SC = 0.78 between residues 19–31 and 34–42; SC = 0.76 between residues 19–30 and 32–40). Despite the presence of alanine residues at positions A34, A37 and A39, these side chains are short and weakly penetrating, and are therefore insufficient to support highly complementary steric zipper packing.

Short Sup35-derived peptides are known to form fibrils and microcrystals, and their structures have been extensively characterized using X-ray crystallography. One well-studied example is the GNNQQNY peptide (residues 7–13 of Sup35), which forms a dry steric zipper interface with high shape complementarity (SC = 0.86) in peptide microcrystal structures, but without a regular checkerboard arrangement of side chains. In these crystals, the dry steric zipper forms only on one side of the β-strand; the other side engages in hydrated, non-zipper contact with neighboring peptides. In the [PSI$^+$]-S7 fibril, a related asymmetric organization is observed: residues 7–12 form a dry steric zipper interface only on one side, contacting residues 57–63; no equivalent hydrated interface is formed on the opposite side. In contrast, the adjacent segment spanning residues 13–17 participates in two dry steric zipper interfaces simultaneously, forming part of both the inner and outer zippers, and occupying a central position within the fibril core.

In addition to the general stabilization of the amyloid core, which is achieved through the tight geometric matching of opposing surfaces, some auxiliary polar interactions further stabilize these interfaces at turns and kinks. Notably, a slipped π-π interaction between Y29 and Y35, characterized by an interplanar separation of ~3.1 Å and a pronounced in-plane offset of ~4.4 Å, provides only modest π-overlap in a single protomer, but becomes cumulatively stabilizing when replicated across successive layers of the fibril. This supports the hairpin turn and reinforces the central region of Core1A. By contrast, the outer zipper exhibits fewer intra-layer side-chain-to-side-chain contacts, but contains a clear Y45 ↔ Q47 hydrogen bond that anchors the β-strand of Core1B against the rest of the fold. Along with the dense volumetric interdigitation across the staggered interfaces, these local interactions form a continuous, intramolecular stabilizing network within a protomer, thus consolidating the fold in a plane perpendicular to fibril elongation.

“Vertical” stabilization interactions (between stacked layers)

Alongside the backbone cross-β hydrogen bond ladder, two additional inter-layer elements reinforce axial stabilization. Firstly, Q/N polar ladders, formed by aligned side-chain donors and acceptors (ND2/NE2 ↔ OD1/OE1), run parallel to the axis. Examples include Q22, Q24 and Q33;

N21 and N48; and Q56 and Q57. These ladders strengthen the registry, especially within residues 6–29 and 46–60. Secondly, side-chain to backbone hydrogen bonds across layers provide additional stabilization at the inner interface, including Q22 → O(Y16) and Q24 → O(Q14). Thirdly, aromatic stacking provides a further major source of axial reinforcement: twelve tyrosines (Y13, Y16, Y29, Y32, Y35, Y45, Y46, Y49, Y52, Y55, Y60 and Y63) form highly regular, parallel, displaced π-π columns that extend across layers. These stacks have centroid-to-centroid distances of 4.8–4.9 Å, interplanar separations of 3.0–3.6 Å and in-plane slip values of 3.2–3.8 Å. The ring normals are tilted by approximately 32–52° (typically near 45°) relative to the fibril axis, which optimises π-overlap under lateral displacement.

Peripheral surface features and adsorption-prone patches

Beyond the disordered N- and C-terminal segments outside the ordered core, the map reveals additional diffuse densities adjacent to surface regions spanning residues 30–32 and 42–45. These regions do not exhibit the characteristic zig-zag β-strand arrangement of the amyloid backbone, nor do they form additional ordered substructures. Instead, they present relatively planar surfaces shaped by glycines, prolines and exposed tyrosines. In 2D class averages, the associated lateral density follows the helical twist of the filament and extends up to ~5 nm from the axis. This is consistent with the idea that this material remains associated with the protofilament — for example, the fuzzy-coat remnants or transiently bound molecules — but is not incorporated into the cross-β architecture. The planar topography and polar/aromatic composition of these regions provide a structural rationale for their propensity to interact with solvent or accessory molecules.

Validation of the structural identity of Sup35DE-GFP prion fibrils used for cryo-EM analysis to the fully-native [PSI+] prion polymers

The fully-native [*PSI+*] polymers composed of endogenous Sup35WT are not suitable for the cryo-EM structure reconstruction procedure due to the following: (i) their short length, making them more like "barrels" rather than "fibrils" in shape[6,7]; (ii) the structural noise imposed by the fuzzy coat with a globular domain attached to the fibril core[8]; and (iii) the high propensity of such fibrils to coaggregate

into dense multifibrillar clumps when fuzzy coat is enzymatically removed. Therefore, we were not able to obtain the clear 2D class averages of the fully-native [*PSI+*]. However, we obtained some structural and genetic evidence suggesting that the structure of [*PSI+*]-S7 prion is, at least, highly similar for the fibrils composed of either Sup35WT or Sup35DE-GFP proteins.

Firstly, we demonstrated that the PK digestion patterns of [*PSI*+]-S7 prion polymers composed of Sup35NM-GFP or Sup35DE-GFP constructs are nearly identical (Supplementary Fig. 5e). Previously, we showed that these patterns are also similar for native Sup35WT prion polymers and for *in vivo* seeded Sup35NM-GFP polymers[1]. Overall, these data suggest that the PK-resistant Core1 has a very similar span and organization in the prion fibrils formed by either Sup35WT or Sup35DE-GFP.

Secondly, we demonstrated that *in vivo* seeded Sup35DE-GFP fibrils are capable to transfer the prionic state of either strong or weak type. Spheroplast transformation of [*psi*-] yeast with Sup35DE-GFP fibrils, which were used for either [*PSI+*]-S7 or [*PSI+*]-W2 cryo-EM analyses, reproduced the initial strong and weak nonsense suppression phenotypes, respectively (Supplementary Fig. 5b). In [*PSI+*]-S7 cells, we also shuffled the wild-type Sup35 to the Sup35^{DE} construct (which is nearly identical to the Sup35DE-GFP construct used for cryo-EM, except that GFP is substituted for the Sup35C domain) produced under SUP35 promoter. After this, the [*PSI+*]-S7 prion continued to propagate, albeit with a slightly weakened nonsense suppression phenotype. Importantly, the Sup35^{DE} construct lacked the Sup35 residues 124-155, which includes half of the Hsp70 chaperones binding site that is critical for the [*PSI+*] prion propagation[9]. Therefore, attribute the reduced suppression to the truncation of this site rather than to the introduction of the D/E cluster. After the reverse Sup35^{DE} → Sup35^{WT} shuffle, the strong prion phenotype was restored (Supplementary Fig. 5d). In an alternative experiment, we allowed [*PSI+*]-S7 cells to grow for ~10 cell generations on the SD-Galactose medium with Sup35DE-GFP construct overproduced in the same manner as for the cryo-EM sample preparation. In this setup, the overproduced Sup35DE-GFP construct formed mixed fibrils with the pre-existing Sup35WT prion polymers composed mainly (~100:1) of Sup35DE-GFP. Overproduction was then stopped by plating the cells on YPD-red medium. After forming colonies (~25 generations), all of the cells eventually exhibited a strong nonsense suppression phenotype

(Supplementary Fig. 5c). Taken together, these results suggest that the derivatives of Sup35 protein with the acidic cluster can propagate the prion state and maintain the variant-specific structure.

Supplementary discussion

[*PSI*+]-S7 prion structure – general notes

The [*PSI*+]-S7 prion structure illustrates how a Q/N-rich cross-β fold can achieve high stability without a hydrophobic core. Rigidity and protease resistance arise from both van der Waals interactions in the steric zippers, and a cooperative polar-aromatic framework, where the canonical in-register cross-β backbone network is reinforced by extensive Q/N ladders and by uniformly structured, parallel-displaced π-π columns of tyrosines. Arrays of these individually modest interactions, repeated along the fibril axis, generate a stable scaffold that maintains a compact amyloid key-like topology. From this perspective, the structure of Sup35 prion resembles the structures of other amyloids enriched in polar residues, like the functional Orb2 amyloid[10] and pathology-related TAF15[11] amyloid.

This architecture revises the earlier 'serpentine' model, which proposed that the amyloid core of Sup35 consists of a continuous β-sheet formed predominantly by tandem oligopeptide repeats[12]. In Sup35, these repeats correspond to PQGGYQ-like motifs grouped in the middle of the N domain. In the [*PSI*+]-S7 fibril, two such repeats are incorporated into the ordered core. Contrary to the serpentine model, they do not form β-arches against each other. The first repeat (R1, residues ~40–48) lies within the 30–45 segment and only contributes limited connectivity to the central steric zippers. In contrast, the second repeat (R2, residues ~56–65) is fully integrated into the outer zipper, forming a stable linkage between Core1A and Core1B (Supplementary Fig. 9c). The remaining repeats are excluded from the structured spine and instead contribute to the disordered fuzzy coat. Therefore, the oligopeptide repeats are not universal linear building blocks of the Sup35 amyloid architecture, but rather participate in distinct, context-dependent roles shaped by the amyloid key-like topology and its polar-aromatic stabilization.

Distal cores in the structures of *in vitro* and *ex vivo* Sup35 fibrils.

Another interesting feature of the Sc37 structure is the presence of the distal cross-β element K102-Q132[13]. In PK digests of both weak and strong *ex vivo* prions we often observed additional cores located at either residues 90-120 (Region 2) or 125-133 (Region 3)[1]. The second core in Sc37 is different as it incorporates parts from both Regions 2 and 3. A similar element was also detected in PK digests of Sup35 prions, but only in two strong of the 26 strong and weak prion isolates studied. Notably, the distal core elements could not be found in the [*PSI+*]-S7 cryo-EM structure solved in this study because the Sup35 sequence downstream of the 96 a.a. position was enzymatically removed to facilitate structure reconstruction (Supplementary Fig. 5a).

References

1. Dergalev, A. A., Alexandrov, A. I., Ivannikov, R. I., Ter-Avanesyan, M. D. & Kushnirov, V. V. Yeast Sup35 Prion Structure: Two Types, Four Parts, Many Variants. *Int J Mol Sci* **20**, (2019).
2. Shkundina, I. S., Kushnirov, V. V., Tuite, M. F. & Ter-Avanesyan, M. D. The Role of the N-Terminal Oligopeptide Repeats of the Yeast Sup35 Prion Protein in Propagation and Transmission of Prion Variants. *Genetics* **172**, 827–835 (2006).
3. Newnam, G. P., Birchmore, J. L. & Chernoff, Y. O. Destabilization and Recovery of a Yeast Prion after Mild Heat Shock. *Journal of Molecular Biology* **408**, 432–448 (2011).
4. Klaips, C. L., Hochstrasser, M. L., Langlois, C. R. & Serio, T. R. Spatial quality control bypasses cell-based limitations on proteostasis to promote prion curing. *eLife* **3**, (2014).
5. Lövestam, S. & Scheres, S. H. W. High-throughput cryo-EM structure determination of amyloids. *Faraday Discuss.* **240**, 243–260 (2022).
6. Kryndushkin, D. S., Alexandrov, I. M., Ter-Avanesyan, M. D. & Kushnirov, V. V. Yeast [*PSI* $^{+}$] Prion Aggregates Are Formed by Small Sup35 Polymers Fragmented by Hsp104. *Journal of Biological Chemistry* **278**, 49636–49643 (2003).
7. Bagriantsev, S. N., Gracheva, E. O., Richmond, J. E. & Liebman, S. W. Variant-specific [PSI+] infection is transmitted by Sup35 polymers within [PSI+] aggregates with heterogeneous protein composition. *Mol. Biol. Cell* **19**, 2433–2443 (2008).

8. Burtseva, A. D. *et al.* Electron Microscopy Study of the Structure of the Sup35 Prion from Yeast Saccharomyces cerevisiae. *Crystallogr. Rep.* **68**, 872–878 (2023).

9. Shen, C. H. *et al.* Exposed Hsp70-binding site impacts yeast Sup35 prion disaggregation and propagation. *Proc. Natl. Acad. Sci. U.S.A.* **121**, e2318162121 (2024).

10. Hervas, R. *et al.* Cryo-EM structure of a neuronal functional amyloid implicated in memory persistence in *Drosophila*. *Science* **367**, 1230–1234 (2020).

11. Tetter, S. *et al.* TAF15 amyloid filaments in frontotemporal lobar degeneration. *Nature* **625**, 345–351 (2024).

12. Kajava, A. V., Baxa, U., Wickner, R. B. & Steven, A. C. A model for Ure2p prion filaments and other amyloids: The parallel superpleated β-structure. *Proc. Natl. Acad. Sci. U.S.A.* **101**, 7885–7890 (2004).

13. Tanaka, M. *et al.* How Sup35 monomer conformation and amyloid fibril polymorphism determine yeast strain phenotypes. Preprint at https://doi.org/10.21203/rs.3.rs-7945345/v1 (2025).
