# Supplementary material for: Cryo-EM structure of ex vivo Sup35 yeast prion and the factors defining prion phenotype
Source: Nat Commun. 2026 Sep 19;17:10978. doi: 10.1038/s41467-026-77823-3 (PMC13683386; doi:10.1038/s41467-026-77823-3)
Supplement: Supplementary file 2 [file 41467_2026_77823_MOESM2_ESM.pdf]

nature portfolio

Corresponding author(s): Vitaly V. Kushnirov, Alexander A. Dergalev and Konstantin M. Boyko

Last updated by author(s): 14.01.2026

Reporting Summary

Nature Portfolio wishes to improve the reproducibility of the work that we publish. This form provides structure for consistency and transparency in reporting. For further information on Nature Portfolio policies, see our Editorial Policies and the Editorial Policy Checklist.

Statistics

For all statistical analyses, confirm that the following items are present in the figure legend, table legend, main text, or Methods section.

n/a	Confirmed	
☐	☒	The exact sample size (*n*) for each experimental group/condition, given as a discrete number and unit of measurement
☐	☒	A statement on whether measurements were taken from distinct samples or whether the same sample was measured repeatedly
☐	☒	The statistical test(s) used AND whether they are one- or two-sided<br>*Only common tests should be described solely by name; describe more complex techniques in the Methods section.*
☒	☐	A description of all covariates tested
☒	☐	A description of any assumptions or corrections, such as tests of normality and adjustment for multiple comparisons
☐	☒	A full description of the statistical parameters including central tendency (e.g. means) or other basic estimates (e.g. regression coefficient) AND variation (e.g. standard deviation) or associated estimates of uncertainty (e.g. confidence intervals)
☐	☒	For null hypothesis testing, the test statistic (e.g. *F*, *t*, *r*) with confidence intervals, effect sizes, degrees of freedom and *P* value noted<br>*Give P values as exact values whenever suitable.*
☒	☐	For Bayesian analysis, information on the choice of priors and Markov chain Monte Carlo settings
☒	☐	For hierarchical and complex designs, identification of the appropriate level for tests and full reporting of outcomes
☒	☐	Estimates of effect sizes (e.g. Cohen's *d*, Pearson's *r*), indicating how they were calculated

Our web collection on statistics for biologists contains articles on many of the points above.

Software and code

Policy information about availability of computer code

Data collection	SerialEM v4.055; FusionCapt Advance SL4 16.04
Data analysis	Warp v1.0.9, crYOLO v1.9; CryoSPARC v4.6.0; RELION v5.0.0; Coot v1.1.18; PHENIX v1.21.1; CCP4 v9.0; UCSF Chimera v1.13.1; UCSF ChimeraX v1.3rc202110252002; flexAnalysis v3.3.; Microsoft Excel 2016; GraphPad Prism 8; (Fiji Is Just) ImageJ 2.3/1.53q; YASARA v25.9.17; FoldX 5.1 plugin for YASARA; Amyloid Illustrator package v3

For manuscripts utilizing custom algorithms or software that are central to the research but not yet described in published literature, software must be made available to editors and reviewers. We strongly encourage code deposition in a community repository (e.g. GitHub). See the Nature Portfolio guidelines for submitting code & software for further information.

Data

Policy information about availability of data

All manuscripts must include a data availability statement. This statement should provide the following information, where applicable:

- Accession codes, unique identifiers, or web links for publicly available datasets
- A description of any restrictions on data availability
- For clinical datasets or third party data, please ensure that the statement adheres to our policy

Atomic coordinates of [PSI+]-S7 structure have been deposited in the Protein Data Bank under accession number 21BQ. The corresponding cryo-EM density map has been deposited in the Electron Microscopy Data Bank under accession code EMD-67558.

Research involving human participants, their data, or biological material

Policy information about studies with human participants or human data. See also policy information about sex, gender (identity/presentation), and sexual orientation and race, ethnicity and racism.

Reporting on sex and gender	N/A
Reporting on race, ethnicity, or other socially relevant groupings	N/A
Population characteristics	N/A
Recruitment	N/A
Ethics oversight	N/A

Note that full information on the approval of the study protocol must also be provided in the manuscript.

Field-specific reporting

Please select the one below that is the best fit for your research. If you are not sure, read the appropriate sections before making your selection.

[x] Life sciences [ ] Behavioural & social sciences [ ] Ecological, evolutionary & environmental sciences

For a reference copy of the document with all sections, see nature.com/documents/nr-reporting-summary-flat.pdf

Life sciences study design

All studies must disclose on these points even when the disclosure is negative.

Sample size	Sample size was not predetermined. The aim was to collect more than 5,000 cryo-EM movies containing fibrils, from which several hundred thousand helical segments could be selected by automated procedures. Data collection exceeded this target, resulting in approximately 9,000 movies containing fibrils and enabling a near-atomic, isotropic reconstruction suitable for atomic model building and refinement.
Data exclusions	During cryo-EM data processing, a substantial fraction of automatically extracted segments was excluded owing to insufficient image quality or failure to align reliably with the consensus dataset. Such segments typically originated from regions of micrographs affected by local ice thickness variations, contamination, filament overlaps, broken filaments, or structural heterogeneity, which cannot be fully avoided during automated filament picking. These low-quality segments were objectively identified during iterative rounds of 2D classification, 3D classification, and refinement as classes lacking well-defined structural features and were removed based on computational criteria. No data were excluded on the basis of expected structural outcomes or biological interpretation.
Replication	This study reports the cryo-EM structure of a single, homogeneous [PSI+]-S7 prion fibril polymorph obtained from one fibril preparation. Structural reproducibility was evaluated computationally through multiple rounds of independent classification, refinement, and gold-standard FSC analysis, which consistently converged to the same overall fold and helical parameters.<br>The reproducibility of the fibril ex vivo purification protocol was confirmed by replicating the protocol more than 10 times (without the trypsin treatment stage for isolated fibrils) and at least 5 times (including the trypsin treatment stage and additional step of purification from trypsin digestion products).
Randomization	Randomization was not applicable, because group allocation was not a part of the experimental design
Blinding	Blinding was not applicable, because group allocation was not a part of the experimental design. Cryo-EM image processing, classification, and model refinement were performed using automated computational procedures and standard validation criteria.

Reporting for specific materials, systems and methods

We require information from authors about some types of materials, experimental systems and methods used in many studies. Here, indicate whether each material, system or method listed is relevant to your study. If you are not sure if a list item applies to your research, read the appropriate section before selecting a response.

Materials & experimental systems

n/a	Involved in the study
☐	☒ Antibodies
☐	☒ Eukaryotic cell lines
☒	☐ Palaeontology and archaeology
☒	☐ Animals and other organisms
☒	☐ Clinical data
☒	☐ Dual use research of concern
☒	☐ Plants

Methods

n/a	Involved in the study
☒	☐ ChIP-seq
☒	☐ Flow cytometry
☒	☐ MRI-based neuroimaging

Antibodies

Antibodies used	Anti-Sup35NM primary (rabbit, polyclonal; custom); anti-Hsp104 primary (rabbit, polyclonal; ThermoFisher, PA1-024); anti-β-tubulin primary (rabbit, polyclonal; ThermoFisher, PA5-16863); HRP-conjugated secondary IgG (goat, anti-rabbit; Pierce, #31460)
Validation	The anti-Sup35NM antibodies were produced in the M.D. Ter-Avanesyan lab by immunizing a rabbit with the purified recombinant Sup35NM protein, and then affinity-purified. To validate the absence of cross-reactivity with any other proteins in yeast lysate, the lysates of Sup35-WT and Sup35ΔNM yeast strains were subjected to SDS-PAGE followed by Western Blotting, and then stained with anti-Sup35NM primary antibodies and anti-rabbit HRP-conjugated secondary antibodies. No bands were observed in the Sup35ΔNM lysate. Anti-Sup35NM antibodies obtained in this manner were used in Kryndushkin et al. (2003, 10.1074/jbc.M307996200), as well as in many others published studies.

Eukaryotic cell lines

Policy information about cell lines and Sex and Gender in Research

Cell line source(s)	S. cerevisiae yeast strain (74-D694) carrying strong prion variant ([PSI+]-S7) was kindly gifted by Y.O. Chernoff (Georgia Institute of Technology, Atlanta, USA); the weak prion variant ([PSI+]-W2) was obtained de novo in the 74-D694 [psi-] [PIN+] yeast strain (also gifted by Y.O. Chernoff), as described in Degalev et al., 2019 (10.3390/ijms20112633); afterwards, RNQ1 gene was deleted from both [PSI+]-S7 and [PSI+]-W2 cell lines.
Authentication	[PSI+]-S7 and [PSI+]-W2 prion variants were authentified by colony color phenotype (ade1-14 colony color phenotype system), polymer size (SDD-AGE analysis), propagon number analysis and the prion loss upon short-term heat shock. Both prion variants were authentified earlier (Dergalev et al., 2019) by PK mapping of isolated prion polymers, nonsense codone readthrough and SUP35/HSP104 overproduction test.
Mycoplasma contamination	The yeast cell lines were not tested for mycoplasma contamination, because mycoplasma is not a common contaminant of yeast cell cultures
Commonly misidentified lines (See ICLAC register)	No misidentified lines were used

Plants

Seed stocks	N/A
Novel plant genotypes	N/A
Authentication	N/A
