# Supplementary material for: Cryo-EM structure of ex vivo Sup35 yeast prion and the factors defining prion phenotype
Source: Nat Commun. 2026 Sep 19;17:10978. doi: 10.1038/s41467-026-77823-3 (PMC13683386; doi:10.1038/s41467-026-77823-3)
Supplement: Supplementary file 3 [file 41467_2026_77823_MOESM3_ESM.pdf]

nature portfolio

Peer Review File

Cryo-EM structure of ex vivo Sup35 yeast prion and the factors defining prion phenotype

Corresponding Author: Dr Vitaly Kushnirov

This file contains all reviewer reports in order by version, followed by all author rebuttals in order by version.

Version 0:

Reviewer comments:

Reviewer #1

(Remarks to the Author)
Review of “Cryo-EM structure of ex vivo Sup35 yeast prion and the factors defining prion phenotype” by Dergalev et al.
The authors have used cryo-EM to determine a structure of amyloid isolated from cells carrying a ‘strong’ variant of [PSI+] a yeast prion of the Sup35 protein. This is a major advance as the first atomic-level structure of ex vivo amyloid for any yeast prion. This work clarifies many issues in the area. The structure is combined with extensive examination of the effects of mutations in the prion domain on prion stability, filament size and amyloid stability, with comparison of the strong (S7) prion variant used for the structure and a weak (W2) variant.

>A>The authors over-expressed the prion domain of Sup35 (Sup35NM) fused to an acidic 15 residue acidic linker (DDDNEDSEEDEDGGP) followed by GFP. The GFP is cleaved, leaving almost all of the acidic linker attached in order to prevent excessive clumping of filaments. It is known from very early work, and confirmed in the present study, that in many cases mutation from uncharged to charged of single Sup35p residues can destabilize the propagation of [PSI+] (the Sup35 amyloid), and this was explained long ago by the finding that the Sup35p infectious amyloid had a folded parallel in-register structure, so charges would repel eachother. The eleven negative charges in the linker could well have some such effect. The NM-linker-GFP fusion protein is must be templated by the full length Sup35 amyloid in the cells. Can the fusion protein propagate the prion (the S7 variant of [PSI+] studied here) in the absence of the w.t. full-length Sup35p? This could be done with the Sup35 C-terminal (functional) domain expressed separately from the fusion protein. If it does support [PSI+] propagation, that would support the notion that the structure of the fusion protein is at least very close to that of the actual prion. If not, then the results here would have to be re-interpreted and would be of less interest.
>B>Lines 16-18: “The yeast Sup35 prion has multiple structural variants divided into two groups, “strong” and “weak”, differing in the frequency of fibril fragmentation and resulting nonsense-suppressor phenotype.”
:::The authors emphasis on the “strong” vs “weak” division of [PSI+] prion variants is misleading in that 1) there are many other ways of dividing prion variants that do not respect the “strong-weak” division, and 2) there are many gradations of strong vs weak, and 3) two equally strong variants need not be the same structure. The authors assume that S7 has “the” strong [PSI+] structure, but it actually has “a” strong [PSI+] structure. The authors have made an important contribution by determining “a” ex vivo [PSI+] structure, and they need not make this overinterpretation.

>C>Lines 27 – 29: “Comparison of ex vivo Sup35 prion structure with that of Sup35 amyloids formed in vitro shows that the latter differ significantly from the prion structure propagating in yeast cells.’
:::There are many strong and many weak variants of [PSI+]. Amyloid formed in vitro of Sup35p is known to be very infectious for yeast, producing a variety of distinguishable infected clones. It is very possible (even likely) that the structure determined for the in vitro generated amyloid is one of many strong [PSI+] variants capable of in vivo propagation (or a mixture of such variants). The quoted statement should be at least modified. While the authors of the present work can claim that their structure is known to be that of a propagating prion, they cannot claim (as they do here) that the previously determined structure is not a weighted average of such structures.
>D> In Lines 79 to 97, the authors point out differences between labs in the results of PK resistance of parts of the Sup35p prion domain.
:::This is not a grand paradox but may well reflect differences in PK techniques. Perhaps more important, the deuterium exchange experiments also show a difference in the extent of the structure in the prion domains between Sc4 (amyloids producing strong variants) and Sc37 (amyloids producing weak variants).

>E>Line 117-119: “We compared the mechanical properties of Sup35 prions from [PSI+]-S7 and [PSI+]-W2 cells and their interaction with chaperones. The [PSI+]-S7 prion was less resistant to heating and high-molar urea (Fig. 1D-1E, S1C), and more fragile (Fig. 1F).”
:::Ref. 27 showed that filaments formed at 4C (and produce strong [PSI+] on transfection) are more easily dissolved by heating than those formed a 37C (and produce weak [PSI+] variants). The authors’ experiments confirm and extend the old result in that they have used ex vivo material and use more measures of amyloid stability, but the old result should nevertheless be cited.
In this work, one strong and one weak [PSI+] variant are compared. The authors seem to assume that these variants are typical of all strong or weak variants, respectively. One cannot expect them to do these extensive studies on many of each variant, but they should be more reserved in their conclusions.
>F> Fig. 1G compares the amounts of Hsp104 bound to a strong [PSI+] vs a weak [PSI+]. Since Hsp104 levels are elevated on [PSI+] infection (Genetics 2000 Oct;156(2):559-70. doi: 10.1093/genetics/156.2.559), different prion variants might produce different levels of elevation. The authors should include a measure of total Hsp104 in the strong vs weak [PSI+] cells to control for this effect. They almost certainly have this data.
>G>Fig. 2B: The authors find (Fig.2B) that cells expressing the Sup35 G58D mutant protein do not substantially lose either their strong S7 variant or the weak W2 [PSI+]. It looks like there is not even a real color change. The statistically real increased instability of S7 in G58D cells (Fig. 2H, not the Fig. 3H cited by mistake on line 172) is not the kind of gross loss in most cells expected. Several labs have confirmed Cox’s original finding that G58D destabilizes [PSI+], but Chernoff showed that prion variants dependent on this mutation can be isolated. Perhaps some host/strain difference or a prion variant change can explain this and differences in PK results compared to other groups?
Others have shown effects of residues well beyond Core 1A or 1B, even some in M, on the transmission of some strong [PSI+] variants from w.t. (that is lab wild type). These are residues that differ between lab strains and some other wild S. cerevisiae. Transmission barriers of ~95% in some cases were observed.
>H>The phrase, “parallel β-sheet” vs “anti-parallel β-sheet” in amyloid structure studies usually refers to the relative orientation of the β-strands in the plane formed by the β-bonds, the hydrogen bonds between the main chain α-amino group H and α-carboxy O. The authors use the expression “antiparallel β-strands” on Line 255 and perhaps elsewhere to refer to the relative orientation of β-strands in the plane perpendicular to the plane of the β-sheets (and perpendicular to the long axis of the filaments). I think this may be confusing to many readers.
>I> The authors mention the Sup35 peptide GNNQQNY and its role in the studies leading to coining the phrase “steric zipper”. It would be of interest then to know if that sequence in the present context as part of Sup35NM assumed the coformation described for the isolated peptide in the earlier study. Specifically, is it paired, in the “steric zipper” plane, with a sequence having the same relation as in the crystals of GNNQQNY? I believe that in the earlier study the GNNQQNY formed a parallel beta sheet in the plane of the H-bonds of the main peptide chain, and was paired with itself in an anti-parallel way in the “steric zipper” plane. Such self-complementary relations were proposed to be the basis of prion/amyloid formation. Does this structure bear that out? Judging by Fig. 3D, I think that it does not, but I am not sure I am evaluating that correctly.

>J> Line 519: “... 0.4 mg/ml adenine (2% of standard concentration).”
20 mg/ml of adenine cannot be standard concentration. This must be a typo.

>K> The Sc4 structures are not necessarily wrong in any sense. The Sc4 structures include a variety of strong [PSI+] structures, not just the one that is studied in this work, S7. The authors report that even the S7 filaments are heterogeneous in that half have a twisted appearance and half do not. Only the twisted ones could be analyzed. The Sc4 structures reflect what is common among the majority of filaments formed under those conditions. The present structure reflects a single variant, but (unavoidably) only half of the filaments of that variant.
:::Most [PSI+] variants capable of propagating are lethal or nearly lethal and S7 is quite mild by comparison. But the lethal [PSI+] amyloids are probably included in Sc4 amyloid. The lethal [PSI+] amyloids are prions that propagate well (without killing the cells) as long as the function of Sup35p is supplied by molecules that are incapable of joining the filaments. There may be [PSI+] variants that kill the cells independent of inadequate Sup35p function, so this is a minimum estimate of the fraction of lethal amyloid filaments.
:::Even a single strong [PSI+] strain really consists of a “cloud” of prion variants which segregate on non-selective growth and mutate between eachother. These are variants with differing sensitivity to the “intra-species barriers” due to minor amino acid sequence differences among wild strains of S. cerevisiae in the N and M regions of Sup35p. There are also strong [PSI+] that are relatively insensitive to the yeast anti-prion systems although a majority are sensitive.

Reviewer #2

(Remarks to the Author)
In this manuscript, Dergalev et al. study the structural differences between [PSI+] prions that give strong nonsense suppression phenotypes and those that give weak nonsense suppression phenotypes. They use over-expression of the priononogenic residues 1-239 of Sup35 fused to GFP in cells harbouring [PSI+]-S7 and [PSI+]-W2 prions, as models for strong and weak prions, respectively.

The authors first confirmed that [PSI+]-S7 prions were less stable than [PSI+]-W2 prions, and that they indeed resulted in a stronger nonsense suppression phenotype. Furthermore, they showed that the two prions had a similar amount of Hsp104 binding, in line with previous work on HSP70 binding. These results support the physical/structural basis for strong/weak nonsense suppression, rather than extent of chaperone binding.

Next, the authors analysed the effect of several previously-reported prion-destabilizing amino acid substitutions in the

Core1A and Core1B regions of Sup35 on the nonsense suppression phenotype. As previously suggested, substitutions in Core1B reduced the strong [PSI+]-S7 prion phenotype, but had little effect on [PSI+]-W2 phenotype, whereas substitutions in Core1A reduced the phenotypes of both prions. This suggests that Core1A is important for strong and weak prions, but that Core1B is only important for strong prions.

The authors then determined the structure of the [PSI+]-S7 prions using cryo-EM, which showed that the Core1B region packed against β-arch formed by the Core1A region. These substructures were stabilized by zipper packing of the abundant glutamine and asparagine residues.

Using this structure, the authors computationally calculated the thermodynamic properties free energy of unfolding and solvation energy to assess its stability, and compared these to calculations for published amyloid structures. In doing so, they present evidence that the solvation energy is a better metric to assess stability than the more commonly used free energy of unfolding, the latter of which is biased toward hydrophobic interactions. This is a useful comparison for the amyloid field. The authors also used these thermodynamic metrics to assess the effects of uncharged-to-charged amino acid substitutions. This identified previously-reported prion-destabilizing substitutions, further validating the use of this approach.

Finally, the authors compared their PSI+]-S7 prion structure to the known structures of amyloids formed in vitro from the priononogenic residues of Sup35 at 4°C and 37°C (Sc4 and Sc37, respectively). Sc4 shares a similar Core1A β-arch fold, but lacks Core1B packing. In contrast, Sc37 lacks the Core1A β-arch fold, but shows similar packing of Core1B against Core1A. As such, the computed thermodynamic properties of [PSI+]-S7 were more similar to Sc37 than Sc4. This is consistent with previous work showing that strong [PSI+] prions have similar proteinase K digestion patterns to Sc37, while weak [PSI+] prions are more similar to Sc4. This is surprising, because Sc4 results in strong nonsense suppression in cells, whereas Sc37 results in weak.

This apparent paradox is addressed in the discussion, where the authors suggest that rare filament types in these in vitro preparations are selected for and amplified in cells and that these confer the strong or weak phenotypes (not the determined Sc4 and Sc37 structures).

The experiments have been conducted to a high standard and the methods a documented in detail. The finding that ex vivo prion structures differ from in vitro prion structures is important for the field to consider going forward. The use of computed solvation energy instead of free energy of unfolding is also a useful approach for the field.

Major comments:
1. It is not clear how the ex vivo prions studied here by over-expressing the priononogenic residues 1-239 of Sup35 fused to GFP relate to native [PSI+] prions. The authors state that they "adopt the pre-existing prion fold," but evidence is not provided. The authors state that native [PSI+] prion fibrils are too short for cryo-EM, but the data is not shown. Could the authors add data, such as 2D class averages, to support their claim that the prions they are studying are similar to native prions? In the absence of such data, a passage should be added to the discussion to acknowledge that the structure of native [PSI+] prions might differ.

2. Are the authors sure that the use of 4% SDS during the extraction of prions does not alter their structure or disassemble a proportion of the fibrils? The authors say they monitored this process using fluorescence microscopy, but I could not find the associated figure.

3. The authors state that the [PSI+]-W2 prion fibrils were essentially untwisted, precluding structure determination by cryo-EM. Could the authors show this data? Are the 2D class averages distinct from those of untwisted [PSI+]-S7 prion fibrils?

4. The authors' hypothesis that rare fibril types in Sc4 and Sc37 may be selected for in cells could be tested using the approach they take here (i.e. extract ex vivo prions from cells with Sc4 and Sc37. This may be beyond the scope of this study, but could be discussed in the text.

Minor comments:
1. In Fig. 1A-1C and S1A, molecular mass markers are missing for SDS-AGE and the n numbers are not reported. It would be helpful to add quantification of repeated experiments.

2. Fig S1B is not cited in order.

3. Fig 2D is cited erroneously for Fig 1G.

4. Core1, Core1A and Core 1B are not defined in the Results text.

5. In Fig S4C it is not possible to evaluate the twist of the fibrils due to the particle coordinates. Could images with and without cooridinates be shown?

6. The conclusions that, "These segments displayed similar 2D class features apart from the degree of twist, indicating a common molecular fold", could be further tested by producing untwisted projections of the [PSI+]-S7 structure, as performed in https://www.nature.com/articles/s41586-022-05319-3.

7. Are the authors sure that their fibril structure has a left-handed twist? This could be confirmed by examining densities for

peptide group oxygens, which should be visible in their high-resolution reconstruction, as performed here, https://www.nature.com/articles/s41586-021-04199-3.

8. The statement "it appears likely that only single-filament fibrils could succeed as viable prions in yeasts" is not evident from the evidence, do the authors have supporting evidence?

Version 1:

Reviewer comments:

Reviewer #1

(Remarks to the Author)
The authors have appropriately responded to my comments.

Reviewer #2

(Remarks to the Author)
The authors have addressed all of my comments in the revised manuscript through the addition of new data and text. The comparison of twisted and untwisted 2D class averages is particuarly comprehensive, as is the text comparing [PSI+]-S7 fibrils to native [PSI+] prions.

Open Access This Peer Review File is licensed under a Creative Commons Attribution 4.0 International License, which permits use, sharing, adaptation, distribution and reproduction in any medium or format, as long as you give appropriate credit to the original author(s) and the source, provide a link to the Creative Commons license, and indicate if changes were made.
In cases where reviewers are anonymous, credit should be given to 'Anonymous Referee' and the source.
The images or other third party material in this Peer Review File are included in the article's Creative Commons license, unless indicated otherwise in a credit line to the material. If material is not included in the article's Creative Commons license and your intended use is not permitted by statutory regulation or exceeds the permitted use, you will need to obtain permission directly from the copyright holder.
To view a copy of this license, visit https://creativecommons.org/licenses/by/4.0/

Dear Reviewer,
Thank you for thorough analysis of our work that helped to improve the manuscript.
Please see below our answers. All citations are made as hyperlinks (press Cntrl+click).

Reviewer #1 (Remarks to the Author):

Review of "Cryo-EM structure of ex vivo Sup35 yeast prion and the factors defining prion phenotype" by Dergalev et al.
The authors have used cryo-EM to determine a structure of amyloid isolated from cells carrying a 'strong' variant of [PSI+] a yeast prion of the Sup35 protein. This is a major advance as the first atomic-level structure of ex vivo amyloid for any yeast prion. This work clarifies many issues in the area. The structure is combined with extensive examination of the effects of mutations in the prion domain on prion stability, filament size and amyloid stability, with comparison of the strong (S7) prion variant used for the structure and a weak (W2) variant.

>A>The authors over-expressed the prion domain of Sup35 (Sup35NM) fused to an acidic 15 residue acidic linker (DDDNEDSEEDEDGGP) followed by GFP. The GFP is cleaved, leaving almost all of the acidic linker attached in order to prevent excessive clumping of filaments. It is known from very early work, and confirmed in the present study, that in many cases mutation from uncharged to charged of single Sup35p residues can destabilize the propagation of [PSI+] (the Sup35 amyloid), and this was explained long ago by the finding that the Sup35p infectious amyloid had a folded parallel in-register structure, so charges would repel each other. The eleven negative charges in the linker could well have some such effect. The NM-linker-GFP fusion protein must be templated by the full length Sup35 amyloid in the cells. Can the fusion protein propagate the prion (the S7 variant of [PSI+] studied here) in the absence of the w.t. full-length Sup35p? This could be done with the Sup35 C-terminal (functional) domain expressed separately from the fusion protein. If it does support [PSI+] propagation, that would support the notion that the structure of the fusion protein is at least very close to that of the actual prion. If not, then the results here would have to be re-interpreted and would be of less interest.

Authors: We have the data indicating that the charged insertion did not affect the [*PSI*+]-S7 prion structure. Firstly, the supplementary **Figure S5B** (possibly not noticed by the Reviewer) shows that the spheroplast transformation of [*psi*-] yeast with the fibrils used for [*PSI*+]-S7 structure reconstruction produced only strong [*PSI*+]. This suggests that the original strong [*PSI*+] fold was mainly preserved. Secondly, any possible effect of the charged insert is most likely to occur in the Core 1B, which is closest to the insert. We can monitor alterations in the Core 1B structure with proteinase K (PK). We compared spectra of the peptides of the proteinase K digestion of the standard Sup35NM-GFP and Sup35DE-GFP proteins overproduced in the [*PSI*+]-S7 cells and found them highly similar (**Figure S5E**). In particular, strong peaks of the largest peptides 2-70,2-71,2-72 indicate the lack of unfolding or weakening of the Core 1B, while the group of smaller peptides, all ending in Core 1B suggest the lack of Core 1A alterations.

Following the Reviewer's advice, we performed two genetic experiments showing that the Sup35 derivative with the acidic cluster is still capable of the [*PSI*+]-S7 prion propagation in vivo. In the first of them, we moved the *SUP35-DE-GFP* gene to a centromeric plasmid with the *SUP35* promoter and the C domain instead of GFP. The *SUP35* gene was changed for *SUP35-DE* through plasmid shuffle. After this, the [*PSI*+]-S7 prion continued to propagate, although with slightly weakened suppressor phenotype. We should note that the Sup35-DE construct lacked the Sup35 residues 124-155, which includes half of the Ssa1/2 binding site that is important for prion propagation. So, we relate the reduced stability to truncation of the Ssa1/2 site (residues 143-164, Shen et al., 2024)) rather than to the DE cluster. After the reverse Sup35DE → Sup35WT shuffle, the strong suppressor phenotype was restored (**Figure S5D**). In the second experiment (**Figure S5C**), we overproduced in the [*PSI*+]-S7 cells the Sup35DE-GFP construct in the same way as for the cryo-EM sample preparation, but for much

longer time, ~10 cell generations vs one. In this setup, overproduced Sup35DE-GFP construct formed mixed fibrils with pre-existing Sup35-WT prion polymers composed mainly (~100:1) of Sup35DE-GFP. Then overproduction was stopped, the cells were allowed to form colonies (~25 generations) and they all showed a strong phenotype. In sum, these results indicate that the Sup35DE protein with acidic cluster is able to propagate the prion state.

>B>Lines 16-18: "The yeast Sup35 prion has multiple structural variants divided into two groups, "strong" and "weak", differing in the frequency of fibril fragmentation and resulting nonsense-suppressor phenotype."

:::The authors emphasis on the "strong" vs "weak" division of [PSI+] prion variants is misleading in that 1) there are many other ways of dividing prion variants that do not respect the "strong-weak" division, and 2) there are many gradations of strong vs weak, and 3) two equally strong variants need not be the same structure. The authors assume that S7 has "the" strong [PSI+] structure, but it actually has "a" strong [PSI+] structure. The authors have made an important contribution by determining "a" ex vivo [PSI+] structure, and they need not make this overinterpretation.

Authors: We agree that S7 may represent just one of strong [*PSI*+] variants and introduced proper corrections and explanations to the text, lines 71-75. Other places in the text now imply the existence of many strong [*PSI*+] variants.

However, we cannot agree that the division for weak and strong is misleading. Because (1) such division does not preclude the possibility to divide [*PSI*+] variants by any other phenotypic "dimensions". Many of such phenotypes were described by Reed Wickner, some by us and other authors. (2) Previously, we have made a large number of PK digests of [*PSI*+] isolates, 32 published (Dergalev et al, 2019; Alieva et al., 2023) and many unpublished. These were obtained in various ways (with/without Sup35 overproduction, with non-Sup35 inducer, during coldroom storage and even lethals described below, under >K>). We observed that they all clearly fall into two groups that can be reliably distinguished by the three criteria: their PK digestion pattern (in all weak variants the peak 2-42 is higher than 2-35 and 2-38, peaks 2-70,71,72 are minor, strong [*PSI*+]: opposite relations), and reaction to overproduction of Sup35 (lethal vs tolerant) and Hsp104 (sensitive or resistant). (3) many publications in the field used the division for weak and strong and observed various different properties of these two types. Hopefully, these arguments allow presuming that strong and weak [*PSI*+] represent two major groups of [*PSI*+] variants and use of the studied variants S7 and W2 as representatives of these groups.

To complete the picture, let us note that according to (Huang and King, 2020) only one strong and four weak [*PSI*+] variants can be obtained in a standard way, by Sup35 overproduction. (18 more variants were obtained by passing standard [*PSI*+] on Sup35 mutations with anti-prion properties located within Core 1). We allow that more than one strong [*PSI*+] variant is possible among the standard group, but basing on our PK experience their structural variation should be minor. Also, we apply the division for strong and weak [*PSI+*] only to the variants obtained in the "standard" way.

We tested some non-"standard" [*PSI*+] variants and found that spectra of their PK-resistant peptides showed some peptides and proportions not found in "standard" [*PSI*+], though the majority of peptides were typical of standard [*PSI*+]. These variants include the exotic strong W8 variant obtained by CY King (Huang et al., 2015) and three unique [*PSI*+] variants propagating in the presence of weakened Hsp104-T160M (Huang et al., 2021).

>C>Lines 27 – 29: "Comparison of ex vivo Sup35 prion structure with that of Sup35 amyloids formed in vitro shows that the latter differ significantly from the prion structure propagating in yeast cells.'

:::There are many strong and many weak variants of [PSI+]. Amyloid formed in vitro of Sup35p is known to be very infectious for yeast, producing a variety of distinguishable infected clones. It

is very possible (even likely) that the structure determined for the in vitro generated amyloid is one of many strong [PSI+] variants capable of in vivo propagation (or a mixture of such variants). The quoted statement should be at least modified. While the authors of the present work can claim that their structure is known to be that of a propagating prion, they cannot claim (as they do here) that the previously determined structure is not a weighted average of such structures.

Authors: Alterations were introduced throughout the text that imply the existence of many strong and weak [*PSI*+] variants.

Based on our knowledge of the cryo-EM reconstruction workflow, the Sc4 structure is not a weighted average of multiple polymorphs. More detailed explanation about interpretation of cryo-EM data is in the >K> answer.

There is the following evidence in favor of the Sc4 inability to propagate in yeast, though it would be difficult to prove it completely. (1) C-Y King tested the [*PSI*+] variant obtained by M. Tanaka after transformation with Sc4 and defined it as the VH variant, which, as we have shown, has a typical strong PK digestion pattern. In the same way, Sc37 yielded VK, a weak variant showing typically weak PK pattern (Huang and King, 2020; Dergalev et al, 2019) (2) One of the important achievements of our work and that of Tanaka et al., 2025 is that they establish a good fit between the precise atomic structure of an amyloid core and the PK digestion results. The Sc4 PK digestion pattern fits very well to its structure and this pattern is clearly different from PK patterns of all strong [*PSI*+] variants studied by us. Thus, the Sc4 prion fold must be, at least, very rare in vivo. (3) M. Tanaka's lab observed that Sc4 fibrils were completely disassembled in vitro by Hsp104/Ssa1/Sis1 complex, in contrast to Sc37 amyloid, which resisted such treatment, being able to support a balance between disassembly and polymerization (Nakagawa et al., 2022).

We explain the high infectivity of Sc4 by the ease of structural rearrangement of the Region 1B in yeast cells to a like of [*PSI*+]-S7 structure (as shown in **Figure 6A**), which should not be difficult, providing that the Region 1A is folded similarly in Sc4 and [*PSI*+]-S7.

>D> In Lines 79 to 97, the authors point out differences between labs in the results of PK resistance of parts of the Sup35p prion domain.
:::This is not a grand paradox but may well reflect differences in PK techniques. Perhaps more important, the deuterium exchange experiments also show a difference in the extent of the structure in the prion domains between Sc4 (amyloids producing strong variants) and Sc37 (amyloids producing weak variants).

Authors: We consider it unlikely that the difference in the PK patterns between Sc4 and [*PSI*+]-S7 can be explained by any differences in PK techniques. Firstly, because these PK patterns (Ohhashi et al., 2018, Dergalev et al, 2019) correspond very well to the respective atomic structures obtained by us and Tanaka et al., 2025. Prion structures include residues 2 to 64/65 in S7 and Sc37 and this is reflected as major 2-70/2-71/2-72 peaks in respective PK digests. In contrast, residues 32-46 and 53-64 in Sc4 are unstructured and this corresponds to the absence of the 2-70/2-71/2-72 peaks in Sc4 PK digest. (Interestingly and importantly, PK-protected region was larger by about 8 residues than the structured region in S7 as well as in Sc37 – this establishes a relation between PK digests and actual structures and suggests something significant about the mechanism of PK action). Secondly, our extensive experience with PK digestion allows concluding that it is not sensitive to reaction conditions, such as the presence of detergents, sucrose, calcium, or reasonable temperature variations. There is some dependence on concentrations of PK and amyloid, but we performed digests at different concentrations and the PK patterns were never similar to those of Tanaka.

Finally, we generated amyloid in vitro at 4˚C by the method of Tanaka et al., 2004, and its PK digest was similar to Sc4, not to S7. The spectrum quality (below) is not as good as in our other spectra, but the key peaks are evident and similar to Ohhashi et al., 2018: 2-38, 2-42, 2-45 and 2-46, and no signal for the peptides 2-70, 2-71, 2-72. Two additional peaks that certainly do not belong to

the Core1: 76-132 and unidentified. We relate the difference to the preparation, rather than to the PK procedure.

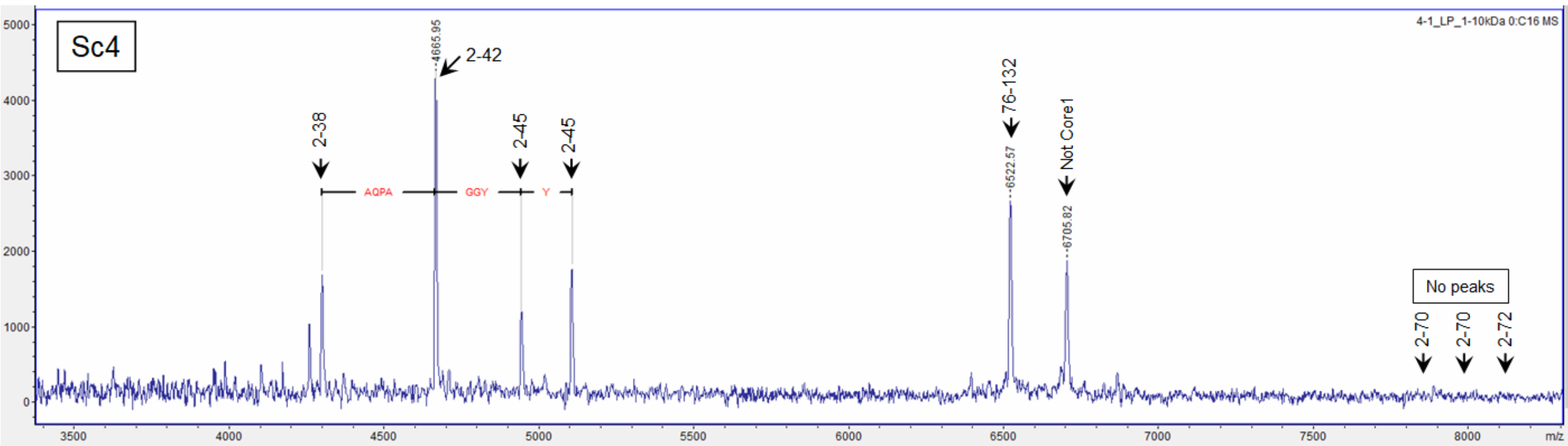

The deuterium exchange data: we trust them, but regrettably, there are no such data for ex-vivo prions.

>E>Line 117-119: "We compared the mechanical properties of Sup35 prions from [PSI+]-S7 and [PSI+]-W2 cells and their interaction with chaperones. The [PSI+]-S7 prion was less resistant to heating and high-molar urea (Fig. 1D-1E, S1C), and more fragile (Fig. 1F)."
:::Ref. 27 showed that filaments formed at 4C (and produce strong [PSI+] on transfection) are more easily dissolved by heating than those formed a 37C (and produce weak [PSI+] variants). The authors' experiments confirm and extend the old result in that they have used ex vivo material and use more measures of amyloid stability, but the old result should nevertheless be cited.
In this work, one strong and one weak [PSI+] variant are compared. The authors seem to assume that these variants are typical of all strong or weak variants, respectively. One cannot expect them to do these extensive studies on many of each variant, but they should be more reserved in their conclusions.

Authors: The thermal resistance (prion melting) tests were made several times previously by different labs with different [*PSI*+] variants, and in all cases ex vivo weak [*PSI*+] variants were more stable than strong ones, the same as Sc37/Sc30 fibrils were more stable than Sc4 (Alexandrov et al., 2012; Huang et al., 2021; Tanaka et al., 2004; Ohhashi et al., 2018). Some weak [*PSI*+] isolates were tested for thermal stability by us, but never published. All references were added to the text, lines 125-127.

We have added an explanation to the manuscript about whether our findings are applicable to other strong and weak [*PSI*+] variants (lines 71-75).

>F> Fig. 1G compares the amounts of Hsp104 bound to a strong [PSI+] vs a weak [PSI+]. Since Hsp104 levels are elevated on [PSI+] infection (Genetics 2000 Oct;156(2):559-70. doi: 10.1093/genetics/156.2.559), different prion variants might produce different levels of elevation. The authors should include a measure of total Hsp104 in the strong vs weak [PSI+] cells to control for this effect. They almost certainly have this data.

Authors: We have added the data comparing the normalized amounts of Hsp104 in the [*PSI*+]-S7 and [*PSI*+]-W2 cell lysates (**Figure S1D**). These data indicate that Hsp104 production levels in the [*PSI*+]-S7 and [*PSI*+]-W2 strains do not show any significant difference (p = 0.43).

>G>Fig. 2B: The authors find (Fig.2B) that cells expressing the Sup35 G58D mutant protein do not substantially lose either their strong S7 variant or the weak W2 [PSI+]. It looks like there is not even a real color change. The statistically real increased instability of S7 in G58D cells (Fig. 2H, not the Fig. 3H cited by mistake on line 172) is not the kind of gross loss in most cells expected. Several labs have confirmed Cox's original finding that G58D destabilizes [PSI+], but

Chernoff showed that prion variants dependent on this mutation can be isolated. Perhaps some host/strain difference or a prion variant change can explain this and differences in PK results compared to other groups?
Others have shown effects of residues well beyond Core 1A or 1B, even some in M, on the transmission of some strong [PSI+] variants from w.t. (that is lab wild type). These are residues that differ between lab strains and some other wild S. cerevisiae. Transmission barriers of ~95% in some cases were observed.

Authors: Different efficiency of G58D mutation in different strong [*PSI*+] strains was observed previously by different authors. The effect of G58D on weak [*PSI*+] was always much weaker or missing. These observations make it reasonable to think that the differences depend on strain background, genetic or epigenetic, but beyond Sup35. The modest destabilization effect of G58D mutation could also relate to our conclusion that the 1B part of the Core 1 is less critical than 1A even for strong [*PSI*+].

In contrast, the PK patterns of weak and strong [*PSI*+] variants did not depend on strain background (many [*PSI*+] isolates of the 74-D694 and 5V-H19 strains were tested) (Dergalev et al, 2019). There is just one difference in PK results with other groups – the difference with the lab of Tanaka (Ohhashi et al., 2018). We hope that the established atomic structures of the S7 prion, Sc4 and Sc37 amyloid shows convincingly that this PK difference is not an artifact, but a correct reflection of the actual significant difference of the structures formed in vitro and propagating in vivo.

Regarding antiprion mutations beyond the Core 1, we checked recently the finding of the importance of the Ssa1/2 binding site in the M domain (Nakagawa et al., 2022) and can confirm that it is critical for any [*PSI*+] variant propagation. Mutations destroying the Core 2 (residues ~ 90-120) can potentially affect [*PSI*+], though our data about this are contradictory.

The Fig. 3 citation was corrected.

>H>The phrase, “parallel β-sheet” vs “anti-parallel β-sheet” in amyloid structure studies usually refers to the relative orientation of the β-strands in the plane formed by the β-bonds, the hydrogen bonds between the main chain α-amino group H and α-carboxy O. The authors use the expression “antiparallel β-strands” on Line 255 and perhaps elsewhere to refer to the relative orientation of β-strands in the plane perpendicular to the plane of the β-sheets (and perpendicular to the long axis of the filaments). I think this may be confusing to many readers.

Authors: To avoid the confusion, we removed the word “antiparallel” from this phrase. This should not cause misunderstanding because the anti-parallel orientation of these strands can be seen from the structure image.

>I> The authors mention the Sup35 peptide GNNQQNY and its role in the studies leading to coining the phrase “steric zipper”. It would be of interest then to know if that sequence in the present context as part of Sup35NM assumed the coformation described for the isolated peptide in the earlier study. Specifically, is it paired, in the “steric zipper” plane, with a sequence having the same relation as in the crystals of GNNQQNY? I believe that in the earlier study the GNNQQNY formed a parallel beta sheet in the plane of the H-bonds of the main peptide chain, and was paired with itself in an anti-parallel way in the “steric zipper” plane. Such self-complementary relations were proposed to be the basis of prion/amyloid formation. Does this structure bear that out? Judging by Fig. 3D, I think that it does not, but I am not sure I am evaluating that correctly.

Authors: We compared the structure of [*PSI*+]-S7 fibrils with one of the published GNNQQNY structures (PDB ID: 5K2G) in **Figure S9H**. As could be seen from their overlay, five residues NQQNY have almost identical fold, while the first GN residues are folded differently. GNNQQNY forms a steric zipper with the 57-QGGYQQ-62 peptide, which partially mimics the structure of anti-parallel GNNQQNY molecule that forms steric zipper in the microcrystal. Thus, opposite to microcrystals, the

steric zipper in the amyloid fibril structure is formed between the stretches with non-identical sequence, and seem to have somewhat less perfect packing of sidechains, as could be seen from their shape complementarity (SC) parameter (discussed in the Supplementary text, chapter "Lateral stabilization interactions").

>J> Line 519: "... 0.4 mg/ml adenine (2% of standard concentration)."
20 mg/ml of adenine cannot be standard concentration. This must be a typo.
Authors: Our fault, certainly this should be 0.4 mg/l, not /ml. Corrected.

>K> The Sc4 structures are not necessarily wrong in any sense. The Sc4 structures include a variety of strong [PSI+] structures, not just the one that is studied in this work, S7. The authors report that even the S7 filaments are heterogeneous in that half have a twisted appearance and half do not. Only the twisted ones could be analyzed. The Sc4 structures reflect what is common among the majority of filaments formed under those conditions. The present structure reflects a single variant, but (unavoidably) only half of the filaments of that variant.
:::Most [PSI+] variants capable of propagating are lethal or nearly lethal and S7 is quite mild by comparison. But the lethal [PSI+] amyloids are probably included in Sc4 amyloid. The lethal [PSI+] amyloids are prions that propagate well (without killing the cells) as long as the function of Sup35p is supplied by molecules that are incapable of joining the filaments. There may be [PSI+] variants that kill the cells independent of inadequate Sup35p function, so this is a minimum estimate of the fraction of lethal amyloid filaments.

Authors: Based on our knowledge of the cryo-EM reconstruction workflow, the Sc4 structure is not a "superposition" or "weighted average" of multiple polymorphs – instead, it represents the single major structure within the Sc4 preparation. Raw images of fibril segments undergo subsequent 2D and 3D classification procedures, which divide distinct structural polymorphs (if such are present in the dataset) into different groups, and each group is finally used for separate structural reconstruction process, resulting in distinct atomic structures. For example, in the Tanaka et al., 2025 manuscript, the authors reconstructed several distinct polymorphs for each of Sc37, S17R4 and S17R37 preparations. However, using the same workflow, they reported just one structure in Sc4 preparation, suggesting it to be highly homogeneous. This doesn't prove that alternative polymorphs are completely absent in Sc4 dataset, but they are very rare.

The Sc4 PK digestion pattern, namely, the lack of the 2-70/2-71/2-72 peptides, typical of all studied strong [*PSI*+], suggests that Sc4 does not contain any significant proportion of the strong prion conformers found in vivo.

The twisted and untwisted segments of [*PSI*+]-S7 prion often found within the same individual filaments do not represent truly distinct fibril species (**Figure S6A**). Based on comparison of their 2D class averages, and computational analysis suggested by the Reviewer #2, they should have nearly the same structure (**Figure S7D**). The twist variation could be an artifact of sample preparation or fibril's interaction with the cryo-EM grid. Some previous articles studying cryo-EM structures of amyloid fibrils reported a similar phenomenon (for example, Yang et al., 2022).

Regarding lethal [*PSI*+]: we have interesting unpublished observations suggesting that lethal [*PSI*+] could relate not to unusual Sup35 prion folds, but to some epigenetic events outside of Sup35. For our 2019 paper, we obtained [*PSI*+] de novo in the cells with multicopy Sup35NM-GFP under tetracycline promoter and a centromeric rescue plasmid encoding Sup35C and Sup45. Many [*PSI*+] isolates appearing in such background were lethal. Interestingly, about a half of the lethal isolates showed PK digestion spectra typical of weak variants (others showed typical strong spectra but nothing uncommon), typical relatively high levels of soluble Sup35 and produced weak [*PSI*+] after the loss of lethality. We also observed that strong and weak [*PSI*+] frequently acquire lethality in the presence of the rescue plasmid. This suggests that the lethality may be unrelated to Sup35 prion fold, possibly relating to either Sup45 decrease or Sup35-Sup45 interaction.

:::Even a single strong [PSI+] strain really consists of a “cloud” of prion variants which segregate on non-selective growth and mutate between each other. These are variants with differing sensitivity to the “intra-species barriers” due to minor amino acid sequence differences among wild strains of S. cerevisiae in the N and M regions of Sup35p. There are also strong [PSI+] that are relatively insensitive to the yeast anti-prion systems although a majority are sensitive.

Authors: We can agree with this note. However, the rate of interconversion between different prion folds and the proportion between the major and minor prion structures are currently unknown. The cryo-EM approach could provide an answer. With our cryo-EM dataset on the [*PSI*+]-S7 prion, we did not find any minor polymorphs with different amyloid core structure (even though we attempted to find them using 3D Variability Analysis tool of the CryoSparc software). This result does not prove that such heterogeneity is completely absent in our preparation – but indicates that the proportion of alternative structures is too low to be detected.

Dear Reviewer,
Thank you for thorough analysis of our work that helped to improve the manuscript.
Please see below our answers. All citations are made as hyperlinks (press Cntrl+click).

Reviewer #2 (Remarks to the Author):

In this manuscript, Dergalev et al. study the structural differences between [PSI+] prions that give strong nonsense suppression phenotypes and those that give weak nonsense suppression phenotypes. They use over-expression of the priononogenic residues 1-239 of Sup35 fused to GFP in cells harbouring [PSI+]-S7 and [PSI+]-W2 prions, as models for strong and weak prions, respectively.

The authors first confirmed that [PSI+]-S7 prions were less stable than [PSI+]-W2 prions, and that they indeed resulted in a stronger nonsense suppression phenotype. Furthermore, they showed that the two prions had a similar amount of Hsp104 binding, in line with previous work on HSP70 binding. These results support the physical/structural basis for strong/weak nonsense suppression, rather than extent of chaperone binding.

Next, the authors analysed the effect of several previously-reported prion-destabilizing amino acid substitutions in the Core1A and Core1B regions of Sup35 on the nonsense suppression phenotype. As previously suggested, substitutions in Core1B reduced the strong [PSI+]-S7 prion phenotype, but had little effect on [PSI+]-W2 phenotype, whereas substitutions in Core1A reduced the phenotypes of both prions. This suggests that Core1A is important for strong and weak prions, but that Core1B is only important for strong prions.

The authors then determined the structure of the [PSI+]-S7 prions using cryo-EM, which showed that the Core1B region packed against β-arch formed by the Core1A region. These substructures were stabilized by zipper packing of the abundant glutamine and asparagine residues.

Using this structure, the authors computationally calculated the thermodynamic properties free energy of unfolding and solvation energy to assess its stability, and compared these to calculations for published amyloid structures. In doing so, they present evidence that the solvation energy is a better metric to assess stability than the more commonly used free energy of unfolding, the latter of which is biased toward hydrophobic interactions. This is a useful comparison for the amyloid field. The authors also used these thermodynamic metrics to assess the effects of uncharged-to-charged amino acid substitutions. This identified previously-reported prion-destabilizing substitutions, further validating the use of this approach.

Finally, the authors compared their PSI+]-S7 prion structure to the known structures of amyloids formed in vitro from the priononogenic residues of Sup35 at 4℃ and 37℃ (Sc4 and Sc37, respectively). Sc4 shares a similar Core1A β-arch fold, but lacks Core1B packing. In contrast, Sc37 lacks the Core1A β-arch fold, but shows similar packing of Core1B against Core1A. As such, the computed thermodynamic properties of [PSI+]-S7 were more similar to Sc37 than Sc4. This is consistent with previous work showing that strong [PSI+] prions have similar proteinase K digestion patterns to Sc37, while weak [PSI+] prions are more similar to Sc4. This is surprising, because Sc4 results in strong nonsense suppression in cells, whereas Sc37 results in weak.

This apparent paradox is addressed in the discussion, where the authors suggest that rare filament types in these in vitro preparations are selected for and amplified in cells and that these confer the strong or weak phenotypes (not the determined Sc4 and Sc37 structures).

The experiments have been conducted to a high standard and the methods a documented in detail. The finding that ex vivo prion structures differ from in vitro prion structures is important for the field to consider going forward. The use of computed solvation energy instead of free energy of unfolding is also a useful approach for the field.

Major comments:

1. It is not clear how the ex vivo prions studied here by over-expressing the priononogenic residues 1-239 of Sup35 fused to GFP relate to native [PSI+] prions. The authors state that they "adopt the pre-existing prion fold," but evidence is not provided. The authors state that native [PSI+] prion fibrils are too short for cryo-EM, but the data is not shown. Could the authors add data, such as 2D class averages, to support their claim that the prions they are studying are similar to native prions? In the absence of such data, a passage should be added to the discussion to acknowledge that the structure of native [PSI+] prions might differ.

Authors: We did not try to obtain 2D class averages of the fully-native Sup35 prion fibrils. This could be very hard or impossible for the reasons described below. However, we have sufficiently strong evidence suggesting that the overproduced Sup35DE-GFP protein faithfully reproduces the native prion fold:

(1) The [*PSI*+]-S7 fibrils used for cryo-EM were also used to transform yeast cells, and all colonies that obtained prion were of the strong [*PSI*+] phenotype (**Figure S5B**). Thus, the S7 structure that we established certainly belongs to a strong [*PSI*+] variant. In the same way, [*PSI*+]-W2 fibrils used for cryo-EM reproduced the weak variant upon transformation in yeast (**Figure S5B**).

(2) The formation of prion particles by the overproduced Sup35DE-GFP protein occurred in the same cellular environment as that of the native prion, and for a relatively short period of time (overnight, but about one cell generation). Sequence alterations located in this case far outside of the prion core region should not affect prion fold during polymerization. For comparison, Chang et al., 2008 showed that the Sup35(1-61)-GFP protein (i.e. GFP was attached to Core1 lacking three last residues) faithfully reproduces different [*PSI*+] prion folds, being templated in vitro by ex-vivo prions. Such in vitro fibrils were used to transform yeast and the transformants were typed against a set of Sup35 mutants able to distinguish [*PSI*+] variants. So, they used a rougher Sup35 construct, different environment, but still [*PSI*+] prion variant was unchanged.

(3) We have extensive experience with partial proteinase K (PK) digestion of ex-vivo prions coupled with MALDI. PK digests allow us to reliably distinguish strong and weak [*PSI*+] variants (Dergalev et al, 2019) and to detect many finer structural distinctions. In this study we showed that the spectra of PK-resistant peptides are very similar between native [*PSI*+] prion polymers and overproduced Sup35NM-GFP protein templated by native prion. This supports the idea that overproduction and the change of the Sup35 C domain for GFP do not change the native [*PSI*+] prion fold. The Proteinase K digestion pattern of the Sup35DE-GFP fibrils, extracted from [*PSI*+]-S7 cells, is very similar to that obtained with Sup35NM-GFP construct (**Figure S5E**), suggesting the lack of structural alterations caused by the negatively charged DE cluster.

(4) [*PSI*+]-S7 cells growth with Sup35DE-GFP overproduction for 10 generations (i.e. several times longer than for obtaining the studied preparations), did not change their prion phenotype, as evident after stopping overproduction (**Figure S5C**).

We did not try using native S7 prion fibrils for cryo-EM reconstruction for the following reasons:
(1) The difficulty of obtaining native prion preparations with sufficient purity, quantity and fibril length. Natural S7 prion is sufficiently large to cause great problems with chromatographic purification, required in its case. But S7 prion is still insufficiently large for cryo-EM reconstruction, and overproduction allowed obtaining longer fibrils. The small length of the native [*PSI*+] fibrils was confirmed in different ways. We estimated the length of strong [*PSI*+] fibrils by SDD-AGE

electrophoresis as 8-20 protomers, or <10nm (Kryndushkin et al., 2003). Such length is comparable to fibrils width. Microscopic images from other lab confirmed this, and the authors referred to prion particles as "barrels" (Bagriantsev et al., 2008).

(2) We had to modify the Sup35 sequence beyond the prion core. As we write in the manuscript, initially we have found that the GFP globule introduces high structural noise thus making structural reconstruction impossible (Burtseva et al., 2023). One should expect the same effect from the Sup35 C domain. So, we had to remove GFP with trypsin, but then fibrils started to aggregate. To cope with aggregation, we introduced a stretch with 11 negative charges after residue 96. We provide a **Figure S5E** showing that the charged cluster did not affect the prion structure, as evident by the PK digestion pattern.

We have added a separate chapter to the Supplementary text, where we summarize all experimental data we collected to prove the relevance of the [*PSI*+]-S7 structure obtained here to the native [*PSI*+] prion polymers.

2. Are the authors sure that the use of 4% SDS during the extraction of prions does not alter their structure or disassemble a proportion of the fibrils? The authors say they monitored this process using fluorescence microscopy, but I could not find the associated Figure.

Authors:

The use of SDS was difficult to avoid since it is required for dissolving higher-order prion aggregates (**Figure S4D**) into individual fibrils. Such aggregates exist for native Sup35 prions too. Dissolution can be performed with a significantly milder detergent, Sarcosyl, but it additionally requires ultra-sonication, which is not acceptable for cryo-EM.

The evidence for SDS not altering prion structure is the following:

(1) Transformation of [*psi*-] yeast cells with the [*PSI*+]-S7 fibrils used for cryo-EM analysis produced only [*PSI*+] colonies of the strong type (**Figure S5B**).

(2) We have compared the PK digestion patterns of S7 [*PSI*+] obtained with SDS and with Sarcosyl, and did not find any significant difference (**Figure S5E**). Thus, SDS causes no more damage than Sarcosyl, though Sarcosyl may also be under suspicion. Also, we know that SDS does not dissolve Sup35 fibrils even at 4% (Kryndushkin et al., 2003).

We have added the requested fluorescent image (**Figure S4D**). It shows that Sup35-GFP aggregates, initially visible as fluorescent foci (**Figure S4C**), all were dissolved.

3. The authors state that the [PSI+]-W2 prion fibrils were essentially untwisted, precluding structure determination by cryo-EM. Could the authors show this data? Are the 2D class averages distinct from those of untwisted [PSI+]-S7 prion fibrils?

Authors: 2D class averages of [*PSI*+]-S7 fibrils (both untwisted and twisted segments) and [*PSI*+]-W2 fibrils (all segments untwisted) are shown in **Figures S6A-S6C**. Based on comparison of the fine features visible on 2D class averages (i.e., number, width, separation and relative intensities of vertical striations), we estimate the 2D class averages of untwisted segments of [*PSI*+]-S7 fibrils as clearly distinct from those of [*PSI*+]-W2 fibrils. For certain orientations, both fibril types show four vertical striations, but their intensity distributions differ markedly. For other orientations, the differences in the striation number and patterns are even more pronounced. We therefore conclude that the cross-β packing differs substantially between the S7 and W2 fibrils.

Corresponding notes were added to the article's main text.

4. The authors' hypothesis that rare fibril types in Sc4 and Sc37 may be selected for in cells could be tested using the approach they take here (i.e. extract ex vivo prions from cells with Sc4 and Sc37. This may be beyond the scope of this study, but could be discussed in the text.

Authors: We are not sure that we have understood your idea well enough. What could be tested: (1) that these prion folds appear by selection of minor fractions in Sc4 and Sc37, rather than by

structural rearrangement of the major fraction, or (2) that the prion folds obtained by yeast transformation with Sc4 and Sc37 are different from those of Sc4 and Sc37?

To the former question (1): we consider, and write in the manuscript, that strong prions most likely appear by partial refolding of the Sc4 major fraction, rather than selection of a minor fraction, and we are uncertain about Sc37.

The latter question (2) was answered by an experiment with unintended involvement of three labs. Yeast strains transformed with Sc4 and Sc37 were obtained by M. Tanaka and were given to CY King, who identified them as strong VH and weak VK [*PSI*+] variants respectively (Huang and King, 2020). A bit earlier we obtained the VH and VK [*PSI*+] variants independently generated by CY King, treated them with PK and observed digestion patterns typical of ex vivo strong and weak [*PSI*+] variants, respectively (Dergalev et al, 2019), and clearly different from the PK patterns obtained by Tanaka's lab for Sc4 and Sc37 amyloids (Ohhashi et al., 2018).

Minor comments:

1. In Fig. 1A-1C and S1A, molecular mass markers are missing for SDS-AGE and the n numbers are not reported. It would be helpful to add quantification of repeated experiments.

Authors: The situation with markers for SDD-AGE (**Figure 1B**) is complicated. Such markers, the gigantic animal muscle proteins titin (4MDa) and nebulin (700Kda) are not sold, are difficult in preparation and poorly stored. A further problem is that they work very approximately. These proteins are long and flexible, in contrast to prion particles, which have a nearly similar width and length. This makes evaluation of the prion size with such marker very approximate. For these reasons, currently the majority of works do not use markers with SDD-AGE. When we introduced SDD-AGE in 2003, it was important to evaluate the absolute size of yeast prions, so we used titin and nebulin (Kryndushkin et al., 2003). Since then, SDD-AGE is mainly used to confirm the presence of prions or compare their relative size.

We have added n numbers and/or error bars to the experimental figures with quantification (in places where they were initially missing). We do not know what can be added to Figure 1A, since it just demonstrates the difference in the colony color phenotype between strong and weak prion variants.

2. Fig S1B is not cited in order.

Authors: Thank you, corrected

3. Fig 2D is cited erroneously for Fig 1G.

Authors: Corrected

4. Core1, Core1A and Core 1B are not defined in the Results text.

Authors: We have added definitions for the Core1, Core1A and Core1B to the Results (lines 140-145).

5. In Fig S4C it is not possible to evaluate the twist of the fibrils due to the particle coordinates. Could images with and without coordinates be shown?

Authors: We modified the corresponding image (**Figure S6A**) as you suggested.

6. The conclusions that, "These segments displayed similar 2D class features apart from the degree of twist, indicating a common molecular fold", could be further tested by producing untwisted projections of the [PSI+]-S7 structure, as performed in https://www.nature.com/articles/s41586-022-05319-3.

Authors: We have performed the suggested analysis/computation and have found that the untwisted projections, obtained from [*PSI*+]-S7 atomic model, display very close resemblance to corresponding experimental 2D class averages obtained from cryo-EM images (**Figure S7D**), supporting our initial

suggestion that both untwisted and twisted segments of [*PSI*+]-S7 fibrils are likely to share the same core structure.
We have also made the untwisted back-projections from the Sc37 atomic model reported by Tanaka et al., 2025 to make a more correct comparison with the experimental 2D class averages of untwisted [*PSI*+]-W2 prion fibrils (**Figure S7E**).

7. Are the authors sure that their fibril structure has a left-handed twist? This could be confirmed by examining densities for peptide group oxygens, which should be visible in their high-resolution reconstruction, as performed here, https://www.nature.com/articles/s41586-021-04199-3.

Authors: We added the validation of helical handedness according to your suggestion (see the **Figure S6B** and the corresponding description in the Supplementary text).

8. The statement "it appears likely that only single-filament fibrils could succeed as viable prions in yeasts" is not evident from the evidence, do the authors have supporting evidence?

Authors: This statement is a hypothesis, a theoretical prediction. Our S7 and W2 ex-vivo preparations are the only ones ever studied thoroughly enough to conclude the absence of double fibrils in them. In 1998, we (Kushnirov and Ter-Avanesyan, 1998) were the first to propose the correct explanation for how yeast prions multiply through their fragmentation by Hsp104. This explanation relies on prion fibers being single-filament. So, it was tempting to develop this theory further, to consider multi-filament amyloids. However, the original text lacked a proper conclusion, which made the text less meaningful, and we have added such conclusion to the revised text. It is known that many human amyloids are composed of two or more filaments. So, why the yeast prions are different? This is because their propagation through cellular generations requires frequent fragmentation by Hsp104, which, in turn, can only be effective with single filaments. There is no such requirement in the case of human amyloids, so they can be multi-filament.
